# Prenatal Docosahexaenoic Acid Supplementation and Offspring Development at 18 Months: Randomized Controlled Trial

**DOI:** 10.1371/journal.pone.0120065

**Published:** 2015-08-11

**Authors:** Usha Ramakrishnan, Amanda Stinger, Ann M. DiGirolamo, Reynaldo Martorell, Lynnette M. Neufeld, Juan A. Rivera, Lourdes Schnaas, Aryeh D. Stein, Meng Wang

**Affiliations:** 1 Emory University, Atlanta, Georgia, United States of America; 2 University of Washington, Seattle, Washington, United States of America; 3 Georgia State University, Atlanta, Georgia, United States of America; 4 Global Alliance for Improved Nutrition (GAIN), Geneva, Switzerland; 5 Instituto Nacional de Salud Publica, Cuernavaca, Mexico; 6 National Institute of Perinatology, Mexico City, Mexico; 7 Family Health International 360, Durham, North Carolina, United States of America; Institute of Preventive Medicine, DENMARK

## Abstract

**Objective:**

We evaluated the effects of prenatal docosahexaenoic acid (DHA) supplementation on offspring development at 18 months of age.

**Design:**

Randomized placebo double-blind controlled trial.

**Settings:**

Cuernavaca, Mexico.

**Participants and Methods:**

We followed up offspring (n = 730; 75% of the birth cohort) of women in Mexico who participated in a trial of DHA supplementation during the latter half of pregnancy. We assessed the effect of the intervention on child development and the potential modifying effects of gravidity, gender, SES, and quality of the home environment.

**Interventions or Main Exposures:**

400 mg/day of algal DHA.

**Outcome Measures:**

Child development at 18 months of age measured using the Spanish version of the Bayley Scales of Infant Development-II. We calculated standardized psychomotor and mental development indices, and behavior rating scale scores.

**Results:**

Intent-to-treat differences (DHA-control) were: Psychomotor Developmental Index -0.90 (95% CI: -2.35, 0.56), Mental Developmental Index -0.26 (95% CI: -1.63, 1.10) and Behavior Rating Scale -0.01 (95% CI: -0.95, 0.94). Prenatal DHA intake attenuated the positive association between home environment and psychomotor development index observed in the control group (p for interaction = 0.03) suggesting potential benefits for children living in home environments characterized by reduced caregiver interactions and opportunities for early childhood stimulation.

**Conclusions:**

Prenatal DHA supplementation in a population with low intakes of DHA had no effects on offspring development at 18 months of age although there may be some benefit for infants from poor quality home environments.

**Trial Registration:**

Clinicaltrials.gov NCT00646360

## Introduction

The long-chain polyunsaturated fatty acids (LCPUFAs) docosahexaenoic acid (DHA) and arachidonic acid (AA) comprise more than 30% of the phospholipid content of the brain and retina and accumulate during the brain growth spurts that occur in the latter half of pregnancy and early childhood [1–3]. DHA is especially concentrated in the nonmyelin membranes of the brain and retina, and inadequate DHA intake during early life may be associated with changes in both structural and functional development of visual-sensory, perceptual, and cognitive systems [4–6]. The need to provide preformed DHA during pregnancy has received much attention since dietary intakes are low in many populations, and the primary sources, namely fish such as tuna, may be contaminated with heavy, neurotoxic elements like mercury [7][8]. Although in humans DHA can be synthesized from the essential parent n-3 fatty acid, alpha linoleic acid (ALA), recent work suggests that the rate of conversion is insufficient to meet the increased demands of pregnancy and infancy [9,10]. The n-3:n-6 fatty acid ratio in the diet and prevalence of the genetic polymorphisms for the rate limiting enzymes (fatty acid desaturases 1 and 2) that catalyze the conversion of ALA to DHA may influence the amount of DHA required during pregnancy [11].

Experimental animal studies and observational human studies have shown positive associations between intakes during pregnancy of the n-3 LCPUFAs eicosapentanoic acid (EPA) and DHA and a range of measures of offspring neurodevelopment [12–17]. The published randomized controlled trials (RCTs) examining the effects of prenatal supplementation on child development have used fish oil supplements containing both EPA and DHA and have reported inconsistent effects on infant cognitive development [18–25]. Several recent meta-analyses and systematic reviews examining the effects of LCPUFA supplementation during pregnancy and/or lactation revealed no differences on infant neurodevelopment outcomes; a major limitation identified by the authors was the heterogeneity among studies regarding the timing, type, concentration and duration of LCPUFA supplementation and methods used to assess the outcomes [26–30]. Further, few studies have examined the benefit of providing DHA without EPA during gestation especially in low-middle income countries. This gap was the rationale for Prenatal Omega 3 Supplementation on child Growth and Development (POSGRAD), which was conducted in Mexico from 2005–2007 [31]. In POSGRAD, we investigated the benefits of providing only DHA from an algal source from 18–22 weeks gestation to parturition (clinical trial registration: NCT00646360) in a population with very low intakes of preformed DHA (median intakes = 55 mg/d) [32]. We have previously reported that DHA improved birth size only among primigravid women [31], and the intervention was effective in improving maternal DHA status; women who received prenatal DHA had higher maternal plasma DHA concentrations at delivery and 1 month postpartum and breast milk DHA concentration at 1 month postpartum compared to women who were assigned to placebo [33]. In this paper, we present the findings for offspring development measured by the Bayley Scales of Infant Development-II (BSID-II) at 18 months of age. We hypothesized that prenatal DHA supplementation would improve scores on the BSID-II for the offspring at 18 months.

## Methods

### Study population and setting

Between February 2005 and February 2007, pregnant women were recruited during routine prenatal care visits at the Mexican Institute of Social Security (Instituto Mexicano del Seguro Social [IMSS]) General Hospital I and three associated health clinics, all located in Cuernavaca, Mexico. Women are eligible for care at the hospital if they or their husbands are employed. Women who were in gestation week 18–22, age 18–35 years, planned to deliver at the IMSS General Hospital and to remain in the area for the next 2 years, and planned predominant breastfeeding for at least 3 months were eligible to participate in the study. Women were excluded from the study if they were considered a high risk pregnancy, had any lipid metabolism/absorption conditions, regularly took DHA or fish oil supplements, or used certain chronic medications (such as antiepileptic drugs).

The Emory University Institutional Review Board and the Research, Biosecurity and Ethics Commissions of the Instituto Nacional de Salud Pública (INSP) approved the study on Feb 28, 2004. After a thorough explanation of study details, written informed consent was obtained from each woman. An external data safety committee monitored the trial for adverse events. The clinical trial was registered in clinicaltrials.gov (NCT00646360) in 2008 when registration of clinical trials started to become common practice; this was after participants were already enrolled. The authors confirm that all ongoing and related trials for this intervention are now registered.

### Sample Size

As described previously [31], we estimated that a final sample of 338 infants per group would have at least 90% power to detect an effect size of 0.25 Standard Deviation (SD) units or greater for the major outcomes at the end of the study, assuming a significance level of α = .05 for a two-tailed test. We therefore planned to recruit at least 994 pregnancies, assuming a 15% loss to follow-up during pregnancy and a further 20% loss in infancy, to have 393 births and 338 mother–child pairs per group complete the study at 18 months of age. The final analytic sample of 365 children per group at age 18 mo was higher than expected and gave us at least 95% power to detect a difference of at least 0.25 SD as planned [34].

### Intervention

Women were assigned randomly to receive 2 capsules of 200 mg of DHA or placebo from weeks 18 to 22 of gestation through delivery. The DHA capsules were derived from an algal source (Martek Biosciences Corporation, Columbia, MD). The placebo pills contained a mix of corn and soy oils with no added antioxidants, and they were similar in appearance and taste to the DHA capsules. The amount of precursors of DHA in the placebo was negligible. Fieldworkers delivered 14 capsules weekly to the woman’s home or workplace, and compliance was monitored by counting any remaining pills and through interviews with the participants. Women were instructed to take both capsules together, at the same time each day, and supplement ingestion was discontinued at delivery.

### Randomization and Blinding

A block randomization scheme was used to create balanced replication of four treatments (two colors for DHA and two for control) using a block size of eight. The list was generated for a sample size of 1,104, and assignment codes were placed in sealed envelopes kept at Emory University. All participants and members of the study team were blinded to treatment assignment throughout the intervention period. The participants and fieldworkers in Mexico remain blinded as follow-up data are still being collected.

### Outcome Measures

We measured child development at 18 months of age using the Spanish version of the second edition of the Bayley Scales of Infant Development (BSID-II) [35–37]. The BSID-II assesses motor (fine and gross), cognitive, and behavioral development from one to forty-two months of age, and yields three scales: the Mental Scale, the Psychomotor Scale, and the Behavior Rating Scale. The Mental Scale evaluates memory, habituation, problem solving, early number concepts, generalization, classification, vocalizations, and language. The Psychomotor Scale tests both gross and fine motor movements including those associated with rolling, crawling, sitting, standing and walking and imitation of hand movements. The Behavior Rating Scale aids in interpretation of the Mental and Psychomotor Scales by assessing the child’s behavior during the testing. The BSID-II was chosen because it was recommended by experts at time of planning [38] and used widely in Mexico [36,37].

The BSID-II was administered in a quiet setting at IMSS hospital by a team of psychologists (n = 5) who were trained and supervised by the study psychologist (LS), who was involved in the development and previous applications of the Spanish version. Supervision included periodic direct observation and routine examination of all completed forms. As described in the BSID-II manual, scores were adjusted for children who were not exactly 18 months of age at time of testing. Children received credit for each item on the Mental and Psychomotor Scales. The mental and psychomotor scores were computed by adding the total number of items for which the child received credit; these scores were converted to the Mental Developmental Index (MDI) and Psychomotor Developmental Index (PDI) using the scales provided in the manual. The normative mean of each index is 100 with a standard deviation of 15 and range of 50–150; an index score between 85 and 114 is considered *Within Normal Limits* for both MDI and PDI. Children who received a score below 70 were considered as significantly delayed and those 70–84 were mildly delayed. The Behavior Rating Scale (BRS) is translated into a percentile rank, with scores ≥ 26^th^ percentile relative to age being classified as *Within Normal Limits*. Additional factor scores within the Behavior Rating Scale include *Motor Quality*, *Orientation/Engagement*, and *Emotional Regulation* [35].

### Other measures

At child age 12 months, trained field workers conducted home visits to collect information about the home environment, using a Spanish version of the Infant/Toddler Home Observations for Measurement of the Environment inventory (HOME) that has been pretested and used in previous studies in Mexico [39,40]. This widely-used measure consists of 45 yes/no questions divided into six subscales: parental responsivity, acceptance of child, organization of the environment, provision of appropriate materials, parental involvement, and variety of stimulation. HOME scores were obtained through observation and interview with the primary caregiver (usually the mother), and the sum of all six subscales was used as a continuous variable. Higher HOME scores indicate a more enriched home environment, and although no cutoff scores are specified in the manual, scores are divided into quartiles on the summary sheets. Scores falling in the lowest quartile indicate an environment that may pose some risk to child development [39]. We obtained additional information on the mother by interview at the time of recruitment [31]. Gravidity was characterized as first pregnancy or other. Socio-economic status was derived using principal component analysis of data including occupation, housing quality and asset ownership including major household goods, and was modeled as a continuous variable. Maternal intelligence was assessed using the Raven’s Progressive Matrice (RPM)s, a non-verbal assessment of cognitive ability, which requires the participant to figure out complementary abstract patterns [41]. The test measures the ability to form comparisons, reason by analogy, and to organize spatial perceptions into systematically related wholes, with higher scores signifying higher levels of maternal intelligence. The RPM has been shown to be a useful measure of generalized intelligence, especially in low-literacy societies, including Guatemala and Mexico [42,43]. A trained psychologist administered the RPM, containing 60 items presented in five sets (12 items per set) and the number of accurate responses was computed. Maternal and infant anthropometric measurements were obtained by trained field workers during study visits, and details of birth outcomes and infant feeding practices were obtained as described in earlier publications [31][44,45].

### Statistical Analysis

We computed treatment compliance as the total number of capsules consumed expressed as a percentage of the total number expected to be consumed. All children with BSID-II measurements at 18 months were included in the analysis, regardless of treatment compliance. We compared maternal characteristics at baseline and selected infant characteristics between treatment groups using Student’s t-test for comparison of means or *Χ* ^2^ tests for comparison of proportions. We also assessed inter-interviewer variability in the mean and variance of the outcome measurements. We followed an intent-to-treat approach and examined group differences in BSID-II scores, both without and with adjustment for maternal height at recruitment (which differed between DHA and placebo, p<0.05), child sex, child age at measurement and psychologist performing the test using general linear models.

We also tested (post-hoc analysis) for effect modification by gravidity, gender, SES, and HOME score using general linear models (PROC GLM). We developed multiplicative interaction terms for each of these four variables with treatment assignment, and tested whether the interaction term was significant at p<0.05 in separate models. Sensitivity analysis was also done for the overall and post-hoc testing of interactions by testing additional regression models that also adjusted for gestational age and maternal intelligence and socioeconomic status, which are known to be associated with the primary outcomes [19,46–48] for the overall sample as well as only singleton term births. SAS 9.2 (SAS Institute, Cary, NC) was used for analyses and statistical significance was defined as p≤0.05.

## Results

**Fig 1:**
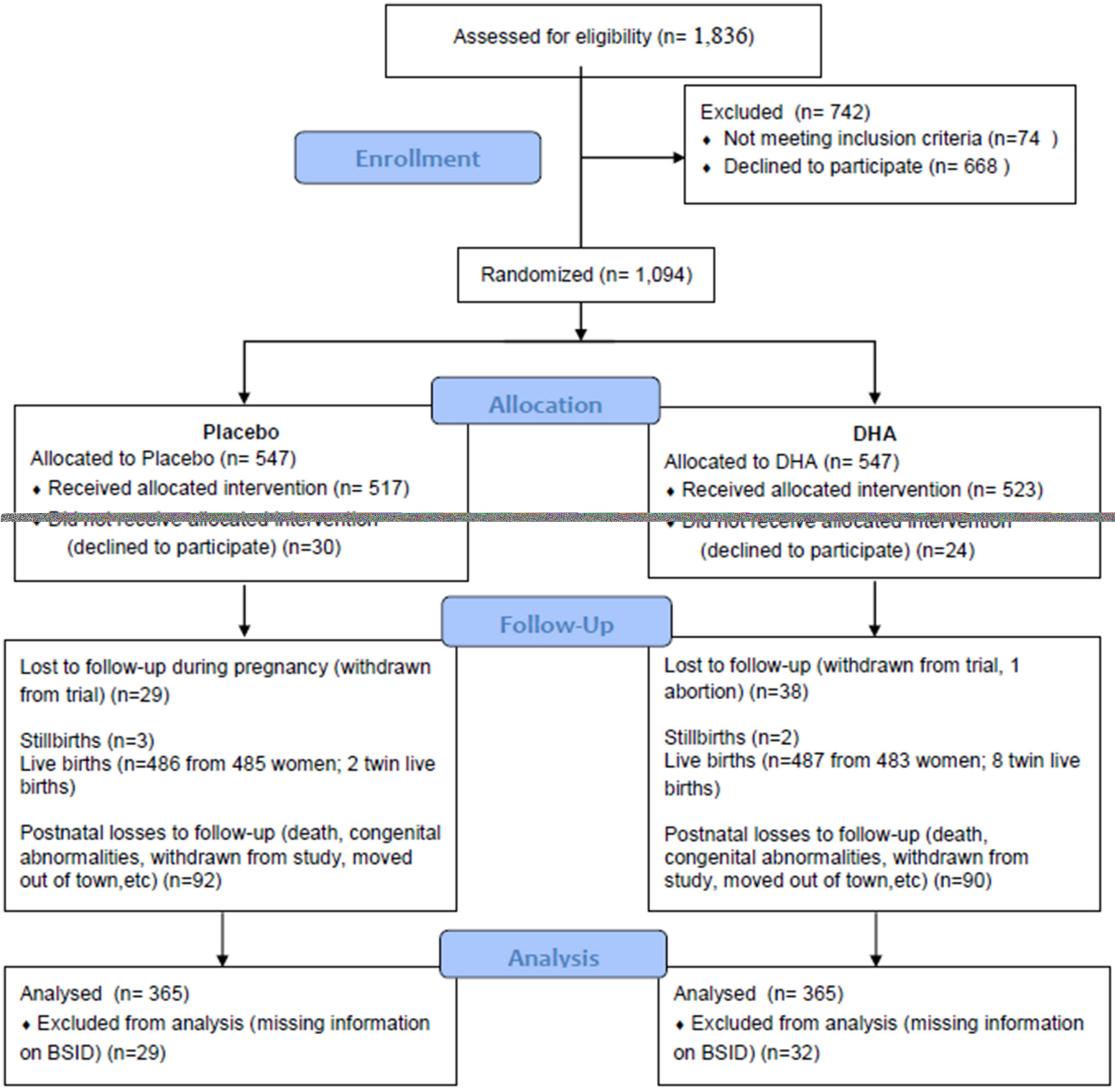
CONSORT diagram of Prenatal Omega-3 Supplementation on Child Growth and Development trial.

Of the 1836 pregnant women screened for inclusion in the study, 1094 women were eligible and agreed to participate; 968 women completed the intervention, resulting in 973 live births, including 5 twin pairs (Fig 1). A description of the intervention trial and results for birth outcomes, safety, adverse events and compliance has already been reported [31]. Compliance was high (> 90%) and similar between the two groups (p = 0.6). Loss to follow-up during pregnancy was <10%; an additional 15% were lost to follow-up during the first 18 months of life. The main reasons for loss to follow-up during the post-natal period (e.g., relocation, death, etc.) did not differ by treatment group. Comparison of the final sample with outcome data (n = 730) to those randomized but lost to follow-up (n = 364) showed that the offspring in the final sample were similar in terms of selected maternal and infant characteristics, including treatment group.

Mean maternal age at the time of recruitment was just over 26 years in both groups, and maternal socioeconomic status, schooling and intelligence did not differ among groups (Table 1). Mothers in the placebo group were taller than mothers in the DHA group (155.8 vs. 154.9 cm, p = 0.03). The prevalence of low birth weight and preterm birth did not differ between groups, nor did infant feeding practices, the HOME score at 12 months of age, or offspring weight and height at 18 months of age.

**Table 1:** Selected maternal characteristics at randomization and child characteristics among 730 children born to women who participated in a trial of 400 mg/d docosahexaenoic acid during pregnancy and had measures of infant development at 18 mo of age, by intervention group^a^.

Variables	Placebo	DHA	P^b^
	(n = 365)	(n = 365)	
Maternal characteristics at randomization			
Age (y)	26.3 (4.6)	26.5 (4.9)	0.76
Gestational age (wks)	20.5 (2.1)	20.6 (2.0)	0.71
Socio-economic status	0.0 (1.0)	0.0 (1.0)	0.81
Schooling (High school or more), %	61.2	56.8	0.23
Raven´s score	41.5 (9.1)	40.8 (8.8)	0.33
Primigravida, %	40.1	36.6	0.34
Weight (kg)	63.6 (11.5)	62.1 (10.6)	0.08
Height (cm)	155.8 (5.8)	154.9 (5.6)	0.03
Body mass index (kg/m^2^)	26.2 (4.4)	25.9 (4.0)	0.34
Child characteristics at birth			
Weight (g)	3225 (472)	3246 (432)	0.53
Length (cm)	50.4 (2.5)	50.3 (2.2)	0.65
Head circumference (cm)	34.3 (1.8)	34.5 (1.5)	0.18
Low birth weight (<2500g), %	5.2	4.1	0.48
Gestational age (wks)	39.1 (1.7)	39.1 (1.8)	0.90
Preterm (<37 wks), %	8.3	8.8	0.81
IUGR, %	9.9	10.7	0.73
Sex (male), %	53.7	54.3	0.88
Child Characteristics during early childhood			
Breastfed at least 6 months (%)	58.1	61.9	0.30
HOME total score at 12 mo of age	36.7 (4.3)	37.0 (4.4)	0.55

Abbreviations: IUGR—Intra uterine growth retardation: birth weight for gestational age <10th.

^a^ Values are Mean (Standard Deviation) unless otherwise indicated

^b^ T-test for comparison of means and chi-square test for comparison of proportions percentile Williams reference

Intent to treat analysis showed no significant differences by treatment group for the MDI, PDI or BRS (Table 2); the results did not change after controlling for maternal height and offspring sex and age at measurement. We also found no significant differences in the proportion of offspring with values indicative of delayed performance. The proportion of children with scores <85 were 14.4 and 10.1% for MDI, 17.9 and 20.2% for PDI, and 0.6 and 0% were <26 for BRS in the DHA and placebo groups, respectively. The results did not differ when we excluded data collected by one psychologist whose measurements varied systematically from the others (data not shown).

**Table 2:** Comparison of measures of infant development using the Bayley Scales of Infant Development II (BSID II) at age 18 mo among 730 children born to women who participated in a trial of maternal supplementation with 400 mg/d docosahexaenoic acid during pregnancy, by treatment group^a^.

Outcome variables	Placebo (n = 365)	DHA (n = 365)	Unadjusted diff. (95% CL)^b^	Adjusted diff. (95% CL)^c^
Mental Development Index	95.2 (9.3)	94.3 (10.7)	-0.90(-2.35, 0.56)	-1.00(-2.42, 0.42)
Psychomotor Development Index	93.3 (9.8)	93.0 (8.9)	-0.26(-1.63, 1.10)	-0.46(-1.80, 0.88)
Behavior Rating Scale Raw Score	111.5 (6.2)	111.5 (6.7)	-0.01(-0.95, 0.94)	-0.01(-0.95, 0.93)

^a^Values are Mean (SD) unless otherwise indicated.

^b^unadjusted difference (DHA-placebo).

^c^ Difference (DHA-placebo) adjusted for maternal height, child sex, and child age at measurement.

**Fig 2:**
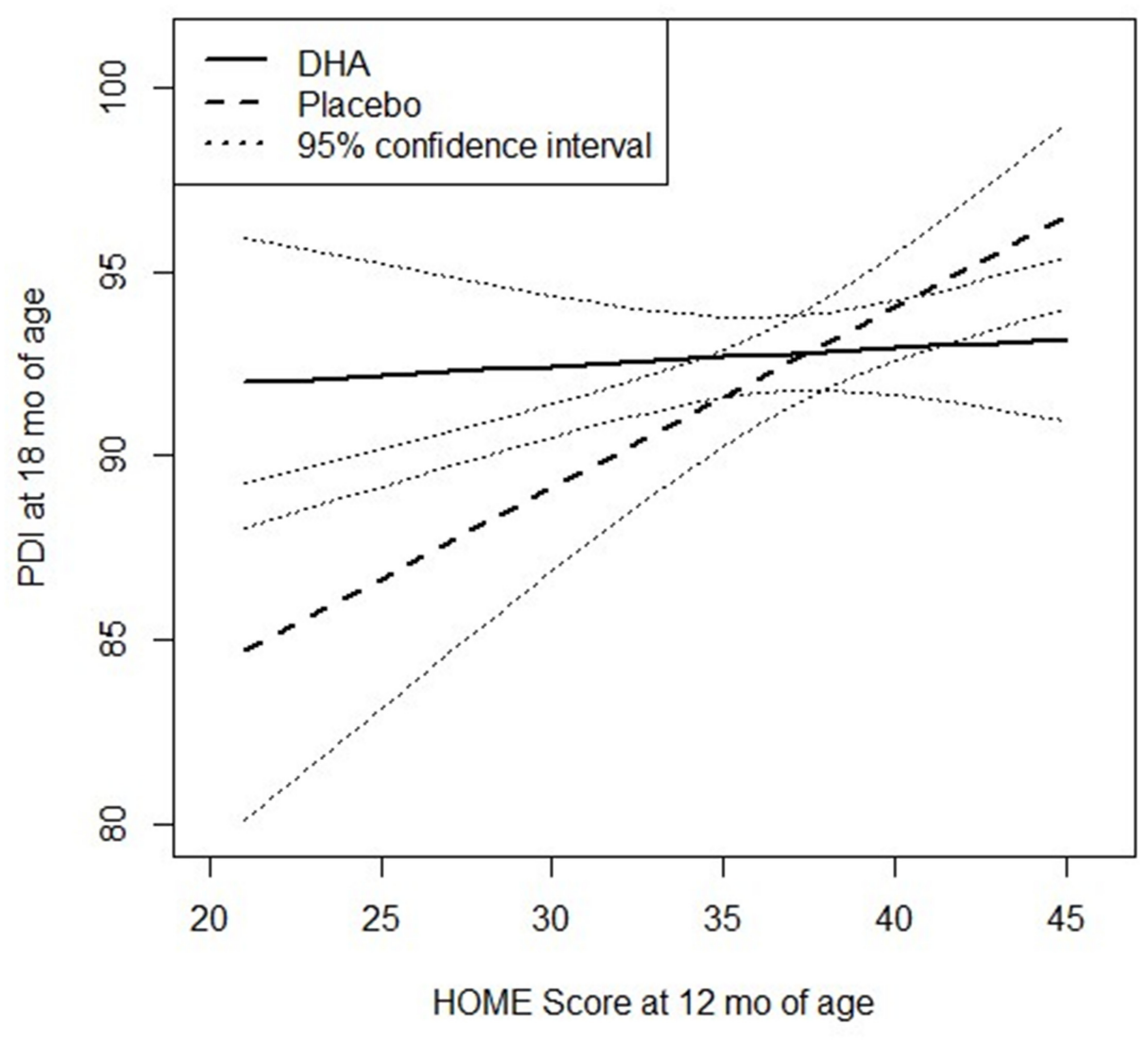
Relationship between HOME score and psychomotor development (PDI) at 18 mo of age, by intervention group.

**Fig 3:**
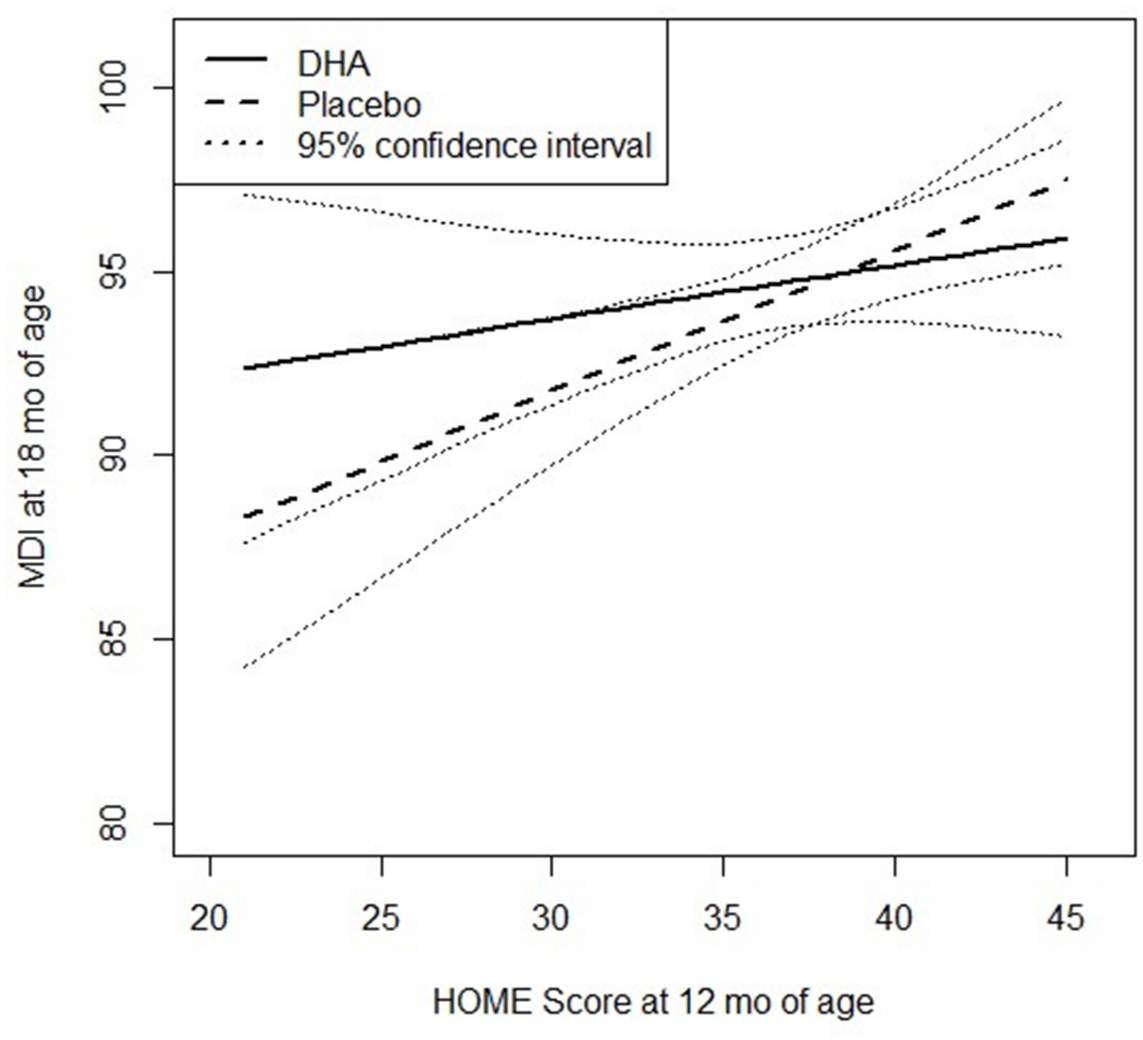
Relationship between HOME score and mental development (MDI) at 18 mo of age, by treatment group.

Post-hoc analysis revealed a strong positive association between the HOME score at 12 months of age and PDI scores measured at 18 months of age among the offspring of women who received the placebo (slope (95% CI) = 0.49 (0.23, 0.77), whereas this association was attenuated among those in the DHA group 0.05 (-0.19, 0.29); p = 0.03 for the interaction (Fig 2). The interaction between treatment and the HOME score was not statistically significant for MDI (Fig 3; p = 0.3 for the interaction). There was no effect modification by the HOME score for the BRS, nor was there evidence of effect modification by gravidity, gender or SES for PDI, MDI or BRS. The HOME score was significantly associated (p<0.01) with maternal SES and intelligence scores (the correlation coefficients were 0.15 and 0.22, respectively), but the overall findings and results of the post-hoc analysis were unaltered when we also adjusted for gestational age, maternal intelligence and SES and/or restricted the sample to singleton term infants (data not shown).

## Discussion

In this large double-blind placebo-controlled RCT providing 400mg of daily DHA supplementation during the latter half of pregnancy, we found no overall differences in infant cognitive, motor, or behavioral development, as measured by the BSID-II. Several cohort studies have shown relationships between fish intake in pregnancy and infant outcomes [7,14,17], but the results of RCTs examining n-3 fatty acid intake have been inconclusive due to heterogeneity of settings and designs [26,27,30]. Tofail et al [22] evaluated the effects of prenatal fish oil supplements in Bangladesh and found no significant differences in the BSID-II at 10 months of age. However, the power to detect differences was limited as outcomes were available for only 249 out of the 400 women who were originally assigned to treatment. Similarly, Helland et al [18] found no effect of prenatal supplementation with cod liver oil on cognitive development in a sample of 288 three-month-old children in Norway. In the much larger DOMInO trial in South Australia, Makrides and colleagues [21] did not find any differences in the overall mean scores on the Cognitive, Language, Motor or Behavior Rating Scale of the BSID-III between the intervention and control groups at 18 months of age, but in contrast to boys, girls exposed to DHA in utero had poorer mean adaptive behavior and language scores and were at increased risk of delayed language development compared to those exposed to placebo. We did not observe effect modification by gender in our study.

A major strength of this study was our ability to assess the effect of the quality of the home environment, which is a strong determinant of child development. Caregiving competence, parental responsivity, and the quality of the home learning environment are critical to a young child’s development [46,48,49]. We observed a statistically significant interaction between the quality of the infant’s home environment and treatment allocation on the PDI at 18 months, suggesting that exposure to DHA in utero attenuated the expected relationship between the quality of the home environment and PDI (but not significant with MDI). Our measures of home environment were associated with SES and caregiver characteristics such as maternal scores on the RPM, but the interaction remained significant even after adjusting for these characteristics. Though preliminary and post-hoc in nature, our findings suggest that DHA may be helpful for children living in home environments characterized by reduced caregiver interactions and opportunities for early childhood stimulation. Future work should explicitly explore the benefits of prenatal DHA supplementation for infants living in home environments that lack the stimulating environment required to promote motor and mental development, which rely on the ability of the infant to explore his/her environment [50,51].

Differences between our study and the previous literature may be due to dissimilarities in the study population, intervention, and measurements. The DOMInO trial used the updated version of the Bayley Scales of Infant Development (BSID-III), which included the language subscale that is not in the Bayley II. Additionally, the intervention group in the DOMInO trial received 800 mg of DHA and 100 mg of EPA daily, as compared to 400 mg of DHA alone in this trial. Although previous studies have used higher doses, we chose a dose that was closer to current recommendations and is considered feasible to be met by dietary interventions [8].

In our study, 181 infants (16.5%) were lost to follow-up. Loss to follow-up did not differ by intervention group and our final sample size had sufficient power (>95%) to detect meaningful differences of 3–5 points (~ 0.3 SD) in the primary outcomes of the PDI and MDI in intent-to-treat analyses. Further, the trial was conducted in a population with very low dietary intakes of n-3 fatty acids including preformed DHA (55 mg/d)[32] and the offspring exposed in utero to DHA had higher cord blood levels compared to the placebo group [31]. Postnatal factors such as infant feeding practices and home environment may have influenced the outcomes, but these did not differ by intervention group.

Finally, infant cognition and development are difficult to measure, and global standardized tests such as the BSID may not differentiate between subtle differences in infant cognitive ability [52]. The BSID is still widely used to evaluate the benefits of nutrition interventions in low to middle income settings [53,54], and was able to detect subtle differences in subgroups in post-hoc analysis. Without doubt, differences in intellectual functioning that are sensitive to pathways influenced by n-3 fatty acids may be detected with more sensitive measures of neurodevelopment such as neuroimaging techniques [55], or by measures of visual attention and executive functioning [56]. However, the suitability of these approaches for large field-based trials in resource poor settings needs further exploration. In addition, differences in cognitive functioning between those receiving a nutritional intervention and a control group may emerge later in life as reported by Helland et al. [19] and most recently by Colombo, et al. [57]. In another example, in Guatemala, protein-energy food supplementation during pregnancy and early childhood resulted in improved intellectual functioning that emerged during adolescence despite few measurable differences in offspring development during early childhood [42].

## Conclusion

This study adds to the growing body of evidence suggesting that prenatal DHA supplementation does not have a significant overall positive effect on global measures of infant development. Our post-hoc results however suggest that prenatal DHA supplementation may benefit children living in a home environment characterized by a lack of parental responsivity and a less stimulating environment. Studies that are designed to specifically examine the potential benefit of DHA for certain subpopulations using more sensitive measures of neurodevelopment are needed. The long term benefits of DHA supplementation for cognitive and behavioral outcomes that may manifest only at later ages also needs further study.

## Supporting Information

S1 CONSORT Checklist CONSORT 2010 checklist for reporting a randomised trial. (DOC)

S1 Protocol POSGRAD original research plan. (DOC)
