# Supplementary material for: Prenatal Docosahexaenoic Acid Supplementation and Offspring Development at 18 Months: Randomized Controlled Trial
Source: PLoS One. 2015 Aug 11;10(8):e0120065. doi: 10.1371/journal.pone.0120065 (PMC4532364; doi:10.1371/journal.pone.0120065)
Supplement: S1 Protocol — (DOC) [file pone.0120065.s002.doc]

**Supplemental material 1: POSGRAD original research plan**

##### **SPECIFIC AIMS** (***added specific hypothesis 3c****)*

The overall objective of this study is to evaluate the effect of docosahexaenoic acid (DHA) supplementation during pregnancy on infant growth and development. The **central hypothesis*,*** that DHA supplementation during pregnancy will improve infant growth and development, will be tested by conducting a randomized double-blinded placebo-controlled trial in Mexico.

The **specific hypotheses** are:

1. Compared to women who receive a placebo, women who receive DHA supplements during the latter half of pregnancy will have **higher DHA concentrations in breast milk and blood** at 1 and 3 months post-partum.

2. Compared to infants born to women who receive a placebo, infants born to women who receive DHA supplements during the latter half of pregnancy will have **improved birth outcomes, specifically**,

a. Greater birth weight and length and longer gestational age

b. Better neurodevelopmental indicators (Apgar and Neonatal Behavioral Assessment Scale scores)

1. Increased cord blood levels of polyunsaturated fatty acids.
   1. Compared to infants born to women who receive a placebo, infants born to women who receive DHA supplements during the latter half of pregnancy will have **better growth and neurodevelopment during the first 18 months of life**, specifically

a. Increased weight, length and head circumference at 1, 3, 6, 9, 12 and 18 months of age

- - 1. Higher Neonatal Behavioral Assessment Scale scores at 1 month of age
    2. *Improved visual and brainstem auditory evoked potentials at 1 month of age*
    3. Improved visual recognition and attention at 6 and 12 months of age
    4. Higher global motor and mental development scores at 6, 12 and 18 months of age
    5. Increased postnatal growth rates (independent of birth size)
    6. Higher global motor and mental development scores at 18 months of age (independent of differences observed at 6 and 12 months).
  1. Compared to infants born to women who receive a placebo, infants born to women who receive DHA supplements during the latter half of pregnancy will have **higher DHA concentrations in blood** at 3, 12 and 18 months of age

**B. BACKGROUND AND SIGNIFICANCE (*Revised and updated)***

**B.1. Nature of poor growth and development**

**B.1.1. Magnitude of the problem**

Poor growth and development during the prenatal period and early childhood continues to be a significant public health problem worldwide. In developing countries, nearly 200 million children suffer from growth retardation[[1]](#footnote-2). One out of 5 are born low birth weight, placing them at increased risk of death, morbidity and poor development during early childhood[[2]](#endnote-2),[[3]](#endnote-3),[[4]](#endnote-4). Nearly half of young children in South Asia are underweight, whereas 10-50% are underweight in Latin America and Sub Saharan Africa[[5]](#endnote-5). In Mexico, the latest National Nutrition Survey found that 23% of children below five years of age were stunted, with higher rates in the more economically deprived regions of the country[[6]](#endnote-6).

**B.1.2. Timing of growth and development retardation**

Pregnancy and early childhood are periods of rapid growth and development in humans. At the cellular level, neural development involves mitosis, migration, differentiation, synaptogenesis, cell death and synaptic reorganization[[7]](#endnote-7). The developmental processes overlap in time; the earliest stages of development are essentially complete before birth, some even by the beginning of the third trimester of pregnancy. Neonates use up to 60% of energy intake for growth, and synaptic connections between cells are made at a very high rate in response to light and to the need for breathing and making deliberate movements[[8]](#endnote-8). Growth and development in both motor and mental domains continue at a rapid pace during the early years and are critical for shaping intelligence, personality and social behavior,[[9]](#endnote-9) as well as for learning preparedness during the school years.

Most of the physical growth retardation in developing countries occurs primarily during the prenatal and early postnatal periods[[10]](#endnote-10),[[11]](#endnote-11). Infants in rural Mexico and Central America are born short, with an average retardation in birth length of nearly one standard deviation, and growth failure continues during the first two years of life[[12]](#endnote-12),[[13]](#endnote-13). From about 3 years of age, the linear growth velocity of developing country children is similar to that of children in developed countries[[14]](#endnote-14). The prenatal and early postnatal periods critical for normal growth and development are characterized by increased nutrient requirements, increased susceptibility to illness, and vulnerability to inadequate care[[15]](#endnote-15). Therefore, investing in effective strategies that promote growth and development during pregnancy and early childhood are needed to improve human capital and prevent life-long functional consequences, such as poor school performance[[16]](#endnote-16) and perhaps increased risk of chronic disease during adulthood[[17]](#endnote-17).

**B.1.3. Determinants of poor growth and neurodevelopment**

The causes of poor growth and neurodevelopment include poor nutrition and infection. Improving dietary intakes during pregnancy can improve birth size and reduce perinatal mortality[[18]](#endnote-18). Similarly, strategies that improve diets and reduce infections improve child growth[[19]](#endnote-19). More recently, there has been considerable interest in the role of micronutrients, especially in settings where dietary quality is poor and intakes of animal products are low. Iron, iodine and zinc improve child growth and development and the potential of multiple micronutrient supplements is also being examined[[20]](#endnote-20).

Inadequate nutrition during early childhood also disrupts cognition and intellectual functioning[[21]](#endnote-21),[[22]](#endnote-22),[[23]](#endnote-23). During the late 1960's to mid 1970's, malnutrition was believed to *directly* affect mental functioning through *biological* insult; many researchers believed that protein deficiency played a key role[[24]](#endnote-24). However, a more comprehensive understanding of how malnutrition affects cognitive development has been since proposedError: Reference source not found suggesting several routes by which malnutrition hinders cognitive development. The quality of the home environment, characteristics of the primary caregiver, and the nature of caregiver-child interactions during the early years are also critical. Intervention trials that combine food supplementation and stimulation during early childhood have demonstrated improvements in child growth and development, especially in resource poor environments[[25]](#endnote-25).

**B.2. Biological Significance of Long Chain Polyunsaturated Fatty Acids (LCPUFAs)**

Historically, the emphasis in recommendations for nutrition have centered on protein, energy, vitamin and mineral requirements, but dietary lipid requirements, especially essential fatty acids (EFA), during pregnancy and infancy have received recent attention[[26]](#endnote-26). The brain, the retina, and other neural tissues are particularly rich in long-chain polyunsaturated fatty acids (LCPUFAs)[[27]](#endnote-27). In particular, the n-3 fatty acid, docosahexaenoic acid (DHA), synthesized from alpha-linolenic acid (ALA), and found pre-formed in oil-rich fish, breast-milk and single cell organisms (Algae) is essential for the development of the fetal brain and retina.

**B.2.1. Chemistry and Nomenclature** The adult mammalian brain contains approximately 50-60% of its dry weight as lipid, mostly in the form of phospholipids, which consist of a glycerol backbone and long chain fatty acids whose amount and composition varies by phospholipid class and cell type[[28]](#endnote-28). Among the precursors of long chain fatty acids, alpha-linolenic acid (ALA, 18:3n-3) and linoleic acid (LA, 18:2n-6) represent the n-3 and n-6 fatty acid families and are called essential fatty acids (EFAs) because they cannot be synthesized **de novo** by humans and must be provided by the diet. These 18-carbon fatty acids are converted to LCPUFAs such as DHA (22:6n-3) and arachidonic acid (AA; 20:4n-6), through a series of desaturation and elongation reactions by enzymatic systems comprising fatty acyl CoA synthetases, the Δ6 and Δ5 desaturases and respective elongases, and a variety of acyltransferases and transacylases (see Figure 1)[[29]](#endnote-29). The ratio of n-3 and n-6 long chain fatty acids in the diet is critical because these two families of fatty acids compete for enzymes involved in chain elongation, desaturation, and conversion to biologically active eicosanoids[[30]](#endnote-30).

**B.2.2. Role of LCPUFAs in brain development in utero and early childhood**

The LCPUFAs, especially DHA and AA, are of critical importance in fetal and infant development: AA is distributed in membrane phospholipids throughout the body and is critically involved in second messenger, cell signaling and eicosanoid pathways, whereas DHA is found in high amounts in specific cells and membranes such as the nonmyelin membranes of the brain and retina[[31]](#endnote-31). In addition to their structural roles, these fatty acids also serve as specific precursors of eicosanoids that act as powerful autocrine and paracrine regulators of numerous cell and tissue functions[[32]](#endnote-32). Because of the fundamental roles of LCPUFAs as structural elements and functional modulators[[33]](#endnote-33), maternal, fetal, and neonatal LCPUFAs may act as important determinants of health and disease later in life. Inadequacy of LCPUFAs in critical membrane lipids may adversely affect brain growth[[34]](#endnote-34), which in turn may have functional neurodevelopmental consequences. Consequently, an adequate supply of these fatty acids to tissues prior to birth and during early life is thought to be essential for normal growthError: Reference source not found, neurological development and function[[35]](#endnote-35), as well as for learning behavior[[36]](#endnote-36). Because DHA is concentrated specifically in neural membranes of high biological activity, and because pregnant mothers’ and infants’ DHA stores are often marginal, inadequate DHA intake may be associated with changes in both structural and functional development of visual-sensory, perceptual, and cognitive systems[[37]](#endnote-37).

The human brain has its growth spurt late in pregnancy and early childhood, and accretion of LCPUFAs is particularly high during these periods[[38]](#endnote-38),[[39]](#endnote-39). The proportion of DHA in the fetal brain and retina increases steadily during the second and last trimester of pregnancy: together, DHA and AA comprise more than 30% of the phospholipid content of the brain and retina[[40]](#endnote-40),[[41]](#endnote-41). Approximately 400 mg of n-6 and 50 mg of n-3 per kg of body weight are deposited daily during late gestationError: Reference source not found. Crawford et al[[42]](#endnote-42) demonstrated that in human mid-pregnancy, as the mother utilizes her stores of DHA to support the developing fetus, her own DHA status is compromised, as evidenced by increases in mead acid (20:3n-9) and osbond acid (22:5n-6) and in the maternal DHA deficiency index (the 22:5n-6 to 22:4n-6 ratio in blood)[[43]](#endnote-43),[[44]](#endnote-44),[[45]](#endnote-45). After delivery, normalization of maternal status takes place, but recovery is slow and may still be incomplete after 6 monthsError: Reference source not found. EFAs derived from maternal diet are present in maternal plasma as triglycerides and are transported to the fetal liver by lipoprotein receptors and lipase activities in the placenta[[46]](#endnote-46),[[47]](#endnote-47). There is also evidence of selective transplacental transport of the individual fatty acids in the order DHA>ALA>LA>AA[[48]](#endnote-48),[[49]](#endnote-49),[[50]](#endnote-50),[[51]](#endnote-51). In animals, protein-calorie malnutrition during gestation and lactation affects fatty-acid Δ5 and Δ6 desaturase activities, which participate in the conversion of 18-carbon fatty acids to LCPUFAs[[52]](#endnote-52),[[53]](#endnote-53). More importantly, the human placenta does not perform elongation-desaturation of EFAs to LCPUFAs, and preformed DHA is incorporated into the developing brain and retina more efficiently than its precursor, ALA (8-fold more effective)[[54]](#endnote-54). Although body stores can be mobilized if dietary supply of n-3 and n-6 fatty acids is too low, the amounts of stored ALA (n-3) are very low compared to LA (n-6). Therefore, the diets of pregnant women should contain sufficient ALA and preformed DHA to meet both their own needs and those of the growing fetus.

**B.3. Requirements of n-3 LCPUFAs during pregnancy**

Maternal demands for n-3 LCPUFAs during pregnancy can be met by her diet, synthesis from EFA, and storesError: Reference source not found,[[55]](#endnote-55). Dietary requirements have been estimated to be 100 mg/d and the current Recommended Dietary Allowance (RDA) for pregnant and lactating women is 650 mg/d for combined EPA and DHA intakes with a minimum of 300 mg/d of DHA[[56]](#endnote-56). The RDA can be met by including about 6-7 meals of **fatty** fish per week (20–62 g/meal of **fatty** fish such as halibut, mackerel, herring, and salmon) along with approximately 22–32 g/d of a vegetable oil relatively rich in ALA (flaxseed, canola, and soybean oils)[[57]](#endnote-57). However, the reliability of these estimates is a concern[[58]](#endnote-58),[[59]](#endnote-59). EFA requirements need to be adjusted for the amount of n-6 LCPUFAs in the diet, since the dietary availability of LA (the precursor of n-6 LCPUFAs) exceeds that of ALA (the precursor of n-3s), and an increased production of DHA and EPA is unlikely to occur during pregnancy. Further, the current recommendation is based on dietary intakes in developed countries where the ratio of n-6 to n-3 fatty acids is much higher than the recommended ratio of 2.3:1, and the appropriateness of these recommendations remain unclear for those living in developing countries especially in the absence of data on the functional implications of supplementing mothers during pregnancy.

**B.4. Adequacy of Dietary Intakes of n-3 LCPUFAs during pregnancy**

The intake of fat is low in many developing countries, often contributing < 10% of total calories in some countries. More importantly, dietary intake of EFAs by pregnant women is low (< 4% of total caloric intake)[[60]](#endnote-60). In developed countries, vegetarians tend to have lower intakes of PUFA, and lower DHA concentrations have been observed in blood and phospholipids of infants of vegetarians[[61]](#endnote-61). Our preliminary data show that intakes are low among Mexican women during pregnancy, especially in communities where fish is seldom consumed (see section C). The ratio of n-6/n-3 fatty acids (~ 10) is also sub-optimal. Another concern in these diets is the increased consumption of trans-fatty acids due to the high consumption of cheap industrialized processed foods[[62]](#endnote-62). Malnourished women in developing countries may also have low adipose stores, which places them at risk for low EFA stores. Low circulating levels of LCPUFAs have also been found in pregnant and lactating women, suggesting preferential transfer to the fetus and infant by the placenta and/or mammary gland at the expense of maternal stores[[63]](#endnote-63).

**B.5. Conceptual Framework** *(revised and updated)*

The potential effects of in-utero exposure to PUFAs on birth outcomes and infant growth and neurodevelopment are shown in Figure 2. The evidence supporting the various pathways is reviewed in the following sections.

**Figure 2: Conceptual framework relating in-utero exposure to PUFAs and infant growth and development.**

Maternal PUFA status

Maternal diet in pregnancy

Infant PUFA status

**Infant neurodevelopment:**

Sensory, motor, cognitive, motivation and arousal related, social.

Prenatal growth

Fatty acid composition of developing neural tissues and retina

***+ -- weak association in human studies; ++ -- strong association in human studies***

**** -- weak association in animal studies; ** -- strong association in animal studies***

**B.5.1 The Effects of Maternal Diet during Pregnancy on Maternal and Neonatal Fatty Acid Status**

Infant PUFA status is highly correlated with maternal status[[64]](#endnote-64),[[65]](#endnote-65),[[66]](#endnote-66). Between early pregnancy (up to week 10) and delivery of the infant, maternal AA and DHA levels fall by an average of 23% and 52%, respectivelyError: Reference source not found. An inverse relationship between DHA plasma levels and number of previous pregnancies[[67]](#endnote-67) suggests that short interpregnancy intervals may tend to deplete maternal DHA stores. Three experimental studies have shown that supplementation of pregnant women with fish oil influences both maternal and neonatal fatty acid status[[68]](#endnote-68),[[69]](#endnote-69),[[70]](#endnote-70). Van Howellingen et al supplemented healthy pregnant Dutch women (n=533) with 2.7g of fish oil/day (0.9 g of DHA and 1.3 g of EPA) from the 31^st^ week of pregnancy until delivery and found significantly higher levels of n-3 LCPUFA in cord blood and walls and in maternal plasma phospholipids. There was also a comparable reduction in the amount of long chain n-6 fatty acids that compete for the same enzyme complex. In the second studyError: Reference source not found, 15 healthy pregnant women received n-3 fatty acid supplementation (2.6 g/d, of which 1.01 g was DHA) from sardines and additional fish oil during late pregnancy (26 - 35^th^ week of gestation). Maternal and newborn plasma levels of DHA were about 1.5 times higher at delivery in the supplemented group compared to the unsupplemented group. Similar results were observed for DHA concentrations in red blood cellsError: Reference source not found. More recently, Velzing-Aarts et al (2001)Error: Reference source not found found that lower doses of n-3 fatty acids (0.5- 1 g,) during the second half of pregnancy either as a milk based supplement (185 mg DHA/d) or fish oil capsules (369 mg DHA/d) were also effective in increasing cord blood PUFA levels compared to controls. *Otto et al (2000)**[[71]](#endnote-71) also found significant increases in maternal DHA status after just 4 weeks of supplementation with 0.57g/d DHA derived from an algal source. Finally, Smuts et al (2003)**[[72]](#endnote-72) found significant increases in infant RBC DHA following supplementation of maternal diets during pregnancy with DHA enriched eggs. It is important to note that no adverse effects were observed in the studies that used only DHA; our study involves only DHA.*

**B.5.2 Effects of Maternal Fatty acid status during pregnancy on intra-uterine growth and gestational age**

Maternal and infant fatty acid status at birth is positively associated with birth size (weight, length, head circumference) and gestational age[[73]](#endnote-73),[[74]](#endnote-74),[[75]](#endnote-75) including among premature and low birth weight (LBW) infants[[76]](#endnote-76),[[77]](#endnote-77),[[78]](#endnote-78). Rump et al (2001)[[79]](#endnote-79) reported an inverse association between birth weight SD scores for gestational age and AA and DHA levels in umbilical cord plasma phospholipids in a study of 627 full-term infants but a positive association between the intermediate n-6 metabolite dihomo-gamma linoleic acid (20:3n-6) and birth weight, suggesting possible differential effects of PUFAs on cell growth. Grandjean et al (2001)[[80]](#endnote-80) also found that a 1% increase in cord blood EPA was significantly associated with a 246 g decrease (95% Confidence Interval CI:16-476) in birth weight adjusted for gestational age, whereas cord blood DHA was positively associated with increased gestational age in a population of women who had high intakes of marine products. Few prospective studies have examined the relationship between maternal dietary intakes during pregnancy and birth outcomes. Olsen et al (1991) found that n-3 fatty acids in maternal RBC were positively associated with gestational age among Danish women (increase of 5.7 days in gestation was also associated with a 20% increase in maternal erythrocyte n-3/n-6 ratio) but not in Faroese women who had higher intakes of n-3 fatty acids, suggesting possible saturation effects[[81]](#endnote-81). In a study of women with low fish intakes but high intakes of n-6 fatty acids, maternal plasma and RBC AA levels measured at 34 weeks gestation and at delivery were dramatically higher in preterm cases compared to controls[[82]](#endnote-82). In a prospective longitudinal study of 372 pregnant Dutch women maternal intakes of n-3 fatty acids plus AA and riboflavin were positively associated with fetal growth[[83]](#endnote-83). However, an inverse relation between maternal LA intake and neonatal head circumference suggests that a high ratio of dietary LA (n-6) to n-3 fatty acids is inversely associated with head circumferenceError: Reference source not found^,Error: Reference source not found^. *Olsen and Secher (2002)[[84]](#endnote-84) also found strong associations between seafood consumption during pregnancy and birth outcomes. Women who never consumed fish (< 0.15g/d of n-3 LCPUFAs) had adjusted odds ratio (AOR) of ~3.6 for both preterm delivery and LBW when compared to women who consumed fish at least weekly.* Finally, Elias and Innis (2001)[[85]](#endnote-85) recently reported that gestational age and birth weight were not related to maternal plasma phospholipid fatty acids measured at 35 weeks gestation, but correlated positively with AA cord blood concentrations in full term infants. Interestingly, these authors found that maternal total trans fatty acids in cholesteryl esters and tryaglycerols were inversely correlated with length of gestation but not with birth weight, suggesting either potential inverse associations between maternal dietary intakes of trans and essential fatty acids and/or the metabolic suppression of EFA desaturation by trans isomersError: Reference source not found.

Few controlled supplementation trials have been carried out and the findings are inconsistent. Most used fish-oil supplements that provide both DHA and EPA and none have been conducted in developing countries where intakes are lower and growth retardation is more common and severe. Olsen and Secher (1990)[[86]](#endnote-86) reported a 20% reduction (from 24% to 20%) in prematurity among women receiving fish oil (0.1 g of long chain n-3 fatty acids) compared to a control group. In a subsequent double-blind randomized controlled trial (RCT) of 533 healthy Danish women, infants of supplemented mothers (four 1 g Pikasol capsules containing 0.9g of DHA and 1.3 g of EPA/d from 30 weeks) had 4.0 days longer gestational age than those born to mothers receiving olive oil[[87]](#endnote-87). Infants from mothers supplemented with fish oil were 107 g heavier, mostly attributed to the longer gestation. The effect of fish oil was greatest among women with low fish intake at entry. There were however no significant differences between those who received fish-oil and a group receiving no supplement. Two later trials in Scandinavia[[88]](#endnote-88),[[89]](#endnote-89) failed to detect any significant differences in gestational age or birth weight.

Mixed findings have also been reported for fish-oil supplementation trials conducted among high-risk pregnancies. Onwude et al (1995)[[90]](#endnote-90) failed to detect any differences in gestational age in a double-blind *RCT* of women (n=232) at high risk for pregnancy induced hypertension (PIH) or intra-uterine growth retardation (IUGR). The intervention group received fish oil supplements (1.08 g of DHA; 1.62 g of EPA) while the control group received matching air-filled capsules from 19-26 wks gestation. In contrast, fish-oil supplements providing 0.9g/d of DHA and 1.3 g/d of EPA from 20 weeks gestation to delivery significantly reduced the risks of recurrent preterm birth (33% vs. 21%; Odds Ratio 0.54, 95% CI: 0.30-0.98) compared to olive oil, in a series of controlled trials of high-risk pregnancies (N=232 for preterm delivery, N=280 for IUGR, and N=386 for PIH)[[91]](#endnote-91). There was no difference in the recurrence risk of IUGR. Results from therapeutic trials in which women at risk of pre-eclampsia (n=79) or IUGR (n=63) received 6.1 g of n-3 LCPUFA (2.1 g of DHA; 2.9 g of EPA), beginning at 33 weeks of gestation also failed to show any differences in mean duration of gestation or birth weight adjusted for gestational age.

***Possible adverse effects of fish-oil supplementation****:* Animal studies using fish oil supplements have suggested prolonged bleeding and/or difficult labor. Although the majority of human trials have shown that fish oils are safe, Olsen et al (1992)Error: Reference source not found reported a tendency (non significant, p=0.1) for greater maternal blood loss at birth for those receiving fish oil compared to olive oil or no-oil supplementation. These adverse effects, if they exist, may be due to the stronger inhibitory effect of EPA, the predominant n-3 fatty acid in marine oils, on platelet aggregation[[92]](#endnote-92). Known disadvantages of fish oil supplements include the unpleasant taste and minor side effects such as belching. Studies in infants also suggest that high levels of EPA are growth inhibitory[[93]](#endnote-93),[[94]](#endnote-94),[[95]](#endnote-95) and therefore it is preferable to use supplements that contain low or no EPA. *More recent studies that have used only DHA that are not derived from marine sources have not shown any adverse effects.*

*Two studies**[[96]](#endnote-96),Error: Reference source not found have examined the benefits and safety of using DHA only during pregnancy using DHA enriched eggs.* Borod et al (1999)Error: Reference source not found conducted a RCT, in which 53 pregnant women received either DHA enriched eggs (mean DHA intake= 200 mg/d) or typical eggs starting in the second half of pregnancy. Prevalence of LBW (0% vs. 26%) and preterm infants (6% vs. 26%) was lower, placentas were larger (760 g vs. 663 g), less gestational diabetes, and fewer births delivered by cesarean. Despite small sample size, these findings were statistically significant. *More recently, Smuts et al (2003)Error: Reference source not found completed an RCT in which women were recruited between the 24 to 28^th^ week of gestation and received either DHA enriched eggs (mean DHA content = 133 mg/egg) or ordinary eggs (mean DHA content = 33 mg/egg) until delivery. This study found a significant increase in the length of gestation( 6 + 2.3 days) in the higher DHA group. Birth weights were also larger in the intervention group (103 + 64 g) but this increase was not statistically significant (p=.108) because of small sample sizes (150 per group). Most importantly, this study carefully followed all women for adverse outcomes and found no safety concerns.*

**B.5.3. Effects of Maternal Diet during Pregnancy on Fatty Acid Composition of the Brain and Retina, and Neurodevelopment in their Offspring**

The fatty acid composition of developing neural tissues can be altered by changes in prenatal maternal dietary fatty acid composition in animals[[97]](#endnote-97),[[98]](#endnote-98),[[99]](#endnote-99),[[100]](#endnote-100),[[101]](#endnote-101). Studies using animal models have also shown that these changes are associated with effects on neurodevelopment, including measures of visual acuity, exploratory activity and psychomotor maturationError: Reference source not found,Error: Reference source not found,[[102]](#endnote-102),[[103]](#endnote-103). One possible underlying mechanism is that maternal dietary fatty acids during pregnancy may alter fetal brain growth cone (n-6 and n-3) fatty acids, and neurotransmitters involved in neurite extension, target finding and synaptogenesis[[104]](#endnote-104). EFAs can also play a role in accelerating myelinogenesis in the brain of rats whose mothers had been fed a lipid fraction extracted from yeast during pregnancy[[105]](#endnote-105). Few have examined this relationship in humans. According to data from one observational study in humans, full-term children (n=435) whose mothers ate oily fish during pregnancy were also more likely to achieve high-grade stereopsis at age 3.5 years than were children whose mothers did not eat oily fish (AOR: 1.57; 95% CI: 1.00, 2.45)[[106]](#endnote-106). Cheruku et al (2002)[[107]](#endnote-107) also found that infants born to mothers with higher plasma DHA levels during pregnancy (> 3% by wt of total fatty acids) had better neonatal sleep state patterning compared to those with lower levels, suggesting greater central nervous system maturity.

**B.5.4. Effects of maternal dietary intakes on breast-milk DHA levels**

Since exclusive breast-feeding is recommended for the first 6 months of life[[108]](#endnote-108), the impact of maternal dietary intakes during pregnancy and lactation on breast milk levels of DHA is of considerable interest, especially in settings where dietary intakes are poor. The milk of Fulani women, migratory nomads of the western Sahel of Africa, contained adequate proportions of ALA (0.50 + 0.16%) and AA (0.42 + 0.22%), but relatively low amounts of LA (9.95 + 2.13%) and DHA (0.15 + 0.08%) compared to the milk of various populations worldwide[[109]](#endnote-109). Similar findings have been reported from studies of breast milk composition in Pakistani and Nepali women[[110]](#endnote-110). In contrast, a recent study from Cuba[[111]](#endnote-111) reported fairly high DHA levels (0.43 + 0.26%) in breast milk and this was attributed to the regular consumption of fatty fish during pregnancy (227 g/wk) and lactation (454 g/wk) provided by the ration system. The RBC DHA levels of mothers and infants at 2 months post-partum were also high. Although there are no studies examining the relationship between maternal intakes during pregnancy and breast milk PUFA levels, supplementation trials among lactating women have shown benefits. Makrides et al (1996)[[112]](#endnote-112) supplemented lactating mothers with 0.2, 0.4, 0.9 or 1.3 g DHA/d and found that breast milk DHA levels ranged from 0.2-1.7% of total fatty acids and increased in a dose-dependent manner. Maternal plasma and erythrocyte phospholipid DHA levels were also strongly associated with dietary DHA dose. Gibson et al (1997)[[113]](#endnote-113) also reported increased breast milk concentrations of DHA following supplementation of the maternal diet for the first 12 weeks post-partum. Breast milk DHA concentration was also related in a dose dependent way to infant plasma and red blood cell levels. More recently, Jensen et al (2000)[[114]](#endnote-114) randomly assigned breast-feeding women to 3 DHA groups (170-260 mg/d) and found significant associations between the DHA content of maternal plasma and breast milk and of milk and infant phopholipids (23-41%). Similarly, Hawkes et al (2002)[[115]](#endnote-115) found that dietary supplementation of lactating women with DHA (0.3- 0.6 mg/d) significantly increased DHA levels in maternal plasma and milk. These studies indicate that breast-milk levels of DHA and therefore infant intakes of DHA can be improved by improving maternal intakes during lactation. Studies have also shown that fatty acids in breast milk originate from maternal body stores[[116]](#endnote-116),[[117]](#endnote-117), suggesting that breast milk levels may be influenced by modifying the fatty acid composition of maternal fat stores that are laid down during pregnancy.

##### **B.5.5. Role of PUFAs in child growth and development**

Only one study has examined the impact of maternal dietary intakes of PUFAs on infant growth and development. Helland et al (2001)Error: Reference source not found conducted a RCT in Norway in which 341 healthy pregnant women received either 10 ml of cod liver or corn oil daily from 17-19 weeks pregnancy until 3 months postpartum and were followed-up for birth outcomes and infant growth and development until 1year of age. Although maternal and infant PUFA status improved, contrary to expectations, there were no significant differences in birth outcomes or infant growth at 1 year of age. Similarly, there were no significant differences in measures of visual acuity at 6 and 9 months, but other global measures of motor and mental development were not examined. *However, a recent report from these researchers finds significant differences in mental development at 4 years of age, indicating the importance of DHA intakes during the pregnancy for later cognition[[118]](#endnote-118). It is likely that these effects would be greater if the study were conducted in a population at greater risk of poor growth and development than the Norwegian population studied..*

In contrast to the dearth of studies examining the relationship between maternal intakes during pregnancy and infant growth and neurodevelopment, there is considerable evidence of a relationship between infant intakes and neurodevelopment. Infants fed breast milk, which contains LCPUFA, have higher levels in erythrocytes and cerebral neurocortex than infants fed infant formula without these fatty acids and these changes may affect the timing or quality of neural development and/or alter neuronal membrane function, which in turn may affect visual and cognitive development[[119]](#endnote-119),[[120]](#endnote-120),[[121]](#endnote-121). A recent review concluded that dietary supplementation with marine oil or single cell-oil sources of LCPUFA results in increased blood levels of DHA and AA and an associated improvement in visual function in formula-fed infants matching that of human breast-fed infants[[122]](#endnote-122). A meta-analysis of trials in which preterm infants received formulas supplemented with LCPUFAs also showed significant improvements in visual acuities at 2 and 4 months of corrected age[[123]](#endnote-123). Among malnourished infants in Argentina, a nutrient formula enriched with LCPUFAs of n-6 and n-3 series achieved an erythrocyte fatty acid pattern and visual function similar to that observed in breast-fed infants[[124]](#endnote-124). The effect of supplementation on visual acuity in full term infants however is less clear[[125]](#endnote-125) and fewer studies have examined global measures of motor and mental development that may serve as better predictors of later intellectual functioning. Agostoni et al (1995)[[126]](#endnote-126) reported a benefit of supplementation in the development quotient (using the Brunet-Lezine test) at 4 months of age, whereas two small studies from Australia failed to detect any benefits of supplementation for growth or development (using the Bayley Scales of Infant Development -BSID) in full-term infants at one year[[127]](#endnote-127),[[128]](#endnote-128). Similarly, Scott et al (1997)[[129]](#endnote-129) did not find significant differences in language development among US infants at 1 and 3 years of age. In the only study with adequate power to detect small differences, general development at 18 months was similar among infants (n=300) who received the supplement (0.32% DHA) and control formula (1.1% ALA)[[130]](#endnote-130). In contrast, Birch et al (2000)[[131]](#endnote-131) recently found that supplementation of full term infants with DHA and AA, was associated with an increase of seven points in the Mental Development Index (MDI) of the Bayley Scales of Infant Development-II at 18 months of age. Further, plasma and RBC DHA at 4 months (but not at 12 months) were associated with MDI at 18 months of age, suggesting that early dietary supply of DHA is important. Birch et al (2002)[[132]](#endnote-132) also recently reported improved visual acuity among healthy term infants who received LCPUFA supplementation after 6 weeks of age, suggesting that the critical period extends beyond early infancy. Finally, Voigt et al (2001)[[133]](#endnote-133) in a follow-up of infants who participated in a supplementation trial during early infancy found improved neurodevelopment scores at 1 year of age (differences of 5- 7 points in both psychomotor and mental development indices) among infants who received PUFA fortified formula. Although the evidence for improved growth in infants receiving DHA supplements is inconclusive, all studies have been conducted in populations where growth retardation is uncommon.

**B.6. Summary** *(revised)*

Prenatal PUFA status is thought to have important consequences on growth and function of the central nervous system and, consequently on infant neurodevelopmentError: Reference source not found*,[[134]](#endnote-134)*. *Three main findings are supported by many studies. First, there is strong evidence of an association between maternal DHA status during pregnancy and neonatal infant tissue levels. Second, no adverse effects have been reported as a result of DHA supplementation. Third, there is considerable evidence that infant tissue levels are positively associated with birth outcomes such as gestational age, birth weight, length, and head circumference.* However, little is known about the effects of DHA supplementation during pregnancy on infant growth and neurodevelopment. The functional hypothesis is that the dynamic biophysical properties of membranes are partially influenced by the nature of their phospholipid fatty acid components. The composition and balance of lipids and fatty acids are important factors in the structural integrity of membranes and the function of messengers in cell signaling systems and research has shown that dietary alterations of n-6 and n-3 series fatty acids can trigger dramatic alterations in brain lipid composition**.**

Dietary changes during pregnancy and early childhood may therefore lead to changes in the composition of cell membranes and organelles in the brain, which in turn may modify brain function through changes in cell growth, cell division, enzyme activity and distortion of cellular cytoarchitecture. There is also evidence that the effects of DHA may also be mediated by **modulating gene expression** in the developing retina and brainError: Reference source not found. Intracellular fatty acids or their metabolites regulate transcriptional activation of gene expression during adipocyte differentiation and retinal and nervous development. DHA also has significant effects on the functional maturation of the central nervous system. These findings clearly call for more studies that examine the role of improving maternal dietary intakes during pregnancy on later neurodevelopmental outcomes, especially in settings where intakes are deficient and growth and development are sub-optimal. In summary, there is strong justification for the trial we propose. The impact of DHA supplementation during pregnancy on early childhood growth and development is hypothesized to be mediated through its direct effects on growth and development during the prenatal period and indirectly via quantity and quality of PUFA stores at birth as well as the availability in breast-milk during early childhood.

**B.7. Significance of the proposed research**

The proposed research will be the first to examine the effects of in-utero exposure to DHA supplementation on growth and development during the post-natal period using a randomized controlled design in a developing country setting where intakes of LCPUFAs and DHA rich sources such as fatty fish are low. The study will provide much needed information on the functional requirements of PUFAs during pregnancy, and possibly lead to cost effective strategies for the improvement of infant growth and neurodevelopment. This research may provide the scientific evidence for making appropriate dietary recommendations and contribute to the broader understanding of the range of the effects of PUFAs for humans in prenatal and postnatal periods. Other strengths of the proposed study are the use of supplements that contain only DHA (to minimize the potential adverse effects of EPA) and the use of methodologies that take into account recent advances in the measurement of infant cognition namely: i) consideration of the specificity of LC-PUFA effects on cognition, ii) inclusion of multiple tasks and levels of measurement as outcome measures using different tests at different stages of neurodevelopment and iii) a stronger emphasis on developmental processes. Finally, this study will be carried out by an expert team of scientists who have considerable expertise in conducting intervention trials and complement each other in the fields of nutritional biochemistry, epidemiology, psychology and maternal and child health and public health nutrition (see section C).

**C. PRELIMINARY STUDIES *(updated)***

The proposed study is a collaboration between the Department of International Health, Rollins School of Public Health (RSPH) at Emory University, the Instituto Nacional de Salud Publica (INSP) and the Instituto Mexicano del Seguro Social (IMSS) in Cuernavaca, Mexico. See Appendix I for letters of support. The study will be directed by a team with complementary skills and expertise in the disciplines of nutrition, maternal and child health, epidemiology and psychology. Key members of the study team are **Drs. Usha Ramakrishnan** (PI), **Reynaldo Martorell** (Co-PI), **Ann DiGirolamo** (study psychologist), **Aryeh Stein** (epidemiologist), **Huiman Barnhart** (biostatistician) and **Elena Kuklina** (post doctoral fellow in nutrition) at RSPH, **Drs. Socorro Parra-Cabrera** (Co-PI), **Juan Rivera** (Co-PI) and **Salvador-Villapando** (Co-investigator) at INSP, and **Dr. Sergio–Arturo Juárez-Márquez**  (physician) at IMSS. As a group, they have considerable experience in conducting complex epidemiological studies of pregnancy outcomes and determinants of child growth and neurodevelopment and have collaborated on earlier research projects. Relevant preliminary work by different members of the study team is described below.

**Dr. Ramakrishnan** (PI) is a nutritionist who has worked in maternal and child nutrition for the past 15 years. Her research focuses on the functional consequences of micronutrient malnutrition during pregnancy and early childhood. As part of her doctoral dissertation at Cornell University, she carried out a RCT to examine the effects of vitamin A supplementation on young child growth and morbidity in South India[[135]](#endnote-135),[[136]](#endnote-136) and received the Young Investigator Award from the Society of International Nutrition in 1993 for this work. Since joining Emory in 1994, her research has focused on interventions to reduce low birth weight and improve child growth and development[[137]](#endnote-137),[[138]](#endnote-138). Since 1997, she has been actively involved in the design and implementation of a double blind randomized controlled intervention trial examining the role of multiple-micronutrient supplements during pregnancy and early childhood on birth outcomes and growth and development during early childhood in collaboration with Dr. Juan Rivera (INSP) in a semi-rural community in Morelos State, Mexico. *Her contributions to child growth and development were recently recognized by the American Society of Clinical Nutrition who selected her as the recipient of the Norman Kretchmer Award in April 2003. A brief description of her research that is relevant to this proposal is presented here and copies of selected publications are included in Appendix II.*

Dr. Ramakrishnan and Dr. Martorell recently completed a double-blind RCT to test the efficacy of multiple micronutrient (MM) compared to iron (FE) supplements during pregnancy. Study outcomes were birth size, gestational age, maternal and infant micronutrient status, micronutrient levels in breast-milk and early postnatal growth and development. This study was supported by grants from the Thrasher Research Fund, UNICEF and Conacyt, Mexico. Data analyses are ongoing[[139]](#endnote-139),[[140]](#endnote-140),[[141]](#endnote-141),[[142]](#endnote-142),[[143]](#endnote-143),[[144]](#endnote-144). A brief description of the study and a summary of key findings are presented here. Pregnant women were recruited before 13 weeks and received supplements 6 d/wk at home until delivery. Both supplements contained 60 mg Fe, but the MM group also included 1-1.5 RDA of several micronutrients. All new pregnancies were identified using a routine home-based surveillance system in which trained field workers visited all women of reproductive age every 5 weeks. A total of 921 pregnancies were identified between July 1997 and Dec 1999, of which 873 were assigned to treatment after confirmation of pregnancy, determining eligibility and obtaining informed consent. All women received routine antenatal care at recruitment, 26, 32 and 37 wks pregnancy and 1 mo post-partum, during which venous blood samples and anthropometric measurements (height, weight (WT), mid-upper arm circumference (MAC), triceps (TSF) and subscapular skinfold thickness (SSF) measurements were obtained by trained workers. Additional data on obstetric history, sociodemographic characteristics, micronutrient status and dietary intakes were collected at baseline.

Data on birth outcomes were available for 656 pregnancies, of which birth weight and length measurements were available for 97.4% (n=639) and 92.7% (n=608), respectively. One quarter of all recruited pregnancies was lost to follow-up. More than half (60%) of these losses occurred within the first 8 weeks of supplementation and were due to early pregnancy losses (intended and unintended abortions), dislike of supplement or refusal to participate. There were few (n=27) losses to follow-up that occurred after 20 weeks of supplementation. The reasons included migration out of the study area and lack of family support. Overall, compliance with the intervention and coverage for the range of measurements whether conducted at the study headquarters and at home was extremely high (95%).

Women in both groups were similar at recruitment for age, parity, economic status, height and hemoglobin (Hb), but not for marital status (4.6% and 2.2% of single mothers in the MM and FE groups) and body mass index (23.8 + 3.9 and 24.5 + 4.3 kg/m^2^ in the MM and FE groups). Loss to follow-up (25%) and compliance (95%) were similar for both groups. Mean birth weight (MM: 2.977 + 0.376 kg; FE: 2.959 + 0.429 kg) and length (MM: 48.62 + 1.93 cm; FE: 48.61 + 1.74 cm) were similar in both groups (MM: n=329; FE: n=327). Mean weight gain during pregnancy (recruitment to 37 weeks) was greater by about 600 g in the MM group (7.70 + 3.43 kg; n=287) compared to the FE group (7.08 + 3.68 kg; n=293), but these differences were not significant after adjusting for maternal weight at recruitment. There were also no differences in changes in MAC and skinfold thickness by intervention group. However, in the case of weight changes between recruitment and 1 mo post-partum, overweight women in the MM group gained 0.33 kg whereas those in the FE group lost 0.74 kg; non-overweight women had gained 3.14 and 3.05 kg, respectively (p<0.15 for the interaction term). *Although these findings suggest that MM supplements during pregnancy do not provide any added benefit compared to standard iron, examination of dietary data suggest that energy intakes were increased in the MM group.*

In a follow-up intervention trial (Ramakrishnan, PI) funded by the Micronutrient Initiative and Conacyt (National Council of Science and Technology), Mexico, infants (n=600) born in the pregnancy study are being re-randomized to receive either multiple micronutrients or iron and vitamin A, daily from 3 to 24 months of age. Data collection on growth and development outcomes during the first 2 years of life is underway and is to be completed by the end of 2003. Many of the instruments will be useful for the proposed study (*see Appendix III*). Data on child growth, feeding practices, morbidity and development are being collected at several time points (3, 6, 9, 12, 18 and 24 months of age). In addition, data on micronutrient status and child-caregiver interaction are also being collected. Coverage rates for anthropometric measurements that are taken at home are high (>95%) at several time points among subjects who continue to receive supplementation. Coverage rates for measures of development (BSID-II) that are done at the study headquarters were around 70% at 12 and 18 months of age, with no incentives. Preliminary data indicate that at 1 year of age, 21.3% of children were stunted, *9.1% were anemic* and that approximately 9% and 17% of children have motor and mental development index scores, respectively, falling in the range of delayed development on the BSID-II[[145]](#endnote-145). Mean scores for the motor and mental development indices at 1 year of age were 88.7 + 7.9 (n=206) and 95 + 9.0 (n=207), respectively. *Preliminary analyses also show that Hb levels at 3 months of age and weight gain in the first year of life, were positive predictors of Hb levels at 1 year of age (n=230)[[146]](#endnote-146).*

Dr. Ramakrishnan’s other research interests include the prevention and control of nutritional anemias[[147]](#endnote-147), especially iron deficiency among women of reproductive age[[148]](#endnote-148),[[149]](#endnote-149),[[150]](#endnote-150), and long-term effects of early childhood nutrition. *She is leading an effort to develop training materials that will help build regional capacity in developing countries for micronutrient programs, in collaboration with the Centers for Disease Control and Prevention*. Dr. Ramakrishnan has been a member of the Nutrition Group at Emory since 1994 and has actively contributed to the design and execution of NIH funded studies *in collaboration with the Instituto de Nutricion de Centro America y Panama* (INCAP) Guatemala examining the long-term effects of malnutrition during pregnancy and early childhood on later adult outcomes, and growth and development of the next generation (PI: Dr. R.Martorell). She has contributed to several publications examining outcomes such as adult body size and composition[[151]](#endnote-151),[[152]](#endnote-152), fertility milestones[[153]](#endnote-153), intergenerational relationships in birth size[[154]](#endnote-154), and early childhood growth and development in the next generation[[155]](#endnote-155). Without doubt her familiarity with the design and methods used in prospective studies examining growth and development during pregnancy and early childhood make her highly suited to conduct the proposed study.

**Dr. Socorro Parra-Cabrera** is a nutritional epidemiologist with extensive experience with LCPUFAs. Specifically, she validated a food frequency questionnaire (FFQ) to assess the dietary intakes of PUFAs among pregnant Mexican women and found that DHA intakes correlated well with maternal RBC status[[156]](#endnote-156),[[157]](#endnote-157). She has assessed the adequacy of dietary intakes during the last trimester of pregnancy in two different settings in Mexico and found that pregnant women from urban populations had better dietary intakes of LCPUFAs, as well as almost twice the amounts of AA, EPA and DHA, when compared to those in a semi-rural community. Mean intakes of DHA were 140 and 25 mg in the urban and semi-rural populations, respectively. Interestingly, the main foodstuffs providing EFAs and PUFAs were not fatty fish, but less expensive, more culturally acceptable items such as tuna, chicken and eggs. Also, these Mexican women had much lower RBC concentrations of DHA (5.97 + 0.73 % of total fatty acids) and AA (9.49 + 2.57 % of total fatty acids) than women from Australia and Germany.

Dr. Parra-Cabrera has also conducted pilot studies of the functional effects of LCPUFAs during pregnancy and infancy on infant neurodevelopment. Her familiarity with the various indicators and methods of determining PUFA status and neurodevelopment is integral to the likely success of the proposed study. In one study, a cohort of 35 women with low risk pregnancies was recruited during the third trimester. Maternal concentrations of AA and DHA were inversely associated with reductions in measurements of Brainstem Auditory Evoked Potentials (BAEP) measured in their infants at 1 month of age[[158]](#endnote-158).

More recently, she completed data collection in a larger cohort study that examined the relationship between maternal PUFA status during pregnancy and infant growth and development. This study was funded by Conacyt, Mexico and carried out in collaboration with the Mexican Institute of Perinatology. Pregnant women (n=146) living in Mexico City were recruited during the last trimester of pregnancy and were followed up with their infants until 1 year of age. Maternal dietary intakes were assessed using the validated FFQError: Reference source not foundand fatty acid concentrations in blood and breastmilk samples were determined using gas chromatography. Infant growth and development were assessed at several time points in the first year of life and data on infant feeding practices, maternal intelligence, obstetric history and socioeconomic status were also collected using pretested instruments that are available for use in the proposed study (*see Appendix III*). Preliminary results describing the DHA status of the study population and relationships with infant development are presented below.

Table 1: Dietary Intakes of Pregnant women (n=146) in Mexico City (using a FFQ)

| **Nutrient** | **Mean** | **S.D.** |
| --- | --- | --- |
| Energy (Kcals) | 2898.62 | 770.67 |
| Protein (g) | 92.72 | 23.72 |
| Carbohydrates (g) | 414.71 | 121.40 |
| Total Fat (g) | 103.43 | 31.99 |
| Animal Fat (g) | 54.23 | 20.01 |
| Vegetable Fat (g) | 49.20 | 17.97 |
| Monosaturated fats(mg) | 38.52 | 13.77 |
| Polyunsaturated Fatty Acids (g) | 22.04 | 8.96 |
| LA 18:2n6 (g) | 14.80 | 8.92 |
| AA 20:4n6 (g) | 0.17 | 0.08 |
| GLA 18:3n3 (g) | 1.52 | 0.71 |
| EPA 20:5n3 (g) | 0.05 | 0.04 |
| **DHA 22:6n3 (g)** | **0.14** | **0.11** |
| Cholesterol (mg) | 340.61 | 165.30 |
| Vitamin C (mg) | 330.22 | 183.01 |
| Retinol (IU) | 4924.29 | 3449.39 |
| Vitamin E (mg) | 13.26 | 5.25 |

First, both dietary and biochemical markers of DHA status indicate a problem in this population. For example, mean dietary intakes of DHA during pregnancy (see Table 1) were only 0.14 g/d, in spite of adequate energy and fat intakes. The low intakes were further aggravated by an imbalance in the fatty acid intakes, namely lower intakes of n-3 fatty acids combined with relatively high intakes of n-6 fatty acids resulting in a high n—6 to n-3 fatty acid ratio which in turn can have adverse effects on lipid metabolism (they compete for the same enzyme system), and thereby on child growth and development. Data on maternal and infant fatty acid status, obtained in a subsample, corroborate the dietary data. The mean and standard deviation (S.D.) for RBC DHA concentrations (expressed as % of total fatty acid) were 6.05 + 0.85 (n=35), 0.35 + 0.27 (n=40) and 0.06 + 0.08 (n=38), in pregnant women, cord blood, and infants at 3 months of age, respectively. Maternal DHA intakes in pregnancy were positively associated with infant DHA levels at 3 months of age (r= 0.33; p=0.08, n=28), and maternal DHA levels at recruitment were predictive of cord blood DHA (r=0.31, p=0.11; n=28). Similarly, breastmilk DHA levels were also low. The mean and standard deviation for breastmilk DHA concentrations ( % of total fatty acid) were 0.15 + 0.11 (n=21) and 0.06 + 0.08 (n=30) at 15 days and 3 months postpartum, respectively. In comparison, breastmilk DHA levels were 0.23 + 0.06 in a study of US women at 2 weeks postpartumError: Reference source not found.

In terms of the main outcomes of interest, namely infant growth and development, mean (S.D) weight and length at birth were 3.18 (0.46) kg and 49.59 (2.29) cm, respectively (n=126). As expected, linear growth retardation continued in the first year of lifeError: Reference source not found (mean length for age Z score at 12 months was –0.68). Motor and mental development, measured using the Bayley Scales of development during the first year of life in the same cohort of infants, are presented in Table 2. Interscorer agreement/inter-tester reliability was above 90%. Although sample size limits our ability to make definitive conclusions, Bayley scores at 12 months of age were higher among infants whose mothers had higher intakes of DHA. Differences of 4-5 points were seen for both the mental development (MDI) and psychomotor (PDI) indices among infants whose mothers consumed at least 0.1 g of DHA in the third trimester of pregnancy, compared to those who consumed less. Linear regression analyses also show that MDI at 12 months of age increased by 18.8 points per % total fatty acids change in maternal DHA intake during pregnancy (p=0.07, n=78), adjusting for total energy and the ratio of AA (n-6) to DHA (n-3) dietary intake. Cord blood DHA was also a significant predictor of MDI at 12 months of age (regression coefficient=15.24 points/ % of total fatty acids in cord blood; p=0.03, n=21).

**Table 2: Mental (MDI) and Psychomotor (PDI) Development indices of Mexican full term infants**

| **Age**  **(mo)** | **n** | **MDI**  *Mean + S.D.* | **PDI**  *Mean +* *S.D.* |
| --- | --- | --- | --- |
| 1 | 113 | 99.15 + 5.22 | 99.55 + 7.80 |
| 3 | 112 | 98.29 + 5.20 | 98.15 + 7.20 |
| 6 | 102 | 99.46 + 6.03 | 97.04 + 9.18 |
| 12 | 97 | 101.12 + 9.01 | 93.59 + 9.33 |

Dr. Parra-Cabrera and her group evaluated visual acuity using the Acuity Teller Cards procedure. Total n-6 and n-3 fatty acids as well as the n6:n3 ratio in maternal RBC were the best predictors of better visual acuity scores; infant visual acuity at 3 months of age was also positively and significantly associated with maternal RBC levels of adrenic acid, AA/EPA ratio and AA/DHA ratio after adjusting for birth weight, length at birth and breast-feeding, but not with maternal dietary PUFA intakes*[[159]](#endnote-159)*. A study of brainstem auditory evoked potentials at one month of age detected a negative linear relationship between maternal RBC levels during last trimester of pregnancy and the latency intervals, suggesting that higher maternal levels of DHA and AA were associated with improved conduction times between the auditory stimulus and the cochlear nucleus. In addition, preliminary results also showed that exclusively breast-fed infants who had higher intakes of DHA had higher mental (7 points) and motor (3 points) development scores using the BSID-II at 12 months of age. In addition to the observational studies described above, Dr. Parra-Cabrera is starting an intervention trial in a public hospital in Mexico that will examine the impact of supplementing formula fed infants with LCPUFAs (DHA and AA) on infant growth, immune response and neurodevelopment using brain stem auditory and visual evoked potentials.

**Dr. DiGirolamo** is an Emory psychologist interested in the role of maternal depression and child-care giving for young child growth and development. *As part of a NIH funded study, she has recently completed data collection in collaboration with INSP* to test the hypothesis that caregivers who report higher levels of distress, stress, and lower levels of perceived social support will exhibit more negative interactions and caregiving behavior, which will in turn be associated with poorer growth, development, and nutritional status in young children. Interviews were conducted with the child's primary caregiver when the child is 9 and 15 months of age to assess levels of depressive symptoms, parenting stress, and perceived social support. Other measurements include in-home observations of caregiver-child interactions at 9 and 15 months, and child growth and development at 18 and 24 months of age that were collected as part of the larger micronutrient supplementation study (PI: Ramakrishnan). *Preliminary results suggest that many women (46.5%) are experiencing high levels of depressive symptoms (46.5%) and stress related to child caregiving.[[160]](#endnote-160). Analyses examining relationships between caregiver stress/distress, caregiver-child interactions, and child growth and development are currently underway. Dr. DiGirolamo is also involved in analyses of data from the larger* *micronutrient study and preliminary results show that both lower birth weight and length, and poor weight gain from birth to nine months predict poorer motor development[[161]](#endnote-161),[[162]](#endnote-162).* Her expertise in measuring maternal competence and child development especially with the Spanish version of the latest version of the Bayley Scales of Infant Development (BSID-II) will be invaluable for the proposed study.

**Dr. Aryeh D. Stein** is an epidemiologist who studies the relationships among social and geographic changes, nutrient intakes, and evolution of chronic disease risk. A general feature of this research is Dr. Stein’s attempt to isolate (and therefore be able to study) critical periods of development (e.g., gestation, infancy, puberty, adult life) in which humans may be especially sensitive to the effects of nutritional factors, whether deficits or surfeits. He joined Emory University in September 1998, becoming an active member of the team of investigators conducting the follow-up studies of the INCAP supplementation study cohort in GuatemalaError: Reference source not found,Error: Reference source not found,Error: Reference source not found,[[163]](#endnote-163),[[164]](#endnote-164). He has extensive experience in the analysis and interpretation of epidemiologic data. Specifically, his expertise in assessment of measurement error, validation of approaches to dietary intake assessment[[165]](#endnote-165),[[166]](#endnote-166),[[167]](#endnote-167) and analysis of longitudinal data[[168]](#endnote-168),[[169]](#endnote-169) is valuable for the proposed activities. Prior to joining Emory, he conducted investigations of CVD risk factor prevalence, and interrelationships between risk factors, in settings as diverse as New York State[[170]](#endnote-170),[[171]](#endnote-171) and Bulgaria[[172]](#endnote-172),[[173]](#endnote-173). He has also collaborated extensively with L.H. Lumey of Columbia University on analyses of the Dutch Famine Birth Cohort Study data set, an investigation into the long-term effects on reproductive health and birth outcomes of exposure to acute famine in utero[[174]](#endnote-174),[[175]](#endnote-175),[[176]](#endnote-176),[[177]](#endnote-177),[[178]](#endnote-178).

**Dr. Reynaldo Martorell** (Co-PI) has many years of experience with field studies in developing countries. His research has contributed to our understanding of the importance of health and nutrition during pregnancy and the first two years of life for short and long term human function. His long term follow up studies in Guatemala have led policy makers to view investment in early childhood health and nutrition as strategies for economic productivity because they improve human capital formation. Dr. Martorell has worked closely with Dr. Ramakrishnan on several projects and will advise on study logistics, analyses, interpretation and writing of results. He has also collaborated with other members of the study team and as chair of the Department of International Health (DIH, RSPH) he is especially committed to the development of strong inter-institutional linkages with INSP (see *Appendix IV*).

**Dr. Juan Rivera** (Co-PI) has considerable experience in the field of child growth and development especially in Latin America and has worked closely with the Emory team on other studies of child growth and development in Guatemala and Mexico. He has also been actively involved in the National Nutrition Survey in Mexico and evaluating the effects of large-scale nutrition interventions on child growth and micronutrient status. His expertise and guidance will be very useful to the proposed study.

Other members of the study team include Drs. Salvador Villalpando (INSP), Sergio Juárez-Márquez (IMSS), Huiman Barnhart (RSPH) and Elena Kuklina (RSPH). **Dr. Villalpando** (Co- investigator), director of basic nutrition at INSP has extensive experience in laboratory methods of nutrition assessment and has collaborated with the PI and other members of the study team in previous studies. His research in the area of fatty acid metabolism during pregnancy and lactationError: Reference source not found,[[179]](#endnote-179),[[180]](#endnote-180),[[181]](#endnote-181),[[182]](#endnote-182) and expertise in the collection and handling of biological samples and biochemical estimations of fatty acids in blood and breastmilk[[183]](#endnote-183),[[184]](#endnote-184),[[185]](#endnote-185) will be valuable for the proposed study. He has also worked at IMSS; this will further strengthen the inter-institutional linkages. **Dr**. **Juárez-Márquez** (IMSS) will work closely with the rest of the study team, especially those at INSP, in the actual implementation of the intervention trial and data collection. Both IMSS and INSP have a strong history of collaboration and have conducted collaborative research in the past (see *Appendix IV* for inter-institutional agreement between INSP and IMSS). **Dr. Barnhart;** study biostatistician, has considerable experience in analyzing datasets with longitudinal data and structural equation modeling. She has also worked closely with the Emory team on the intergenerational effects study in Guatemala. **Dr. Kuklina,** a pediatrician, is currently completing her doctoral dissertation under Dr. Ramakrishnan’s guidance on "Determinants of early childhood development in Guatemalan children" in the Nutrition and Health Sciences Program at Emory University, examining the effects of prenatal and postnatal influences on motor and mental development during early childhood using longitudinal data collected in rural Guatemala (PI: Dr. Martorell) ^Error: Reference source not found^,[[186]](#endnote-186),[[187]](#endnote-187). Preliminary results indicate that birth size and growth during the first year of life are important predictors of development during the second year of life.

Finally, the project will greatly benefit from active participation of **Dr. Ricardo Uauy and Dr. Maria Makrides**, recognized experts in the area of child growth and development and functional effects of LCPUFAs who have agreed to serve as consultants in the design, data analysis and interpretation of findings. Letters of support are included in Appendix I.

**D. RESEARCH DESIGN AND METHODS: *(revised)***

**D.1. Research Design** *(revised)*

##### **D.1.1. Study Hypotheses** *(added specific hypothesis 3c)*

The ***central hypothesis*** is that DHA supplementation during pregnancy will improve child growth and development during infancy, and will be tested by conducting a randomized double-blinded placebo-controlled trial in Mexico.

The **specific hypotheses** are:

- 1. Compared to those who receive a placebo, women who receive DHA supplements during the latter half of pregnancy will have **higher DHA concentrations in breast milk and blood** at 1 and 3 months post-partum.

2. Compared to those born to women who receive a placebo, infants born to women who receive DHA supplements during the latter half of pregnancy will have **improved birth outcomes** as follows,

a. Greater birth weight and length and longer gestational age

b. Better neurodevelopmental indicators (Apgar and Neonatal Behavioral Assessment Scale scores)

1. Increased cord blood levels of polyunsaturated fatty acids.
2. Compared to those born to women who receive a placebo, infants born to women who receive DHA supplements during the latter half of pregnancy will have **better growth and neurodevelopment during the first 18 months of life**, namely

a. Increased weight, length and head circumference at 1, 3, 6, 9, 12 and 18 months of age

- 1. Higher Neonatal Behavioral Assessment Scale scores at 1 month of age
  2. *Improved visual and brainstem auditory evoked potentials at 1 month of age*
  3. Improved visual recognition and attention at 6 and 12 months of age
  4. Higher global motor and mental development scores at 6, 12 and 18 months of age
  5. Increased postnatal growth rates independent of birth size
  6. Higher global motor and mental development scores at 18 months of age independent of differences observed at earlier time points (e.g. 6 and 12 months).

1. Compared to those born to women who receive a placebo, infants born to women who receive DHA supplements during the latter half of pregnancy will **higher DHA concentrations in blood** at 3, 12 and 18 months of age.

**D.1.2. Study Design** *(revised)*

A **randomized controlled double blind trial** will be used to test the hypothesis whether prenatal supplementation with DHA improves child growth and development. Pregnant women will be recruited at 20 weeks of gestation and will be randomly allocated to either the intervention group (daily supplements during pregnancy of 200 mg of DHA) or to the control group (a placebo that is similar in appearance and taste). The commercial preparation Neoformins PL^TM^ produced by Martek will be used. Each tablet provides 200 mg DHA synthesized from an algae source which is safer and more acceptable than the fish oils that have been used in earlier studiesError: Reference source not found,Error: Reference source not found,Error: Reference source not found. The rationale for this dosage is that it represents a physiological dose that is safe and will meet the minimum recommended intake of 300 mg/d in combination with dietary sources that are expected based on our preliminary data to contribute about 100 mg/d. *Recent studies have shown that a dose of 200 mg of DHA is as effective as higher doses in improving DHA status during pregnancyError: Reference source not found,Error: Reference source not found,Error: Reference source not found and lactationError: Reference source not found and more importantly that 200 mg of DHA is safe. Studies that have used only DHA, whether from algal sources or as DHA enriched eggs, did not find any adverse effectsError: Reference source not found,Error: Reference source not found,Error: Reference source not found. In the only study in which a tendency for increased bleeding was reportedError: Reference source not found the supplement was from marine sources and contained substantially higher amounts of DHA (nearly 2000 mg of DHA; about 10 times our proposed dose) and more importantly, high amounts of EPAError: Reference source not found. A dose of 200 mg is safe and can be provided in one pill, which would help ensure compliance. Finally if found effective, a dose of 200 mg of DHA is amenable to safe and feasible dietary recommendations for pregnant women to increase their DHA intakes using food based approaches, such as increasing the frequency of consumption of fatty fish, use of DHA enriched foods such as eggs and routine consumption of supplement.* The placebo will be similar in appearance, taste and composition to the treatment, except for DHA. Random allocation of all eligible pregnancies to either the treatment or control group will be done using a computer- generated list to be created by the study biostatistician at Emory based on an allocation ratio of 1:1 without blocking constraints[[188]](#endnote-188). All study subjects and members of the study team will be blinded to the treatment code, which will be maintained in sealed envelopes at INSP and RSPH, and opened only at the end of the study and data analysis, or made available to the external data safety monitoring committee if requested (see section E).

The inclusion and exclusion criteria for study subjects are as follows:

**Inclusion criteria**

- 20-24 weeks pregnant (based on last menstrual period)
- 18 - 35 years old
- Resident of Cuernavaca who intends to deliver at the IMSS General Hospital I and remain in the area for the next 2 years
- Intends to predominantly breastfeed infant until 3 months of age
- Agrees to participate with informed consent

**Exclusion criteria**

- High risk pregnancy: This will be as documented in the clinical record at recruitment and will include history and prevalence of abruptio placentae, any serious bleeding episode in the current pregnancy, gestational diabetes, pregnancy induced hypertension, maternal toxoplasmosis infection during pregnancy, coagulation disorders, thrombocytopenia or chronic vascular, renal or systemic disease and drug use.
- Lipid metabolism (hyperlipidemia) and/or absorption disorders
- Regular intake of fish oil or DHA supplements during pregnancy
- Chronic use of medication for illnesses like epilepsy

Informed consent will be obtained from all subjects following standard procedures (see section E) and women will be free to withdraw voluntarily at any time. All study protocols will be explained to the study subjects and will be approved by the Human Investigations Committees at Emory University and INSP. Supplements packaged in blister packs, will be delivered at home weekly by trained field workers who will also monitor compliance by interview and pill count. *Preliminary data collected from women (n=49) who had recently delivered at the IMSS Hospital in Mexico City revealed that most of them (87.5%) considered weekly supplementation at home desirable and  not invasive. Recent data from the National Nutrition Survey in Mexico have shown that only 13.8% of pregnant women reported consuming any micronutrient supplements[[189]](#endnote-189) in contrast to the high coverage rates (> 80%) that we achieved in our clinical trials in Xoxocotla with daily or weekly home visitsError: Reference source not found.* All subjects will have access to routine prenatal care. Infants will also have access to standard care and will be followed up until 18 months of age. The total duration of follow-up will therefore be 23 months for each mother-child pair.

The rationale for repeated measures of growth and development during the first 18 months of life is that frequent measurements until a later time point will more likely provide more accurate information on the dynamic process of infant growth and cognitive development. An advance attributed to the growing theoretical shift in the field of developmental psychology states that measuring the developmental course of cognitive function in infancy should be conducted within the realm of developmental systems theory[[190]](#endnote-190). Therefore, we will conduct more extensive longitudinal measurements and use measures of the developmental course of variables assessing whether nutritional supplementation affects cognitive outcome. The second methodological implication of the broader, developmental systems approach is that we will consider whether variables other than LCPUFA manipulations interact with the manipulations themselves. For example, it is possible that LCPUFA supplementation has larger effects in populations in which environmental quality (e.g., socioeconomic status or caregiver responsiveness) is poor, compared with populations in which environmental conditions are more optimal. A third implication is that small or transient effects at one age point may not be unimportant. The fact that there may be no differences in, for example, visual acuity at 6 months ignores the possibility that the acceleration of the visual acuity curve experienced by the supplemented group may have a longer-term effect. Thus, even a small early advantage in lower-order function may serve to affect higher-order functions at some later point. Further, in infancy the developmental process itself complicates the measurement of developmental functioning. For example, the Bayley may measure motor development well at 12 months, but be less valid for language development at this age as these skills are just emerging, or may be a predictor of intelligence at 24, but not at 6 months. There is also some evidence that among low-risk infants, scores within the first year of life are not predictive of later IQ, but those after 18-24 months provide more useful predictions; however, the instrument may be more predictive even within the first year of life among high-risk infants[[191]](#endnote-191). Similarly, growth faltering begins during the latter half of infancy and often continues through the second year of life. Although later prediction is not the main purpose of this study, the inclusion of these measurements will help our understanding of the underlying processes.

**D.1.3. Study Site:** *(no changes)*

This study will be carried out at the IMSS General Hospital I located in the city of Cuernavaca, Morelos, Mexico. The IMSS General Hospital I had over 9,000 women registering for prenatal care and about 3,189 deliveries in 2000. About one-third had their first visit during the first trimester, and about 60% had their first prenatal visit before week 20. The women who usually attend the hospital are medium-low socioeconomic status and either they and/or their husbands are employed. In most cases, the patient usually pays a third of the health care costs, with the Federal Government and their employer paying the other two-thirds (one third each). All pregnant women are routinely provided with tetanus toxoid vaccination and iron-folate supplementation (5 mg/day of folic acid and 100 mg of iron fumarate or sulfate) during pregnancy. Routine prenatal care will be provided by registered physicians (graduated from the Gynecological Residency) who will be assisted by registered nurses and residents. Mothers are given appointments for prenatal checkups beginning with the diagnosis of the pregnancy for each month during the 1st and 2nd trimester, and during the third trimester, every 2 weeks and during the last month, each week.  Coverage at each of these routine prenatal visits is about 70%. After birth, infants come to the hospital for monthly well baby visits in the first year of life during which they receive routine immunization and monitoring of growth and development. Infant length and weight is measured and mothers are counseled by trained nurses in a range of topics that include family planning, breast feeding, nutrition, and child development. These visits become bimonthly in the second year of life. Coverage rates for immunization are very high (>95%) for the various vaccines, and about 75% for the routine monthly visits for infants. Almost all mothers initiate breast feeding and about 60% are still predominantly breast feeding at 3 months post partum.

**D.1.4. Sample Size and Power Calculations:** *(no changes)*

**Table 3: Expected effect sizes for key study outcomes at birth and during the postnatal period**

| **Study Outcomes** | **Birth** | | | **Postnatal (3-18 mo)** | | |
| --- | --- | --- | --- | --- | --- | --- |
|  | ***S.D.*** | ***Minimum difference (d)*** | ***Effect Size*** | ***S.D*** | ***Minimum difference (d)*** | ***Effect Size*** |
| **GrowthError: Reference source not found** |  |  |  |  |  |  |
| Weight (kg) | 0.45 | 0.1 | **0.22** | 1.0 | 0.3 | **0.30** |
| Length (cm) | 1.6 | 0.5 | **0.31** | 3.0 | 1.0 | **0.33** |
| Head Circumference (cm) | 2.0 | 0.5 | **0.25** | 1.5 | 0.5 | **0.33** |
| Gestational Age (d) | 7.0 | 2.0 | **0.29** | - | - | **-** |
| **Development Error: Reference source not found***^.^****^Error: Reference source not found^*** |  |  |  |  |  |  |
| Brazelton Score | 1.0 | 0.8 | **0.80** | - | - | **-** |
| Fagan Test | - | - | **-** | 2.7 | 2.00 | **0.74** |
| Mental Development Index | - | - | **-** | 15 | 3.75 | **0.25** |
| Motor Development Index | - | - | **-** | 15 | 3.75 | **0.25** |
| **DHA statusError: Reference source not found*^,Error: Reference source not found^*** |  |  |  |  |  |  |
| Maternal blood***** | 1.0 | 0.50 | **0.5** | 1.0 | 0.50 | **0.50** |
| Infant blood***** | 1.0 | 0.50 | **0.5** | 1.0 | 0.50 | **0.50** |
| Breast milk (% TFA) | **-** | **-** | **-** | 0.08 | 0.04 | **0.50** |

**plasma phospholipid DHA levels expressed as % total fatty acids (TFA)*

Sample size calculations are based on the expected effect size, power to detect those differences and level of significance. The expected effect sizes for key outcomes presented in Table 3 were calculated by dividing the minimum meaningful difference (d) that we wish to detect by the standard deviation (S.D.) using data from either the literature or from our preliminary studies in Mexico and Guatemala. Most of the effect sizes are small to medium (0.2 to 0.5) and the minimum sample sizes required to detect them using a two-tailed test and confidence level of 0.05 are shown in Table 4 at different levels of power (80-95%)[[192]](#endnote-192). The smallest effect sizes of interest are 0.22 S.D. for birth weight and 0.25 S.D. for motor and mental development at 18 months of age. Using a two tailed test and a significance level of α=0.05, a final sample of 338 infants per group at the end of the study will have 90% power to detect an effect size of 0.25 S.D. or greater for all the major outcomes. This sample size will have 80% power to detect meaningful differences in birth weight (0.22 S.D) as we expect at least 393 births/group after accounting for loss to follow-up (see section D 1.5). The power calculations are also presented for single outcomes as well as for repeated measures of growth and development assuming correlations of 0.2. Due to budgetary constraints, blood and breast-milk samples will be obtained for a 30% sub-sample of 120 mother-infant pairs per group, which will have 95% power to detect expected effect sizes of 0.5 S.D.

**Table 4: Minimum sample sizes by effect sizes, power and number of measurements**

| **Effect**  **size** | **Minimum Sample size per Group (2 tailed test, alpha=0.05, Correlation between repeated measurements=0.2)** | | | |
| --- | --- | --- | --- | --- |
|  | 80% Power  Time Points  1 2 3 | 85% Power  Time Points  1 2 3 | 90% Power  Time Points  1 2 3 | 95% Power  Time Points  1 2 3 |
| 0.2 | 393 237 185 | 450 271 211 | 526 317 247 | 651 391 305 |
| 0.3 | 175 106 83 | 201 121 95 | 234 142 110 | 290 175 136 |
| 0.4 | 99 60 47 | 113 69 54 | 132 80 63 | 163 99 77 |
| 0.5 | 64 39 31 | 73 45 35 | 85 52 41 | 105 64 50 |

**D.1.5. Recruitment of study subjects**: *(revised)*

*The power calculations dictate that a* minimum of 676 mother-child pairs should complete the study. We expect a 15% loss to follow up during pregnancy and a further 20% loss in infancy, and therefore need to recruit at least 994 pregnancies. *These estimates are conservative and include losses due to deaths and migrations out of the study area. All efforts however will be made to keep losses to a minimum. Attendance at routine prenatal and postnatal visits is already high in our study hospital and in addition we will be providing incentives to encourage participation.* Recent data indicate that IMSS General Hospital I has approximately 250 births/month, of which ~60% are predominantly breastfed until 3 months of age. The proportion of pregnancies that are high risk and therefore ineligible for inclusion is about 10%. Based on the above data, we expect to recruit at least 100 eligible pregnancies per month and therefore to complete recruitment in 10 months. The 30% subsample of mother-infant pairs for biochemical determinations will be selected at random.

**D.1.6. Time line:** *(revised)*

The total duration of this project is *four* years and the time line for the main activities is shown in Figure 3. Activities related to the development of the protocol, pre-testing and finalizing questionnaires, hiring and training of all study staff will be carried out in the first year. The total duration of data collection will be about 32 months. Recruitment of subjects for the intervention trial will begin in the middle of the first year and will be completed in 10 months (see recruitment). The total duration of follow-up is 23 months (5 months of pregnancy and 18 months during early childhood) for each mother-child pair and therefore, all data collection will be completed early in the fourth year. Data entry and cleaning will be carried out in an on-going fashion. All lab estimations will be carried out during years 2-4. Data analysis will be conducted in the last two years and the preparation and submission of manuscripts for publication will be done in the final year.

**D.2. Data collection** *(revised)*

**D.2.1. Field operations and field team**

The details of the protocol for data collection during the prenatal and post-natal follow-up periods for each mother-child pair in the study are shown in Table 5. The study team in charge of data collection and the intervention trial will comprise of a study director who will be a physician based at IMSS General Hospital I and will report directly to the study investigators at INSP, Cuernavaca as shown in Figure 4. He/she will be assisted by area supervisors who will oversee data collection and ensure data quality. All prenatal care will be provided by trained hospital staff.

A hotline will be maintained for all study subjects to contact the study team. In terms of data collection, if we recruit about 100 pregnancies per month, we expect to have approximately 85 births per mo and approximately 70 infants/mo at 18 months of age. A team of 5-8 field workers will be hired to visit all pregnant women at home on a

**Figure 4: Organization Structure for Field Operations**

**Study Investigator (Dr. Parra)**

**Hospital Staff**

*Prenatal care and Delivery*

**Team I** (8 FW)

*Supplement Distribution*

**Team II** (*5 nurses*)

*Anthropometry*

*Dietary intakes*

*SES, Obstetric history*

*Blood samples*

*Breast-milk samples*

**Team III** *(*3 PSY)

*Maternal Intelligence Neurodevelopment*

**Team IV** (2 SW)

*Home environment*

**Field Director (Dr. Juarez)**

***Supervisor I (Physician) Supervisor II (Nutritionist) Supervisor III (Psyschologist)***

*FW – Field Workers; PSY – Pyschologists; SW- Social Workers*

Table 5: Details of data collection during prenatal and postnatal follow-up

|  | Prenatal | | | | **Birth** | | **Post-partum (mo)** | | | | | | | | | | | |
| --- | --- | --- | --- | --- | --- | --- | --- | --- | --- | --- | --- | --- | --- | --- | --- | --- | --- | --- |
|  | Baseline (20-24 wks gestation) | | **Intervention** | |  | | **1** | | **3** | | **6** | | **9** | | **12** | | **18** | |
| Sociodemographic characteristics | **X** | |  | |  | |  | |  | |  | |  | |  | |  | |
| Obstetric History | **X** | |  | |  | |  | |  | |  | |  | |  | |  | |
| Supplement consumption |  | | **X** | |  | |  | |  | |  | |  | |  | |  | |
| *Maternal diet* | **X** | |  | |  | |  | | **X** | | *X* | |  | |  | |  | |
| Maternal anthropometry | **X** | |  | |  | |  | | **X** | |  | |  | |  | |  | |
| Maternal Weight | **X** | | **X** | |  | |  | | **X** | |  | |  | |  | |  | |
| Maternal PUFA status | **X** | |  | | **X** | | **X** | | **X** | |  | |  | |  | |  | |
| Gestational age and birth outcomes |  | |  | | **X** | |  | |  | |  | |  | |  | |  | |
| Infant anthropometry |  | |  | | **X** | | **X** | | **X** | | **X** | | **X** | | **X** | | **X** | |
| Infant PUFA status |  | |  | | **X** | |  | | **X** | |  | |  | | **X** | | **X** | |
| Apgar & NBAS scores |  | |  | | **X** | | **X** | |  | |  | |  | |  | |  | |
| *Visual and Brainstem Auditory Evoked Potentials* |  | |  | |  | | *X* | |  | |  | |  | |  | |  | |
| Visual recognition memory and visual attention |  | |  | |  | |  | |  | | **X** | |  | | **X** | |  | |
| Global mental and motor development (Bayley) |  | |  | |  | |  | |  | | **X** | |  | | **X** | | **X** | |
| Infant Diet | |  | |  | |  | | X | | **X** | | **X** | | **X** | | **X** | | **X** |
| Breast milk PUFA | |  | |  | |  | | **X** | | **X** | |  | |  | |  | |  |
| *Home environment* | |  | |  | |  | |  | |  | | **X** | |  | | **X** | | *X* |
| Maternal Intelligence | | **X** | |  | |  | |  | |  | |  | |  | |  | |  |

weekly basis to distribute supplements and monitor compliance. *These workers will have a minimum qualification of having completed high school and will be trained to provide referrals for health care if required.* We expect about 100 - 400 women in the study per month and each worker will cover approximately 40-50 women. A team of 4*-5* trained nurses will obtain all baseline measurements (anthropometry, sociodemographic characteristics and obstetric history, diet, blood samples) at recruitment. They will also collect similar data for infants as well as blood and breast milk samples from mothers and *assist in measuring visual and auditory evoked potentials* and monitoring the birth outcomes to be collected as part of routine hospital procedures. All infant measurements will be done at the study headquarters in the IMSS General Hospital I. Mothers will be scheduled to bring their infants at 1, 3, 6, 9, 12 and 18 months, at which time growth and neurodevelopment outcomes will be measured. The total contact time per mother-infant pair is expected to range from 1-2 hours per visit of which the contact time for the infant ranges from 30 minutes at 9 months to 90 and 110 minutes at 12 and 18 months of age, respectively. The date of each assessment will be calculated based on birth date, plus or minus 7 days. A team of 3 trained psychologists will conduct all neurodevelopment and psychological measurements at the study headquarters. These include maternal intelligence at recruitment and the infant neurodevelopment outcomes, namely Neonatal Behavioural Assessment Scale (NBAS), Fagan and Bayley tests of motor and mental development (see section D. 2.2.1. for details). Approximately 4-5 infants will be scheduled daily (6 days a week) to be measured by the study nurse and psychologist. *Finally, two trained social workers will collect data on home environment during home visits at 6 and 12 months of age and at the study headquarters at 18 month of age.* Other study personnel will include an assistant who will coordinate all appointments and follow-up for data collection. Data entry operators (2) and lab assistants (1) will assist in data entry and cleaning and handling and analysis of biological samples at INSP.

**D.2.2. Measurement of Key Outcomes**

#### D.2.2.1. Infant Neurodevelopment *(revised)*

#### Neurodevelopment will be assessed at birth, and at 1, 6, 12 and 18 months of age for all infants born to women who participated in the intervention trial during pregnancy.

***a. Apgar and Neonatal Behavioral Assessment Scale (NBAS):*** The infant’s condition shortly after delivery will be evaluated using the **Apgar Score**[[193]](#endnote-193), a test routinely conducted at 1, 5 and 10 minutes post delivery to assess the infant’s status in five areas: heart rate, respiration, tone, reflex, and color. Scores for the individual areas range from 0 to 2, with a total number of 10 points possible. Total scores of 7 and above indicate the child is in good condition and at low risk for needing immediate medical attention. Infants whose 5-minute score remains low (0-3) are at risk for lasting problems, including neurological damage such as cerebral palsy or mental retardation[[194]](#endnote-194). The **Neonatal Behavioral Assessment Scale** (NBAS)[[195]](#endnote-195) will be administered to infants 48 hours after birth and at 1 month of age. The NBAS yields information on both the infant’s behavior (responses to certain stimuli -28 items), and reflexes (18 items), with supplementary information provided on qualities such as alertness, irritability, and endurance. The NBAS takes approximately 20-30 minutes to administer and will be done by trained examiners with some background in theories of child development and/or or experience with babies and neonates.

***b. Global mental and motor development***: Mental and motor development will be assessed at 6, 12 and 18 months of age using **the Bayley Scales of Infant Development-II** (BSID-II)Error: Reference source not found, which have been used in other studies of the

relationship between infant LCPUFA status and behavioral outcomes in humans[[196]](#endnote-196). The BSID consist of 3 scales: the Mental Scale (MDI), the Psychomotor Scale (PDI), and the Behavior Rating Scale *(see Appendix III for English and Spanish versions used in previous studies*). The MDI evaluates memory, habituation, problem solving, early number concepts, generalization, classification, vocalizations, and language. The PDI evaluates control of the gross muscle groups including movements associated with rolling, crawling and creeping, sitting, standing, walking, running and jumping. The PDI also tests fine motor manipulations involved in prehension, adaptive use of writing implements, and imitation of hand movements. The Behavioral Rating Scale assesses the child's behavior during the testing situation, which facilitates interpretation of the Mental and Motor Scales. The three scales are considered complementary, and each makes a unique contribution to the evaluation of the child. Each evaluation begins by administrating the recommended starting item from the age-appropriate item set. The infant’s raw summary scores on the Mental and Motor Scales are computed by adding the total number of items for which the child receives credit on each scale and all items below the basal item. The raw scores are then translated into Mental and Psychomotor Development Indices that are standardized to a mean of 100 ± 15. The average reliability is expected to range between 0.84 – 0.92 for the different components in children aged 6-18 mos. Interscorer agreement is 0.96 and 0.75 for the mental and motor scales, respectivelyError: Reference source not found. The BSID also provides guidelines for classification of these standardized scores into the categories of “Accelerated Performance” (115+), “Within Normal Limits” (85-114), “Mildly Delayed Performance” (70-84), and “Significantly Delayed Performance” (69 and below). Previous work has shown that the total duration of these tests increases with age and ranges between 25- 40 minutes per contact.

***c. Visual and Brainstem Auditory Evoked potentials, visual recognition memory and visual attention****: Since BSID assesses global development, more specific neurophysiological measures are required: visual evoked potentials (VEP), brainstem auditory evoked potentials (BAEP), visual recognition memory and visual attention.*

***VEP and BAEP*** *will be assessed in infants at 1 month of age at the hospital by a trained nurse and neurologist. VEP will be elicited by a light-emiting diode (LED) photostimulator fitted in goggles, without dark adaptation; pupils will not be dilated; the stimuli will be presented at 0.5/s, delivering an output intensity of 1500 cd/m^2^. VEPs will be recorded from an active electrode at the inion (Oz) referenced to a mid-frontal electrode (Fz). At least 2 averages of 64 trials each will be collected**[[197]](#endnote-197). For BAEP, surface gold-cup electrodes, 4mm in diameter, attached with paste and tape will be used in both ears for stimulation and for recording. Electrode impedances will be below 5 kΩ. Electrical activity will be evoked and recorded on a Nicolet Spirit. The click stimulation will be presented monoaurally at 70 dB hearing level and repeated 3000 times. The auditory stimulus is constant polarity rarefaction click delivered at 10/s via pediatric headphones. BAEPs will be recorded between the vertex (Cz) and the mastoid ipsilateral to the stimulated ear (A1 or A2)Error: Reference source not found. The total duration of these assessments will be 60 minutes and will not cause any discomfort to the infants.*

*Visual recognition memory (novelty preference) and visual attention (look duration)* *will be assessed at 6 and 12 months using* ***the Fagan Test of Infant Intelligence (FTII)****[[198]](#endnote-198). The FTII is a standardized, paired-comparison test, was developed to identify infants at risk for cognitive deficits[[199]](#endnote-199) and has been shown to be predictive of later performance on standardized intelligence tests[[200]](#endnote-200),[[201]](#endnote-201),[[202]](#endnote-202). In this test, the infant is seated on his or her mother's lap in front of a stage and is presented with pictures of faces. The complete test uses a series of 10 familiarizations, each of which is followed by a paired-comparison trial in which the novel stimulus is presented first on one side and then on the other. Tasks include “familiarization”, “duration of each look”, and “visual attention to a novel stimuli“. Preference for the novel picture, expressed as `percentage fixation preference', shows that the two pictures are discriminable, and also indicates that the infant can recognize one of the pictures as familiar. The final value is called the `novelty score'. This score is the length of fixation time during the test phase devoted to the novel picture divided by the total fixation time to both the novel and familiar picture, multiplied by 100. The required interval for familiarization decreases with increasing age, as does the time allowed for each paired-comparison test.*

#### D.2.2.2. Maternal and Child Anthropometry (*revised)*: Maternal anthropometry will be obtained at recruitment and will include height, weight, mid-arm circumference, and triceps and subscapular skinfold thickness using standard procedures**[[203]](#endnote-203)** by trained workers. Details of maternal weight gain during pregnancy will be obtained from medical records. Weights will be recorded to the nearest 0.1 kg and heights to the nearest 0.5 cm. Length, weight and head circumference of the infants will be measured at birth and at 1, 3, 6, 9, 12 and 18 months of age using standard procedures. Data collectors will be trained in anthropometric techniques, and regular standardizations will be carried out. Measurement of accuracy and precision for all anthropometric measurements will be done using methods recommended by Lohman et al (1988)Error: Reference source not found. The scale will be calibrated twice daily with a known reference weight. All data collection will be done at the study headquarters and will be obtained in duplicate by the same data collector. Children will be weighed using a pediatric weighing scale with a precision of 10 g. Infants will be weighed without clothing at birth and with a minimum of clothing (e.g., undershirt, underpants or dry diaper) for subsequent measurements. The weight of clothing will be adjusted for using a second set of clothing. Recumbent length will be measured using a baby-board (UNICEF, Copenhagen, Denmark). Measurements will be read to the nearest 0.1 cm. Weight-for-age and length-for-age z scores will be calculated using the National Center for Health Statistics reference data**[[204]](#endnote-204)**. Head circumference will be measured at the largest occipitofrontal circumference to the nearest 0.1 cm with a non-stretchable tape.

D.2.2.3. Gestational Age and Birth Outcomes *(revised):* Details of birth outcomes (type of delivery, sex, livebirth, etc) will be obtained from hospital records and will be verified by interview in a 10% sub-sample. Gestational age will be determined at birth using Ballard’s method[[205]](#endnote-205).

D.2.2.4. Long Chain Polyunsaturated Fatty Acid Status (Maternal and Infant): *(revised)*

*Fatty acid composition of plasma phospholipids and RBC membrane phospholipids are appropriate markers of recent intake because they respond rapidly[[206]](#endnote-206). Since the distribution of fatty acids in the phospholipid fraction of plasma is closely related to their distribution in the phospholipids of the RBC membrane, only plasma phospholipid fatty acids (ALA, LA, AA, EPA, and DHA) will be measured in a random 30% subsample of pregnant women and their infants. Maternal blood samples (7 ml) will be obtained by venipuncture at recruitment and at delivery. Neonatal blood samples will be obtained from the umbilical cord vein immediately after delivery. A 5 ml venous blood sample will be obtained at 3, 12 and 18 months of age from infants. All samples will be collected by trained nurses and placed into tubes containing disodium ethylene diamine tetraacetic acid (EDTA). Plasma and RBCs will be separated by centrifugation at 800 g for 10 min at room temperature. Plasma will be frozen in a nitrogen environment and stored at -70° C for later analysis at INSP laboratories. The maternal and infants' plasma total lipids will be extracted using the procedure described by Folch et al[[207]](#endnote-207) and individual and total plasma phospholipid fatty acid methyl esters will be identified using gas-liquid chromatography and expressed both as relative weight (% by wt) and in absolute concentration (mg/L).* *Details of lipid extraction, fatty acids methylation and analysis are provided in Appendix V. Details of quality control are described in section D.2.4.*

D.2.2.5. Breast milk PUFA: *(revised)* Breast-milk samples will be collected at 1 and 3 mo post-partum in the same subsample of women who provide venous blood samples. The samples will be taken from a morning feed but not the first one, between 8 and 12 o'clock at the study headquarters. Infants will be allowed to suckle the nipple for a few minutes, and then a breast milk sample (10 ml) will be taken and the feeding continued[[208]](#endnote-208). The samples will be placed in 14 ml glass test tubes, labeled and sealed, and immediately transferred to the freezer at -70°C until analysis. Before storage, the samples will be sonicated and EDTA and butylated hydroxytoluene will be added to a final concentration of 1.85 mg/mL and 75 μg/mL, respectively. Although the amount of fat may change within and between feeds, the proportion of fatty acid remains relatively constant[[209]](#endnote-209),[[210]](#endnote-210). Since breast milk PUFA will be expressed as a % of total fatty acids, complete breast expressions are therefore not required[[211]](#endnote-211). *The fatty acid composition of breastmilk will be analyzed by gas chromatography using standard methods[[212]](#endnote-212),[[213]](#endnote-213) (see Appendix V for details).*

#### D.2.3. Measurement of Potential Confounders

Randomization is expected to ensure comparability of the groups for most maternal and infant characteristics that may influence the key outcomes. However, this needs to be assessed, and controlled for appropriately especially if there are differences. Data will therefore be collected on the following maternal and infant characteristics.

D.2.3.1. Sociodemographic Characteristics and Obstetric History: Data on maternal sociodemographic characteristics and obstetric history will be collected using questionnaires that have been used in previous studies by the study investigators. The instrument used by Dr. Parra (see Appendix *III*) has been validated for use in pregnant women from low-medium socioeconomic status at the National Institute of Perinatology in Mexico. It contains 55 questions that can be administered by a nurse or social worker and takes approximately 20-25 minutes. The first section includes questions regarding marital status, place of birth, years of schooling, occupation, household income and composition. The second section includes questions about reproductive history, like number of pregnancies, number of abortions, causes, number of live children, number of death children and causes, previous and current pregnancy complications like: hypertension, convulsions, bleeding, etc. The third section contains questions about nervous sicknesses, depression, psychiatric disorders, and addictions like smoking, drug and alcohol use.

D.2.3.2. Maternal pre-pregnant nutritional status: Maternal prepregnant nutritional status especially fat stores may influence the outcomes of interest. Prepregnant weight (wt) and height (ht) will be determined from hospital records and body mass index (BMI) will be calculated (BMI = wt in kg /(ht in m)^2^) Since many women access prenatal care early, the measurements at the first prenatal visit will be used.

D.2.3.3. Maternal Diet *(revised): A 24-hour recall survey protocol that has been used and validated in previous studies in Guatemala and Mexico[[214]](#endnote-214) (see Appendix III) will be used to estimate and compare mean intakes of macronutrients (energy (kcal), fat (g, % of total energy), protein (g, % of total energy), carbohydrate (g, % of total energy), total monounsaturated acids (g, % of total energy), total PUFA (g, % of total energy), total saturated fatty acids (g, % of total energy), total EFA (g, % of total energy), total n-3 PUFA (g, % of total energy), and individual fatty acids(ALA (g), LA (g), AA (mg), EPA (mg), DPA (mg), and DHA (mg)) in the two groups. Patterns of consumption of DHA rich foods will also be compared. A single 24-hour dietary recall will be administered at recruitment (20-24 wks gestation) and at 3 and 6 months postpartum by trained nurses at the study headquarters. The period of recall will be for the day before the interview beginning at 6 am and ending at 6 am on the day of the interview. The administration of this instrument takes approximately 30 minutes. The multipass method will be followed. The 3 passes will include (1) an overview of the days activities and eating occasions; (2) a detailed inventory of foods eaten at each occasion; and (3) a review and set of probes to ascertain any additional food items that may have been omitted. Local cooking and serving utensils and a kitchen scale will be used to assist in the estimation of portion size and details of recipes will be obtained. The amounts of food ingested will be recorded in grams. Data entry and conversions from food items to nutrient values will be done using a compilation of food composition tables for Latin American foods and extensively tested computer programs developed at INSP. The food composition tables include values obtained from INSP, INCAP and the United States Department of Agriculture[[215]](#endnote-215).*

D.2.3.4. Maternal Intelligence: Maternal cognitive functioning can influence infant development, as the mother is typically the primary caregiver during the early years. We will therefore assess her cognitive functioning at recruitment using Raven’s Progressive Matrices, a nonverbal test of intellectual functioning which requires the participant to figure out complementary abstract patterns[[216]](#endnote-216). The Raven’s test has been shown to be a useful measure of generalized intelligence, especially in low literacy societies and has been used by the investigators in previous studies in Guatemala and Mexico. The test measures the ability to form comparisons, reason by analogy, and to organize spatial perceptions into systematically related wholes. The Standard Progressive Matrices form, which contains 60 items presented in five sets (12 items per set), will be used. Raw scores will be converted into percentile ranks. The test takes between 15 and 30 minutes and will be administered by trained psychologists[[217]](#endnote-217).

D.2 3.5. Home Environment: Aspects of the home environment have been shown to be strongly associated with cognitive and language development[[218]](#endnote-218),[[219]](#endnote-219). In order to control for these potential influences when examining the effects of DHA supplementation on child growth and development, the HOME Inventory, a measure of the home environment that is widely used, will be employed[[220]](#endnote-220). The HOME Inventory assesses the quality and quantity of social, emotional, and cognitive support available to a child in the home environment, and is composed of six subscales: parental responsivity, acceptance of child, organization of the environment, provision of appropriate materials, parental involvement, and variety of stimulation. Several reviews have suggested that this inventory is a reliable and valid measure of the home environment and that it is related in expected ways to several health and development measures, as well as to many ecological factors[[221]](#endnote-221),[[222]](#endnote-222). This inventory will be completed in the home by trained social workers at 6 and 12 months of age and will take approximately one hour to complete. *A reduced version of the scale will be administered by the social workers during the hospital visit at 18 months of age primarily to capture any major changes in the home environment since the last visit and will take ~ 20-30 minutes.*

D.2.3.6. Infant Diet *(revised)* : Since breastfeeding is associated with neurodevelopmental outcomes in infants[[223]](#endnote-223) and the duration of breastfeeding cannot be controlled by study design, the duration of breastfeeding will be controlled in the data analysis. *Feeding practices and dietary intakes will be assessed using a structured 24 hour recall survey (Appendix III) by trained nurses at the time the infant visits the hospital.* Exclusive breast-feeding will be defined in accordance with the WHO[[224]](#endnote-224) definition (the infant receives breast milk only with no other liquids or solids). Predominant breast-feeding will be defined as receiving breast milk plus fluids (e.g., water, juices) and less than 2 fluid ounces of any commercial infant formula per day. *Administration of the 24-hour recall and calculation of energy and nutrient intakes will follow protocols similar to that described under maternal diet.*

***D.2.4. Data Quality Control***

*In this section we describe several procedures to ensure data quality and estimate the extent of measurement error for the various outcome measurements and confounding factors. Key to ensuring high quality data will be extensive training of the team of interviewers, and supervision during data collection and data entry. The 3 area supervisors (see Figure 4) will verify all forms for accuracy and completeness on a routine basis before data entry at INSP, and incorrect or incomplete entries will be corrected as far as possible during data collection. Further, to avoid systematic biases, all interviewers within each area of measurement will be randomly assigned to subjects using computer based algorithms as in our previous studies. Estimates of validity and reliability will be obtained for selected measurements by collecting either duplicate measurements by supervisors blinded to the interviewers’ observations and/or additional data on study subjects or comparable non-participants as described below.*

1. *A supervisor will verify measurements in a 10% random subsample for a) maternal and child* ***anthropometry****; b) birth outcomes; c) sociodemographic characteristics and obstetric history status; and d) infant feeding practices. These measurements will be done within 1-2 days of the original measurement. Data collectors will not know in advance which subsample is being verified.*

*2) The accuracy of estimates of* ***gestational age*** *obtained at birth will be validated by comparing them to estimates obtained from ultrasound examinations to be conducted during the routine prenatal visit at approximately 32 weeks gestation in a 10% random subsample.*

*3) For* ***psychological assessments*** *(infant neurodevelopment, maternal intelligence and home environment) and dietary intakes (24 hour recalls for mothers and infants), the supervisors will verify data quality as follows:*

1. *A 10% random sub-sample of subjects will be interviewed independently at the different time points by the supervisor. Data collectors will not be aware of the sub-sample being verified.*
2. *A different 10% subsample of interviews will be directly supervised during the interviews.*

*4) In the case of* ***biochemical estimations***

1. *Duplicate aliquots for a 10% subsample of blood and breast milk samples will be analyzed at Nutrek, Inc Essential Fatty Acid laboratory, Brookline, MA, USA (see Appendix V.)*
2. *All samples will be carefully labeled (type of specimen, identification number and date of collection) using indelible markers and frozen on the same day of collection at INSP after separation and preparation of aliquots. Dry ice will be used for transportation and the temperatures of storage freezers will be checked daily.*
3. *All lab personnel will be blinded to the supplementation codes and all estimations will be done by the same chemist highly trained and experienced in gas chromatography. High quality external standards will be procured and the maximum allowable CV will be 18% for each fatty acid.*

*Measurement error will also be estimated for the key outcome measures of infant development in an independent sample of age-appropriate mother-child pairs (n=30) living in the same general area of the study population, who are not participating in the main study. All the examiners and area supervisors will obtain replicate, blinded measurements within the same week, with the order in which they conduct the interviewers to be assigned at random. Standard methods will be used to calculate the inter-rater reliability coefficient[[225]](#endnote-225) to estimate the degree of error. Using methods originally developed in nutritional epidemiology to account for within-subject variability and measurement error in dietary intakes[[226]](#endnote-226) we will correct effect estimates for the observed measurement error[[227]](#endnote-227).*

**D.3. Data entry and management *(revised)***

All data forms will be checked for accuracy and completeness by the study supervisors and by the data entry operators at INSP. All data forms will be sent weekly to the data entry center at INSP and 100% double-entered into the project’s computer files using FoxPro^TM^ *.* Incorrect entries and missing data will be checked and verified on a systematic basis using range checks for values beyond permissible values and missing values. *All datafiles will be routinely backed up using read-writable CDs and copies will be kept at INSP and Emory University.*

**D.4. Data Analysis**  *(no changes*)

**D.4.1. Overview of Statistical Approach**:

Generally, we will analyze outcomes from birth to 12 months of age among all children who reach that time point. In further analyses to be conducted in the subsample of children who are followed up until 18 months of age, missing interim endpoints will be imputed using multiple imputation procedures as described by Little and Rubin[[228]](#endnote-228). Effectiveness of randomization will be examined by comparing the two groups on key baseline characteristics. In the case of significant differences, group differences in outcomes will be examined with and without adjusting for these factors. Loss to follow-up is expected to be 20% to 18 months. We will assess selection bias by comparing baseline characteristics of subjects with and without the outcome measure of interest and control for any observed differences in the analyses.

All statistical analysis will be done using Statistical Analysis Software (SAS)[[229]](#endnote-229). A probability of p<0.05 will be considered statistically significant for all hypothesis testing. Assumptions of normality will be assessed using plots and tests of normality. Non-normal variables will be transformed or categorized as required. Descriptive data analysis will include examining the distributions of neurodevelopment, dietary intakes, anthropometric and PUFA status. Means, standard deviations, quartiles and median levels will be reported. Individual analytical hypotheses will be tested using the following general statistical techniques:

D.4.1.1. Outcomes measured at one time point: These are analyses which model outcomes collected at one point in time as a function of variables collected either cross-sectionally or longitudinally. For example, cognitive development at 18 months may be modeled as function of prenatal and postnatal growth, diet, and social confounding factors. These analyses can take several forms.

i. Analysis of Covariance will be used for comparison of means of two or more categories (e.g. supplement groups) adjusting for covariates, which may be either continuous or categorical.

ii. General linear models based on least squares fit will be used when the dependent outcomes are continuous variables such as indicators of child growth and development and nutrient intakes. Independent variables can include continuous, categorical and dummy (0/1) variables. Two approaches, Ordinary Least Squares (OLS) regression and Two Stage Least Squares (2SLS) will be used, depending on the issues addressed.

iii. Logistic regression may be used if the response variable is dichotomous (e.g. stunting defined as ht/age Z score <= -2 ). Adjusted odds ratios will be calculated to estimate risk for the independent variable of interest namely supplement use while controlling for other confounding variables.

D.4.1.2. Outcomes measured on repeated occasions: Some of the data are obtained from repeated measurements on individuals over time (e.g. length measurements at birth, 1, 3, 6, 9, 12, and 18 months of age). Such longitudinal data are very useful for describing dynamic processes which would be poorly captured by a single outcome or "point prevalence" assessment. Independent variables can also be time varying (e.g. feeding practices by time period: 0-3, 3-6, 6-9, 9-12 and 12-18 months) or fixed (e.g. household SES). Examples of the variables we will be modeling longitudinally include:

- **Child outcomes:** birth size, growth, motor and mental development
- **Determinants of interest:** Supplement group
- **Controlling for confounding variables:** SES, maternal BMI, intelligence, home environment.

The analytic complexities that are introduced by this approach can be addressed using methods that have been developed recently for handling longitudinal/repeated measures data, namely Generalized Estimating Equations (GEE) approach and mixed models. The GEE approach is useful for analyzing either discrete or continuous longitudinal data and accounts for the correlation among repeated measures in the calculation of both parameter and variance estimates[[230]](#endnote-230),[[231]](#endnote-231). Additionally, it is able to accommodate time‑dependent as well as time‑independent covariates. For analyses in which the response variable is continuous and the assumption of multivariate normality is met, an alternate approach is to do mixed model analyses, using, for example, the SAS procedure PROC MIXED[[232]](#endnote-232). This procedure allows the inclusion of both fixed and random effects and, in repeated-measures situations, is more flexible and widely-applicable than standard univariate or multivariate approaches.

D.4.1.3. Structural Equation Modeling (SEM): Structural equation modeling (also known as path analysis and simultaneous equation systems) is an advanced multivariate technique widely used for testing causal models in behavioral and social research[[233]](#endnote-233),[[234]](#endnote-234). This approach will be used to test the significance of the various pathways proposed in the conceptual framework (Figure 1) accounting for the covariance within the different constructs. SEM is also useful to test bi-directional causal relationships (eg: caregiver interaction and motor development) and longitudinal data and can incorporate latent variables. SEM is particularly suited to handle issues of endogeneity inherent in the models to be developed. In order to obtain unbiased estimates, one develops multiple equations to describe the mechanisms of the system, one for each endogenous variable. The simplest and most commonly used technique for the estimation of systems of linear simultaneous equations is the class of instrumental variables estimators [[235]](#endnote-235),[[236]](#endnote-236) of which two-stage least squares (2SLS) is the most important special case. We will use this approach extensively. Recent versions of the appropriate computer packages (eg: AMOS, EQS, PROC CALIS in SAS) for testing complex causal models by SEM will be used[[237]](#endnote-237)*.*

**D.4.2. Analytical Plan:**

The principal outcome variables of interest will be birth outcomes (birth weight, length, maternal and newborn PUFA, neurodevelopment), breast milk PUFA levels, and child growth (weight, length, head circumference) and skinfold thickness) and development (cognitive and motor) at 18 months of age. In addition, weight and length gain from birth to 18 months of age, neurodevelopment at 1, 6 and 12 months, cognitive and motor development at 6 and 12 months and infant PUFA status at 1 and 3 months of age will also be examined. Preliminary descriptive analyses will be done to examine the distribution of the data and check for outliers, following which unadjusted comparisons of mean differences in length and weight and biochemical values will be made using Student's t test. The intervention groups will be compared on several baseline variables to ensure that randomization worked. Chi-square tests will be used to compare categorical variables and Student’s t test will be used to compare normally distributed continuous data. Following this, adjusted comparisons (using co-variance analysis)[[238]](#endnote-238) will be made if controlling for potential confounders is required using linear multiple regression for continuous variables and logistic regression for categorical variables[[239]](#endnote-239). In all cases, results will be presented before and after adjustment for confounding and testing of interactions.

Examples of some of the models for various hypotheses are presented below. For specific hypotheses 2a, using birth weight as the outcome of interest and general linear models regression analysis, we can test the following model:

***2a. Birth weight = Constant + β_1_(Supplement)+ β_2_ (Other confounding)* *+ β_3_  (Supplement * other confounding)***

Similar models can be used to test specific hypothesis 3a and 3b using attained weight and length and cognitive and motor development at 18 months of age as the outcomes of interest. For example,

***3a. Length (18 mo) = Constant + β _1_ (Supplement)+ β_2_ (Other confounding)* *+ β_3_  (Supplement * other confounding)***

***3b. Mental development ( 18 mo) = Constant + β_1_ (Supplement)+ β _2_ (Other confounding)* *+***

***β_3_  (Supplement * other confounding)***

Similar models can be specified to test the hypotheses stated under specific aims 1 & 4, for example that breast milk PUFA levels and infant PUFA status at 1 month of age will be higher in the intervention group compared to ‘control’ group. Mean values of breast milk and blood levels of DHA will be used as the dependent variables. Since some outcomes will also be defined as dichotomous outcomes, some of the hypotheses will also be tested using logistic regression. For example, if the dependent variable for specific hypothesis 3a is prevalence of stunting at 18 months of age that is defined using cut-offs for height/age Z score (HAZ), the model will be as follows:

***Pr {HAZ <= -2 at 18 mo} = Constant + β_1_ (Supplement)+ β_2_ (Other confounding)* *+ β_3_ (Supplement * other confounding)***

The various pathways indicated in the conceptual framework will be tested using techniques described in SEM. For example, 2SLS can be used to model birth weight as a function of supplement group controlling for other confounding, and in turn these estimates will be used to predict attained size at 18 months of age. Similar models will be developed for indicators of neurodevelopment and examine the role of caregiver interaction.

**E. HUMAN SUBJECTS CONCERNS *(revised)***

**E.1. Subject population**

The study participants are pregnant and lactating women (18-35 y of age) and their infants from birth to 18 mo of age who currently live in Cuernavaca, Morelos State, Mexico. The population is of mixed Spanish-indigenous origin (Ladinos) and speaks Spanish.

## E.2. Sources of research materials

Data will be collected from study individuals by questionnaires, clinical examinations and medical records. The types of data to be collected are described in section D. Some instruments are included in *Appendix III*. All data except biological samples will be collected from all women and infants. Venous blood and breast milk samples will be obtained from a random sub-sample of mothers and their infants for determination of PUFA levels. All data collection uses well-established techniques and includes interviews and clinical assessment. All methods are non-invasive except that, the collection of biological samples is minimally invasive. Data collection is at recruitment, delivery and several time points during infancy (1, 3, 6, 9, 12, and 18 months of age) as shown in Table 5 in section D. Most of the data collection will be conducted at the study headquarters at the IMSS General Hospital I except that the assessment of home environment will be done at the woman’s home.

## E.3. Plans for recruitment

Pregnant women will be identified through hospital records maintained at IMSS General Hospital I. All eligible women will be approached by the study physician who will explain the study and seek their participation.

**E.4.Potential risks**

We judge the potential risks from data collection to be minimal. No invasive procedures are under consideration except for collection of venous blood and breast milk samples; these procedures are minimally invasive and voluntary. Blood samples will be obtained from a subsample of women and infants following routine practices. Appropriate precautions will be taken to avoid inflicting harm or risk to the well being of the subjects. Subjects detected with severe anemia or other life threatening conditions will be followed up to receive appropriate treatment.

The intervention is itself considered safe and the possibility of adverse effects has been minimized by the use of i) stringent inclusion and exclusion criteria and ii) a safe form (algal) and dosage (200 mg) of DHA. Total DHA intakes will remain within a safe range (< 1 g/d) even when combined with dietary sources. Subjects will be free to withdraw from the study at any time. Any potential adverse effects will be monitored and reported by the study investigators immediately to the Data Safety Monitoring Committee as well as to the Human Subjects Committee at INSP and Emory for appropriate action. Potential risks to participants include mild side effects such as gastric discomfort and the identification of new health problems that may be upsetting to participants. Any such findings will be explained to the participant and appropriate follow-up will be advised. Even though the potential for injury to research subjects due to the risks of the proposed procedures is thus judged to be minimal, all reasonable efforts will be made to minimize these risks through the exclusive use of properly trained and educated research personnel. Individuals who are injured by study procedures will be informed of their rights to and be offered treatment of the injury as part of the informed consent procedure and the prevailing local health policy. Detailed procedures for the notification of any abnormal findings of clinical relevance as well as non-emergency and emergency referrals will be developed and implemented. Being in a clinical setting, all subjects will have access to readily available high quality care at all times. A hot-line will exist for the mothers to refer any adverse effects events at the hospital*.*

## E.5 Procedures for protection of human subjects

Many of the protocols to obtain information through questionnaires proposed in the current study have been used in previous studies without evidence of adverse effects. These protocols and instruments have been approved periodically by the appropriate Human Subject Review Boards. The study protocol will be submitted for approval to the Human Investigations Committees at Emory University and National Institute of Public Health in Mexico.

Informed consent will be obtained from all subjects at recruitment (20-24 weeks gestation) and again from the parent/caretaker at the time of birth for the infant. The details of the study procedures with potential risks and benefits will be explained. Subjects will be free to withdraw from the study at any time without prejudice or coercion. A consent form will be developed and will be submitted for approval to the Human Investigations Committees of both Emory University and INSP prior to any data collection. The consent form will closely follow the format we have used in our previous studies in the population of this region. It will describe the study, the respondent burden, the potential risks and benefits, and provide names and contact details of individuals in Mexico who can be contacted for additional details. Interviewers will read the form to the respondent, offer the opportunity to have any questions answered, then hand it to the respondent, and obtain a signature. If the respondent is illiterate, a friend can witness the consent process. Verbal consent will be obtained and documented before administration of each module. A copy of the consent form will be provided to the respondent to keep.

## E.6. Procedures for ensuring confidentiality

The study will obtain a Certificate of Confidentiality, issued by the National Institutes of Health, to cover a) all new data that will be collected by the study and b) data that will be obtained from medical records (weight gain during pregnancy and birth outcomes). The purpose of a Certificate of Confidentiality is to prevent anyone other than study investigators from gaining legal access to data collected for research purposes. Study subjects can thus be assured that information revealed by them during an interview or collected as part of the examination will be kept confidential and cannot be subpoenaed or obtained by legal means except with their permission.

The study will employ standard methods for protecting the confidentiality of research materials through the use of coded identification numbers on all materials, password-protected computer data files, and locked file cabinets for storing hard copies of interview and other study materials. All interviewers will be trained in procedures to minimize the potential for breaches of confidentiality, including but not limited to ensuring that all files are closed, that interviews are conducted in private settings, and that no conversations about individual study participants occur in public settings.

Names and other easily recognizable identifiers will be removed from all questionnaires prior to data entry and will not be included in any electronic databases. Numeric study identifiers are included so that data from the several instruments may be linked, however, these are not meaningful to casual observers without access to the original study logs. All data files will be maintained under password protection at all times, whether at IMSS, INSP or at Emory University.

## E.7. Anticipated benefits

We expect that at least half the study participants will benefit, namely those who receive DHA supplements. However they will not know this until the completion of the trial and benefit cannot be guaranteed. Possible benefits to research participants include a better understanding of factors that may affect their health. Special efforts will be made to ensure that all study subjects have access to improved health care, which will be facilitated by routine screening by the study nurses and timely referral to the health facilities.

## E.8. Results with possible clinical implications

Although expected to be infrequent, additional benefits may come from the detection of previously unknown health problems that, with treatment, will result in better future health for participants.

## E.9. Women and minority inclusion in clinical research

Women will be well represented as the study subjects are pregnant women and their infants. We expect that half the children to be born and studied will be female. The population to be studied is of Ladino (mixed Spanish-indigenous) ancestry.

## E.10. Inclusion of children

Children are the focus of this study; child development is the primary outcome.

## E.11. Data and safety monitoring plan (revised)

# A Data Safety Monitoring Committee (DSMC) will be formed with experts well qualified in clinical trials among pregnant women *and infants* who are not affiliated with the study team institutions. Letters of support from Dr. Robert Goldenberg, Professor of Obstetrics and Gynecology, School of Medicine, University of Alabama, Birmingham, AL, Dr. Molly Cogswell, senior epidemiologist, Maternal and Child Nutrition Branch, Division of Nutrition and Physical Activity at the Centers for Disease Control and Prevention, Atlanta, GA and Dr. Esther Casanueva, National Institute of Perinatology, Mexico, who have all agreed to serve on the committee have been included in Appendix I. *The Data Safety Monitoring Committee will monitor the study for safety and ensure that human subjects protection is assured during the entire duration of data collection. In particular, the DSMC will pay close attention to any adverse outcomes in a) women during pregnancy and delivery, especially increased bleeding; and b) infants, especially those who are born to women recruited early in the study. The DSMC will have the power to stop the study if required. The DSMC will have frequent (monthly) interactions with the study team and will receive immediate communication of any adverse outcomes via email/fax/telephone. The committee will have access to unblinded data upon request and will have the power to call for meetings with the study investigators (either by telephone conference or in person) and close the study if required at any time.*

**F. VERTEBRATE (*no changes)***

None

INSP (*Appendix IV).*

**I. CONSULTANTS**

Dr. Ricardo Uauy and Dr. Maria Makrides have agreed to serve as consultants for this project and will assist in the implementation of the trial, data analysis and interpretation of findings. Letters of support have been included in Appendix I.

1. Based on stunting and underweight, defined as values <- 2. S.D.of the NCHS median for height/age and weight/age, respectively. [↑](#footnote-ref-2)
2. **G. LITERATURE CITED *(updated)***

   ? ACC/SCN. Fourth Report on the World Nutrition Situation. ACC/SCN, WHO, in Geneva in collaboration with IFPRI, Washington D.C., USA, 2000. [↑](#endnote-ref-2)
3. Ashworth A. Effects of intrauterine growth retardation on mortality and morbidity in infants and young children. Eur J of Clin Nutr. 52:S1, S34-42, 1998. [↑](#endnote-ref-3)
4. Hack M. Effects of intrauterine growth retardation on mental performance and behavior, outcomes during adolescence and adulthood. Eur J of Clin Nutr 52:S1, S67-S71, 1998. [↑](#endnote-ref-4)
5. De Onis M, Monteiro C, Akre J, Glugston G. The worldwide magnitude of protein-energy malnutrition: an overview from the WHO Global Database on Child Growth, Bull WHO;71:703-712, 1993. [↑](#endnote-ref-5)
6. ?Rivera J, González-Cossío T, Flores M, Hernández M, Lezana MA, Sepúlveda J. Emaciación y déficit de talla en menores de cinco años en distintas regiones y estratos en México. Salud Púb Méx 37:95-107, 1995. [↑](#endnote-ref-6)
7. ? Rozsenzweig MR, Leiman AL, Breedlove SM. Biological Psychology. Sunderland, MA: Sinauer Associates, Inc. pp 102, 112-113, 1996. [↑](#endnote-ref-7)
8. Crawford MA. The role of essential fatty acids in neural development: implications for perinatal nutrition. Am J Clin Nutr, 57(suppl):703S-10S, 1993. [↑](#endnote-ref-8)
9. Young ME. Early childhood development: Investing in our children’s future. Elsevier, the Netherlands, 1997. [↑](#endnote-ref-9)
10. ?Rivera J, Ruel MT. The timing of growth retardation in rural Guatemalan Children with adequate birth weight. FASEB J;7(3):abs No. 1636, 1993. [↑](#endnote-ref-10)
11. ?Ruel M, Rivera J, Habicht JP. Length screens better than weight in stunted populations. J Nutr;125:1222-1228, 1995. [↑](#endnote-ref-11)
12. ?Huttly SRA, Victora CG, Barros FC, Teixeira AMB, Vaughan JP, The timing of nutritional status determination: implications for interventions and growth monitoring. Eur J Clin Nutr;45:85-96, 1991. [↑](#endnote-ref-12)
13. ?Waterlow JC. Observations on the natural history of stunting, In Linear growth retardation in less developed countries, JC Waterlow (ed), 1, Vevey: Nestle Nutrition/New York: Raven Press, 1988. [↑](#endnote-ref-13)
14. ?Martorell R, Schroeder DG, Rivera JA, Kaplowitz, HJ. Patterns of Linear Growth in rural Guatemalan Adolescents and children. J. Nutr; 125(4):1060S-1067S, 1995. [↑](#endnote-ref-14)
15. ?UNICEF. Strategy for improved nutrition of children and women in developing countries. UNICEF policy review. New York: UNICEF, 1990. [↑](#endnote-ref-15)
16. ?Martorell R, Rivera J, Kaplowitz H, Pollitt E. Long-term consequences of growth retardation during early childhood. In Human Growth: Basic and clinical aspects, Ed Hernandez M, Argente J, 1992. [↑](#endnote-ref-16)
17. Leon DA, Lithell H, Vagero D, McKeigue P, Koupilova I, Mohsen R, Berglund L, Lithell UB, McKeigue PM. Reduced fetal growth rate and increased risk of death from ischaemic heart disease: cohort study of 15,000 Swedish men and women born 1915-29. BMJ; 317:241-245, 1998. [↑](#endnote-ref-17)
18. Kramer, MS. Balanced protein/energy supplementation in pregnancy. Cochrane Pregnancy and Childbirth Group Cochrane Database of Systematic Reviews; (Issue 4) 2000. [↑](#endnote-ref-18)
19. Schroeder, DG. Malnutrition In “Nutrition and Health in Developing Countries” Ed, Semba RD, Bloem MW. Humana Press, 393-426, March 2001. [↑](#endnote-ref-19)
20. Ramakrishnan U, Huffman S. Multiple Micronutrient Malnutrition – What can be done? In Nutrition and Health in Developing Countries. Ed: Semba, R.D., Bloem, M. Totawa, NJ: Humana Press, 365-392, March 2001. [↑](#endnote-ref-20)
21. Wachs TD. Relation of mild to moderate malnutrition to human development: correlational studies. J Nutr 1995;125(8):2245S-2225S, 1995. [↑](#endnote-ref-21)
22. ?Martorell R. Undernutrition during pregnancy and early childhood: consequences for cognitive and behavioral development. In: Early child development: Investing in our children’s future. Ed. Young ME. Elsevier Science BV, Netherlands, 1997. [↑](#endnote-ref-22)
23. ? Brown JL, Pollitt E. Malnutrition, Poverty and Intellectual Development. Scientific American, Feb 1996. [↑](#endnote-ref-23)
24. Levitsky DA, Strupp BJ. Malnutrition and the Brain: changing concepts, changing concerns. J Nutr;125:2212S-2220S, 1995. [↑](#endnote-ref-24)
25. ?World Health Organization (WHO). A Critical Link: Interventions for physical growth and psychological development. A Review. Dept of Child and Adolescent Health and Development, WHO, Geneva, Switzerland, 1999. [↑](#endnote-ref-25)
26. ? Uauy R, Treen M, Hoffman DR. Essential Fatty Acid Metabolism and Requirements during Development. Seminars Perinatology 13:118-130, 1989. [↑](#endnote-ref-26)
27. Uauy, Hoffman DR. Essential fat requirements of preterm infants. Am J Clin Nutr 71:245S-50S, 2000. [↑](#endnote-ref-27)
28. Sastry P: Lipids of nervous tissue: Composition and metabolism, Prog Lipid Res 24:69–176, 1985. [↑](#endnote-ref-28)
29. Budowski P: n3-Fatty acids in health and disease. World Rev Nutr Diet 57:214–274, 1988. [↑](#endnote-ref-29)
30. Carroll KK. Upper limits of nutrients in infant formulas: polyunsaturated fatty acids and trans fatty acids. J Nutr 119:1810–3,1989. [↑](#endnote-ref-30)
31. Flieser SJ, Anderson RE. Chemistry and metabolism of lipids in the vertebrate retina. Prog Lipid Res; 22:79-131, 1983. [↑](#endnote-ref-31)
32. Weber PC, Fischer S, von Schacky C, Lorenz R, Strasser T. Dietary omega-3 polyunsaturated fatty acids and ecosanoid formation in man. In: Simopoulus AP, Kifer RR, Martin RE eds. Health effects of polyunsaturated fatty acids in sea foods. Orlando FL: Academic Press:49-60, 1986. [↑](#endnote-ref-32)
33. Dutta –Roy A. Transport mechanisms for long-chain polyunsaturated fatty acids in the human placenta Am J Clin Nutr;71(Suppl):315S-22S, 2000. [↑](#endnote-ref-33)
34. Innis SM. Essential fatty acids in growth and development. Prog Lipid Res;30:39-103, 1986. [↑](#endnote-ref-34)
35. Uauy R, Peirano P, Hoffman D, Mena P, Birch D, Birch E. Role of esential fatty acids in the function of the developing nervous system. Lipids; 31:S167-S176, 1996. [↑](#endnote-ref-35)
36. Stevens LJ, Zentall SS, Deck JL. Essential fatty acid metabolism in boys with attention-deficit hyperactivity disorder. Am J Clin Nutr;62:761-768, 1995. [↑](#endnote-ref-36)
37. Neuringer M. The relationship of fatty acid composition to funtion of the retina and visual system. In J. Dobbing (Ed): Lipids, learning, and the brain: Fats in infant formulas. Pp 134-165. Report of the 103rd Ross Conference on Pediatric Research. Columbus, Ohio: Ross laboratories. 1993. [↑](#endnote-ref-37)
38. Koletzko B, Schmidt E, Bremer HJ, Haugh M & Harzer G. Effects of dietary long-chain polyunsaturated fatty acids on the essential fatty acid status of premature infants. European J Pediatr;148:669-675, 1989. [↑](#endnote-ref-38)
39. Clandinin MT, Chappell JE, Leong S, Heim T, Swyer PR, Chance GW. Intrauterine fatty acid accretion rates in human brain: Implications for fatty acid requirements. Early Hum Dev 4:121-129, 1980. [↑](#endnote-ref-39)
40. Martinez M. Tissue levels of polyunsaturated fatty acids during early human development. J Pediatr;120:S129-S138, 1992. [↑](#endnote-ref-40)
41. Wells J. Infant and follow-on formulas: the next decade. British Nutrition Foundation. Bulletin 23; (Supplement 1);23-24, 1998. [↑](#endnote-ref-41)
42. Crawford MA, Hassam AG, Williams G. Essential Fatty Acids and fetal brain growth. Lancet;1:452-453, 1976. [↑](#endnote-ref-42)
43. Otto SJ, van Houwelingen AC, Antal M, Manninen A, Godfrey K, Lopez-Jaramillo P and Hornstra G. Maternal and neonatal essential fatty acid status in phospholipids: an international comparative study. Eur J Clin Nutr; 51, 232-242, 1997. [↑](#endnote-ref-43)
44. Holman RT. Control of polyunsaturated fatty acids in tissue lipids. J Am Coll Nutr;51:183–211, 1989. [↑](#endnote-ref-44)
45. Neuringer M, Connor WE, Lin DS, Barstad L. Biochemical and functional effects of prenatal and postnatal omega-3 fatty acid deficiency on retina and brain in monkeys. Proc Natl Acad Sci U S A;83:4021–4025, 1986. [↑](#endnote-ref-45)
46. Herrera E, Amusquivar E. Lipid metabolism in the fetus and the newborn, Diabetes/Metabolism Research and Reviews, 16, 3, 202-210, 2000. [↑](#endnote-ref-46)
47. Green P, Yavin E. Mechanisms of docosahexaenoic acid accretion in the fetal brain, Journal of Neuroscience Research; 52: 129-136, 1998. [↑](#endnote-ref-47)
48. Campbell FM, Gordon MJ, Dutta-Roy AK. Preferential uptake of long chain polyunsaturated fatty acids by isolated human placental membranes. Mol Cell Biochem 155:77–83, 1996. [↑](#endnote-ref-48)
49. Naval J, Calvo M, Laborda J, Dubouch P, Frain M, Sala-Trepat JM, Uriel J. Expression of mRNAs for alpha-fetoprotein (AFP) and albumin and incorporation of AFP and docosahexa-enoic acid in baboon fetuses. J Biochem (Tokyo) 111:649–654, 1992. [↑](#endnote-ref-49)
50. Benassayag C, Mignot TM, Haourigui M, Civel C, Hassid J, Carbonne B, Nunez EA, Ferre F. High polyunsaturated fatty acid, thromboxane A2, and alpha-fetoprotein concentrations at the human feto-maternal interface. J Lipid Res 38:276–286, 1997. [↑](#endnote-ref-50)
51. Haggarty P, Page K, Abramovich D, Ashton J. Polyunsaturated fatty acid accumulation in the perfused human placenta. Prostagland Leukot Essent Fatty Acids 57:232, 1997. [↑](#endnote-ref-51)
52. Marín MC, de Tomás ME, Serres C, and Mercuri O. Protein-energy malnutrition during gestation and lactation in rats affects growth rate, brain development and essential fatty acid metabolism. J Nutr 125, 1017-1024, 1995. [↑](#endnote-ref-52)
53. Marín MC, de Alaniz MJT. Relationship between dietary oil during gestation and lactation and biosynthesis of polyunsaturated fatty acids in control and in malnourished dam and pup rats. J Nutr Biochem; 9(7): 388-395, 1998. [↑](#endnote-ref-53)
54. Su HM, Huang MC, Saad NM, Nathanielsz PW, Brenna JT, Fetal baboons convert 18:3n-3 to 22:6n-3 in vivo. A stable isotope tracer study. J Lipid Res; 42(4):581-586, 2001. [↑](#endnote-ref-54)
55. Clandinin MT, Chappell JE, Leong S, Heim T, Swyer PR, Chance GW. Extrauterine fatty acid accretion in infant brain: implications for fatty acid requirements. Early Hum Dev;4:131–138, 1980. [↑](#endnote-ref-55)
56. Simopoulos AP, Leaf A, Salem N. Jr., Essentiality of and recommended dietary intakes for omega-6 and omega-3 fatty acids, Annals of Nutrition and Metabolism. 43(2)127-130, 1999. [↑](#endnote-ref-56)
57. Kris-Etherton PM, Taylor DS, Yu-Poth S, Huth P, Moriarty K, Fishell V, Hargrove RL, Zhao G, and Etherton TD. **Polyunsaturated fatty acids in the food chain in the United States** Am J Clin Nutr, 71:179-188, 2000. [↑](#endnote-ref-57)
58. Mercuri O, De Tomás ME, and Itarte H. Prenatal protein depletion and Δ9, Δ6 and Δ5 desaturases in the rat. Lipids 14, 822-825, 1979. [↑](#endnote-ref-58)
59. Koletzko B, Agostoni C, Carlson SE, Clandinin T, Hornstra G, Neuringer M, Uauy R, Yamashiro Y, Willatts P. Long-chain polyunsaturated fatty acids (LC-PUFA) and perinatal development. Acta Pediatr 460-464, 2001. [↑](#endnote-ref-59)
60. Robillard PY, Christon R. Lipid intake during pregnancy in developing countries. Possible effects of essential fatty acid deficiency on fetal growth. Prostaglandins Leukot Essent Fatty Acids, 48:139–142, 1993. [↑](#endnote-ref-60)
61. Sanders TAB. Essential fatty acid requirements of vegetarians in pregnancy, lactation, and infancy, Am J Clin Nutr; 70(3)555S-559S, 1999. [↑](#endnote-ref-61)
62. Hornstra G. Essential fatty acids in mothers and their neonates. Am J Clin Nutr;71:1262S-1269S, 2000 [↑](#endnote-ref-62)
63. Smit EN, Oelen EA, Seerat E, Muskiet FA, Boersma E. Breast milk docosahexaenoic acid (DHA) correlates with DHA status of malnourished infants, Arch Dis Child;82(6): 493-494, June 2000. [↑](#endnote-ref-63)
64. Hornstra G, Houwelingen VAC, Simonis M, Gerrard JM. Fatty acid composition of umbilical arteries and veins: possible implications for fetal EFA-status. Lipids; 24:511-517, 1990. [↑](#endnote-ref-64)
65. Al MDM, van Howellingen AC, Kester ADM. Maternal essential fatty acid patterns during normal pregnancy and its relationship with the neonatal essential fatty acid status. British J Nutr; 74:55-68, 1995. [↑](#endnote-ref-65)
66. Araya J, Rojas M, Fernandez P, Mataluna A. Diferencias en la composición porcentual de los poliinsaturados de cadena larga en eritrocitos materno-fetales en nacimientos de termino o pretermino en humanos. Arch Lationoamericanos de Nutricion; 48:210-215, 1998. [↑](#endnote-ref-66)
67. Al MDM, van Howellingen AC, Hornstra G. Relation between birth order and the maternal and neonatal docosahexaenoic acid status. Eur J Clin Nutr; 51:548-553, 1997. [↑](#endnote-ref-67)
68. van Houwelingen AC, Sorensen JD, Hornstra G, Simonis MM, Boris J, Olsen SF, Secher NJ. Essential fatty acid status in neonates after fish-oil supplementation during late pregnancy. Br J Nutr; 74(5):723-731, 1995. [↑](#endnote-ref-68)
69. Connor WE, Lowensohn R, Hatcher L. Increased docosahexaenoic acid levels in human new-born infants by administration of sardines and fish oil during pregnancy. Lipids; 31:S183–S187, 1996. [↑](#endnote-ref-69)
70. Velzing-Aarts FV, van der Klis FR, van der Dijs FP, van Beusekom CM, Landman H, Capello JJ, Muskiet FA. Effect of three low-dose fish oil supplements, administered during pregnancy, on neonatal long-chain polyunsaturated fatty acid status at birth. Prostaglandins Leukotrienes & Essential Fatty Acids.; 65 (1):51-57. 2001. [↑](#endnote-ref-70)
71. *Otto SJ, van Houwelingen AC, Hornstra G. The effect of supplementation with docosahexaenoic and arachidonic acid derived from single cell oils on plasma and erythrocyte fatty acids of pregnant women in the second trimester. Prostaglandins Leukot Essent Fatty Acids; 63 (5):323-8,2000.* [↑](#endnote-ref-71)
72. *Smuts CM, Huang M, Mundy D, Plasse T, Major S, Carlson SE. A randomized trial of docosahexaenoic acid supplementation during the third trimester of pregnancy. Obstet Gynecol; 101 (3):469-79,2003*. [↑](#endnote-ref-72)
73. Koletzko B, Braun M. Arachidonic acid and early human growth: is there a relation? Ann Nutr Metab; 35:128-131, 1991. [↑](#endnote-ref-73)
74. Crawford MA, Doyle W, Drury P, Lennon A, Costeloe K, Leighfield M. n-6 and n-3 fatty acids during early human development. J Intern Med; 225(1):59-169, 1989. [↑](#endnote-ref-74)
75. Ongari MA, Ritter JM, Orchard MA, Wadell KA, Blair IA, Lewis PJ. Correlation of prostacyclin synthesis by humans umbilical artery with status of essential fatty acid. Am J Obstet Gynecol; 149:455-460, 1984. [↑](#endnote-ref-75)
76. Foreman-van Drongelen MM, van Houwelingen AC, Kester AD, Hasaart TH, Blanco CE, Hornstra G. Long-chain polyunsaturated fatty acids in preterm infants: status at birth and its influence on postnatal levels. J Pediatr; 126(4):611-618, 1995. [↑](#endnote-ref-76)
77. Leaf AA, Leighfield MJ, Costeloe KL, Crawford MA. Long chain polyunsaturated fatty acids and fetal growth. Early Hum Dev; 30:183–191, 1992. [↑](#endnote-ref-77)
78. Woltil HA, van Beusekom CM, Schaafsma A, Muskiet FAJ, Okken A. Long-chain polyunsaturated fatty acid status and early growth of low birth weight infants. Eur J Pediatr; 157:146–152, 1998. [↑](#endnote-ref-78)
79. Rump P, Mesink RP, Kester ADM, Hornstra G. Essential fatty acid composition of plasma phospholipids and birth weight: a study in term neonates. Am J Clin Nutr; 73:797-806, 2001. [↑](#endnote-ref-79)
80. Grandjean P, Bjervec KS, Weihea P, and Steuerwald U. Birthweight in a fishing community: significance of

    essential fatty acids and marine food contaminants. Int J Epidemiol; 30:1272-1278, 2001. [↑](#endnote-ref-80)
81. Olsen SF, Hansen HS, Sommer S, Jensen B, Sorensen TIA, Secher NJ, Zachariassen P. Gestational age in relation to marine n-3 fatty acids in maternal erythrocytes: A study in the Faroe Islands and Denmark. Am J Obstet Gynecol 164:1203-1209, 1991. [↑](#endnote-ref-81)
82. Reece MS, Maternal and perinatal long-chain fatty acids: possible roles in preterm birth - Am J Obstet Gynecol; 176(4):907-914, Apr 1997. [↑](#endnote-ref-82)
83. Badart-Smook A, van Houwelingen AC, Al MDM, Kester ADM, Hornstra G. Fetal growth is associated positively with maternal intake of riboflavin and negatively with maternal intake of linoleic acid. J Am Diet Assoc; 97:867–870, 1997. [↑](#endnote-ref-83)
84. *Olsen SF, Secher NJ. Low consumption of seafood in early pregnancy as a risk factor for preterm delivery: prospective cohort study. BMJ; 324 (7335):447, 2002.* [↑](#endnote-ref-84)
85. Elias SL, Innis SM. Infants plasma *trans*. n-6, and n-3 fatty acids and conjugated linoleic acids are related to maternal plasma fatty acids, length of gestation, and birth weight and length. Am J Clin Nutr; 73:807-814, 2001. [↑](#endnote-ref-85)
86. Olsen SF. Secher NJ. A possible preventive effect of low-dose fish oil on early delivery and pre-eclampsia: indications from a 50-year-old controlled trial. Br J Nutr; 64(3):599-609, 1990. [↑](#endnote-ref-86)
87. Olsen SF, Sorensen JD, Secher NJ, Hedegaard M, Henriksen TB, Hansen HS, Grant A. Randomised controlled trial of effect of fish-oil supplementation on pregnancy duration. Lancet; 339:1003–1007, 1992. [↑](#endnote-ref-87)
88. Olsen SF. Hansen HS. Secher NJ. Jensen B. Sandstrom B. Gestation length and birth weight in relation to intake of marine n-3 fatty acids. Br J Nutr; 73(3):397-404, 1995. [↑](#endnote-ref-88)
89. Helland IB. Saugstad OD. Smith L. Saarem K. Solvoll K. Ganes T. Drevon CA. Similar effects on infants of n-3 and n-6 fatty acids supplementation to pregnant and lactating women. Pediatrics. 108(5):1-10, 2001. [↑](#endnote-ref-89)
90. Onwude JL, Lilford RJ, Hjartardottir H, Staines A, Tuffnell D. A randomised double blind placebo controlled trial of fish oil in high risk pregnancy. Br J Obstet Gynaecol; 102:95–100, 1995. [↑](#endnote-ref-90)
91. Olsen SF, Secher NJ, Tabor A, Weber T, Walker JJ, Gluud C. Randomised clinical trials of fish oil supplementation in high risk pregnancies. Br J Obstet Gynecol 107:382-395, 2000. [↑](#endnote-ref-91)
92. McGregor JA, Allen KGD, Harris MA, Reece M, Wheeler M, French JI and Morrison J. The Omega-3 Story: Nutritional prevention of preterm birth and other adverse pregnancy outcomes. Obstet Gynecol Survey; 56(6):S1-S13, 2001. [↑](#endnote-ref-92)
93. Maurage C. Guesnet P. Pinault M. Rochette de Lempdes J. Durand G. Antoine J. Couet C. Effect of two types of fish oil supplementation on plasma and erythrocyte phospholipids in formula-fed term infants. Biol Neonate. 74(6):416-429, 1998. [↑](#endnote-ref-93)
94. Carlson SE. Arachidonic acid status of human infants: influence of gestational age at birth and diets with very long chain n-3 and n-6 fatty acids. J Nutr; 126(4):1092S-1098S, 1996. [↑](#endnote-ref-94)
95. Koletzko B. Fatty acids and early human growth. Am J Clin Nutr; 73(4):671-2, 2001. [↑](#endnote-ref-95)
96. Borod E, Atkinson R, Barclay WR, Carlson SE. Effects of third trimester consumption of eggs high in docosahexaenoic acid on docosahexaenoic acid status and pregnancy. Lipids 34:S231, 1999. [↑](#endnote-ref-96)
97. Yonekubo A., Honda S., Okano M., Takahashi K., Yamamoto Y. Dietary fish oil alters rat milk composition and liver and brain fatty acid composition of fetal and neonatal rats. J. Nutr; 123:1703-1708, 1993. [↑](#endnote-ref-97)
98. Wainright P.E.; Huang Y.-S.; Bulman-Fleming B.; Levesque S.; McCutcheon D, The effect of dietary fatty acid composition combined with environmental enrichment on brain and behavior in mice, Behavioural Brain Research; 60(2):125-136, 1994. [↑](#endnote-ref-98)
99. Enslen M., Milon H., Malnoe A. Effect of low intake of n-3 fatty acids during development on brain phospholipids, fatty acid composition and exploratory behavior in rats. Lipids; 26:203-208, 1991. [↑](#endnote-ref-99)
100. Greiner R, Winter CS, Nathanielsz J, Brenna PW, Thomas J. Brain docosahexaenoate accretion in fetal baboons: bioequivalence of dietary [alpha]-Linolenic and Docosahexaenoic Acids. Pediatric Res;. 42(6):826-834, 1997. [↑](#endnote-ref-100)
101. Anderson GJ, Lin D, Neuringer M, Connor WE. The Reversal of Maternal N-3 Fatty Acid Deficiency In Newborn Monkey Infants: Effects Upon Blood, Retina and Brain, FASEB J, Experimental Biology 2001, Orlando, Florida, March 31 – April 4, 2001. [↑](#endnote-ref-101)
102. Neuringer M., Connor W. E., Lin D. S., Barstad L., Luck S. Biochemical and functional effects of prenatal and postnatal omega 3 fatty acid deficiency on retina and brain in rhesus monkeys. Proc Natl Acad Sci USA; 83:4021-4025, 1986. [↑](#endnote-ref-102)
103. Amusquivar, E., Rupérez, F. J., Barbas, C., Herrera, E. Low arachidonic acid rather than {alpha}-tocopherol Is responsible for the Delayed Postnatal Development in Offspring of Rats Fed Fish Oil Instead of Olive Oil during Pregnancy and Lactation. J Nutr; 130: 2855-2865, 2000. [↑](#endnote-ref-103)
104. Innis, SM, de la Presa Owens, S. (2001). Dietary Fatty Acid Composition in Pregnancy Alters Neurite Membrane Fatty Acids and Dopamine in Newborn Rat Brain. J Nutr; 131:118-122, 2001. [↑](#endnote-ref-104)
105. Salvati S, Attorri L, Avellino C, Di Biase A, Sanchez M. Diet, lipids and brain development, Developmental Neuroscience; 22(5-6):481-487, 2000. [↑](#endnote-ref-105)
106. Williams C, Birch EE, Emmett PM, Northstone K., Stereoacuity at age 3.5 y in children born full-term is associated with prenatal and postnatal dietary factors: a report from a population-based cohort study. Am J Clin Nutr;73(2):316-322, Feb 2001. [↑](#endnote-ref-106)
107. Cheruku SR, Montgomery-Downs HE, Farkas SL, Thoman EB, Lammi-Keefe CJ. Higher maternal plasma docosahexaenoic acid during pregnancy is associated with more mature neonatal sleep-state patterning. Am J Clin Nutr; 76 (3):608-613. 2002. [↑](#endnote-ref-107)
108. World Health Organization (WHO). Exclusive Breastfeeding for Six Months. <http://www.who.int/child-adolescent-health/NUTRITION/infant_exclusive.htm> [↑](#endnote-ref-108)
109. Schmeits BL, Content of lipid nutrients in the milk of Fulani women, J Hum Lact; 15(2): 113-120, June 1999. [↑](#endnote-ref-109)
110. Schmeits, BL, Cook JA, VanderJagt DJ, Magnussen MA, Bhatt SK, Bobik Jr EG, Huang Y-S and Glewwe RH. Fatty acid composition of the milk lipids of women in Nepal, Nutr Res; 19(9):1339-1348, 1999. [↑](#endnote-ref-110)
111. Krasevec JM, Jones PJ, Cabrera-Hernandez A, Mayer DL, Connor WE. Maternal and infant essential fatty acid status in Havana, Cuba. Am J Clin Nutr; 76 (4):834-844. 2002. [↑](#endnote-ref-111)
112. Makrides M, Neuman MA, Gibson R. Effect of maternal docosahexaenoic acid (DHA) supplementation on breast milk consumption. Eur J Clin Nutr; 50:352-357, 1996. [↑](#endnote-ref-112)
113. Gibson RA, Neuman MA, Makrides M. Effect of increasing breast milk docosahexaenoic acid on plasma and erythrocyte phospholipid fatty acid and neural indices of exclusively breast fed infants. Eur J Clin Nutr; 51:578-584, 1997. [↑](#endnote-ref-113)
114. Jensen CL, Maude M, Anderson RE, Heird W. Effect of docosahexaenoic acid supplementation of lactating women on the fatty acid composition of breast milk lipids and maternal and infant plasma phospholipids. Am J Clin Nutr; 71(suppl):292S-299S, 2000. [↑](#endnote-ref-114)
115. Hawkes JS, Bryan D-L, Makrides M, Neumann MA, Gibson RA. A randomized trial of supplementation with docosahexaenoic acid-rich tuna oil and its effects on the human milk cytokines interleukin 1{beta}, interleukin 6, and tumor necrosis factor {alpha}. Am J Clin Nutr; 75 (4):754-760. 2002. [↑](#endnote-ref-115)
116. DelPrado M, Villalpando S, Elizalde A, Rodriguez M, Demmelmair H, Koletzko B. Contribution of dietary and newly formed arachidonic acid to human milk in women eating a low fat diet. Am J Clin Nutr; 74:242-247, 2001. [↑](#endnote-ref-116)
117. Larque E, Demmelmair H, Koletzko B. Perinatal supply and metabolism of long-chain polyunsaturated fatty acids: importance for the early development of the nervous system. Annals of the New York Academy of Sciences.; 967:299-310. 2002. [↑](#endnote-ref-117)
118. *Helland IB. Saugstad OD. Smith L. Saarem K. Solvoll K. Ganes T. Drevon CA. Maternal supplementation with very-long-chain n-3 fatty acids during pregnancy and lactation augments children's IQ at 4 years of age. Pediatrics; 111 (1):e39-44, 2003.* [↑](#endnote-ref-118)
119. Carlson SE, Neuringer M, Reisbick S. Assessment of Infant Visual and Cognitive Function in relation to Long Chain Polyunsaturated Fatty Acid. Ed. Roche. Switzerland; 10-18, 1997. [↑](#endnote-ref-119)
120. Carlson SE, Rhodes PG, Ferguson MG. Docosahexaenoic status of preterm infants at birth and following feeding with human milk or formula. Am J Clin Nutr; 44:798-800, 1986. [↑](#endnote-ref-120)
121. Farquharson J, Cockburn F, Patrick WA, Jamieson EC, Logan RW. Infant cerebral cortex phospholipid fatty acid composition and diet. Lancet; 340:810-813, 1992. [↑](#endnote-ref-121)
122. Uauy R, Hoffman D, Peirnao P, Birch D, Birch E. Fatty acids in visual and brain development. Lipids; 36:885-895, 2001. [↑](#endnote-ref-122)
123. SanGiovanni JP, Parra-Cabrera MS, Coldits G, Dwyer J, Berky C. Meta-analysis of Dietary Essential Fatty Acids and Long-chain Polyunsaturated Fatty Acids as They Relate to Visual Acuity Development in Healthy Preterm Infants. Pediatrics; 6:1292-1298, 2000. [↑](#endnote-ref-123)
124. Marin MC, Rey GE, Rodrigo MA, de Alaniz MJ. Acidos grasos de fosfolipidos en plasma y eritrocitos de lactantes desnutridos alimentados con leche materna o formulas. Medicina; 61:41-48, 2001. [↑](#endnote-ref-124)
125. Simmer, K. Long chain polyunsaturated fatty acid supplementation in infants born at term. Cochrane Neonatal Group Cochrane Database of Systematic Reviews. Issue Issue 1, 2002. [↑](#endnote-ref-125)
126. Agostini C, Trojan S, Bellu R, Riva E, Giovannini M. Neurodevelopmental quotient of healthy term infants at 4 months and feeding practice: the role of long-chain polyunsaturated fatty acids. Pediatr Res; 38:262-266, 1995. [↑](#endnote-ref-126)
127. Makrides M, Neumann MA, Simmer K, Gibson RA. Dietary long-chain polyunsaturated fatty acids do not influence growth of term infants: a randomised clinical trial. Pediatrics; 104:468-475, 1999. [↑](#endnote-ref-127)
128. Makrides M, Neumann MA, Simmer K, Gibson RA. A critical appraisal of the role of long-chain polyunsaturated fatty acids on neural indices of term infants: a randomised controlled trial. Pediatrics; 105:32-38, 2000. [↑](#endnote-ref-128)
129. Scott DT, Janowsky JS, Carroll RE, Taylor JA, Auestad N, Montalto MB. Formula supplementation with long-chain polyunsaturated fatty acids: are there developmental benefits? Pediatrics;102(5):E59,1998. [↑](#endnote-ref-129)
130. Lucas A, Stafford M, Morley R, Abbott R, Stephenson T, Macfadyen U, Elias-Jones A, Clements H. Efficacy and safety of long-chain polyunsaturated fatty acid supplementation of infant-formula milk: a randomized trial. Lancet;354:1948-1954,1999. [↑](#endnote-ref-130)
131. Birch E, Garfield S, Hoffman DH, Uauy R, Birch D. A randomized controlled trial of early dietary supply of long chain polyunsaturated fatty acids and mental development in term infants. Developmental Medicine and Child Neurology; 42:174-181, 2000. [↑](#endnote-ref-131)
132. Birch EE, Hoffman DR, Castaneda YS, Fawcett SL, Birch DG, Uauy RD. A randomized controlled trial of long-chain polyunsaturated fatty acid supplementation of formula in term infants after weaning at 6 wk of age. Am J Clin Nutr.; 75 (3):570-580. 2002 [↑](#endnote-ref-132)
133. Voigt RG, Jensen CL, Fraley JK, Rozelle JC, Brown FR, 3rd, Heird WC. Relationship between omega3 long-chain polyunsaturated fatty acid status during early infancy and neurodevelopmental status at 1 year of age. J Hum Nutr Dietet.; 15 (2):111-120. 2002 [↑](#endnote-ref-133)
134. Salem N, Jr., Litman B, Kim HY, Gawrisch K. Mechanisms of action of docosahexaenoic acid in the nervous system. Lipids.; 36 (9):945-959. 2001.. [↑](#endnote-ref-134)
135. Ramakrishnan U**,** Latham MC, Abel R. Vitamin A supplementation does not improve growth of preschool children: A randomized double blind field trial in South India. J Nutr; 125(2):202-211, 1995. [↑](#endnote-ref-135)
136. Ramakrishnan U, Latham MC, Abel R. Vitamin A supplementation and morbidity among preschool children in South India. Amer J Clin Nutr; 61:1295-303, 1995. [↑](#endnote-ref-136)
137. Ramakrishnan U, Neufeld L. Recent Advances in Nutrition and Intrauterine Growth. In: Martorell R., Haschke F., Eds. Nutrition and Growth. Nestlé Nutrition Workshop Series, Vol 47. Philadelphia, Lippincott-Williams and Wilkins; Chapter 7, 2001. [↑](#endnote-ref-137)
138. Ramakrishnan U, Martorell R. The Role of Vitamin A in Reducing Mortality and Morbidity and Improving Growth in Young Children. Salud Publica de Mexico 40(2):189-198, 1998. [↑](#endnote-ref-138)
139. Ramakrishnan U, Gonzales-Cossio T, Neufeld LM, Rivera J, Martorell R. Effect of multiple micronutrient supplements during pregnancy on birth size: a randomized double blind clinical trial in semi-rural Mexico. *Am J Clin Nutr 77(3):720-725, 2003.* [↑](#endnote-ref-139)
140. Ramakrishnan U, Neufeld, LM, Gonzales-Cossio T, Rivera J, Martorell R. Effect of multiple micronutrient supplements during pregnancy on maternal weight and skinfold changes: A randomized double blind clinical trial in Mexico. Annual Meeting of the American Public Health Association, Atlanta, Oct 2001. [↑](#endnote-ref-140)
141. Neufeld LM, Ramakrishnan U, Rivera J, Villalpando S, Gonzalez-Cossio T, Martorell R. Prevalence of anemia and iron deficiency during pregnancy of women supplemented with iron or iron and multiple micronutrients. FASEB J; 15(4): Abst #505.2, 2001. [↑](#endnote-ref-141)
142. Neufeld LM, Ramakrishnan U, Gonzales-Cossio T, Rivera J, Martorell R. Prevalence of multiple micronutrient malnutrition during pregnancy in a semi-rural community in Mexico. International Union of Nutritional Sciences (IUNS) Congress, Vienna, Austria, August 27- Sept 1, 2001. [↑](#endnote-ref-142)
143. Hernandez-Cordero S, Rivera J, Villalpando S, Gonzalez-Cossio T, Neufeld LM, Ramakrishnan U, Martorell R. Multiple Micronutrient Supplementation During Pregnancy: Effect on Breast Milk Retinol Concentration at One Month Postpartum. FASEB J. 15(4): Abst #505.7; 2001. [↑](#endnote-ref-143)
144. Garcia-Giuerra A, Rivera-Dommarco J, Neufeld LM, Gonzalez-Cossio T, Ramakrishnan U, Martorell R. Cord blood zinc concentrations from women supplemented with iron or iron and multiple micronutrients during pregnancy. FASEB J. 15(4): Abst #505.4, 2001. [↑](#endnote-ref-144)
145. Bayley Scales of Infant Development, 2nd ed. Psychological Corporation, San Antonio, TX, 1993. [↑](#endnote-ref-145)
146. *Neufeld LM, U Ramakrishnan U, González-Cossío T, Rivera J, Martorell R. Cambios en la prevalencia de anemia de 3 a 12 meses de edad en una población de niños suplementados. Oral presentation at the X Congreso de Investigación en Salud Publica, March 2003, Cuernavaca, Mexico. Abstract #: TL186.*  [↑](#endnote-ref-146)
147. Ramakrishnan U. Functional consequences of Nutritional Anemia During Pregnancy and Early Childhood. In: Nutritional Anemias. Ed: Ramakrishnan U. Boca Raton, FL, CRC Press; 43-68, 2001. [↑](#endnote-ref-147)
148. Ramakrishnan U, Yip R. Experiences and Challenges in Industrialized Countries: Control of Iron Deficiency in Industrialized Countries. *J Nutr. 132(4S):820S-824S, 2002*. [↑](#endnote-ref-148)
149. Frith-Terhune, A, Cogswell M, Kettel-Khan L, Will J, Ramakrishnan U*.* Determinants of iron deficiency among Mexican-American and Non-Hispanic White Females: Third National Health and Nutrition Examination Survey, 1988-94, (NHANES III). Am J Clin Nutr; 72(4):963-968, 2000. [↑](#endnote-ref-149)
150. *Cogswell, M, Kettel-Khan L, Ramakrishnan, U. Iron supplement among women in the United States: Science, Policy, and Practice. J Nutr 133;1974-1977, 2003.* [↑](#endnote-ref-150)
151. *Li H, Stein AD, Barnhart HX, Torun B, Ramakrishnan U, Martorell R.* ***Associations between prenatal and postnatal growth and adult body size and composition.*** *Am J Clin Nutr 77:1498–1505, 2003*. [↑](#endnote-ref-151)
152. Stein A, Conlisk AJ, Ramakrishnan U**,** Schroeder DG, Torun B, Martorell R. Nutritional Supplementation and adult fat and fat-free mass in young Guatemalans. FASEB J. 15(4): Abst #223.5, 2001. [↑](#endnote-ref-152)
153. Ramakrishnan U, Barnhart H, Schroeder DG, Stein AD, Martorell R. Early childhood nutrition, education and fertility milestones in Guatemala. J Nutr 129:2196-2202, 1999. [↑](#endnote-ref-153)
154. ? Ramakrishnan U, Martorell, R, Schroeder DG, Flores R. Intergenerational effects on linear growth. J Nutr; 129 (2):544‑549, 1999*.* [↑](#endnote-ref-154)
155. Kuklina EV, Ramakrishnan U, Martorell R. Predictors of Motor and Mental Development at 2 years of age in Rural Guatemala. FASEB J. 15(4): Abst #502.12, 2001. [↑](#endnote-ref-155)
156. Hernández-Avila M, Romieu I, Parra-Cabrera S, Hernández-Avila JE, Madrigal H, Willett W. Validity and Reproducibility of a Food Frequency Questionnaire to Assess Dietary Intake in Women Living in Mexico City. Salud Pub Mexico; 40:122-140, 1998. [↑](#endnote-ref-156)
157. Parra MS, Schnaas L, Meydani M, Romieu I, Perroni E, Martínez S. Erythrocyte phospholipid levels compared against reported dietary intake of polyunsaturated fatty acids in Pregnant Mexican women. *Pub Hlth Nutr J; 5 (6A):931-7, 2002.* [↑](#endnote-ref-157)
158. Parra MS, Schnaas L, Meydani M, Romieu I, Perroni, Martínez S. “Relationship between maternal erythrocyte polyunsaturated fatty acids and brainstem auditory evoked potentials (BAEP) in Mexican full term infants”. 17^th^ International Congress of Nutrition 2001. Abstract 1.08.016, pag. 93. 27-31 August 27-31th, 2001.Austria, Vienna. [↑](#endnote-ref-158)
159. Parra MS, Resa P, Schnaas L, Reza S, Romieu I, Perroni E. “Association between maternal polyunsaturated fatty acids and their infants visual acuity at 3 months of age”. Abstract No. TL 188, pag. 84. IX National Research Congress in Public Health. National Institute of Public Health. March 5th-7th 2001, Cuernavaca, Morelos México. [↑](#endnote-ref-159)
160. *DiGirolamo A, Neufeld L, Rivera J, Ramakrishnan U, Martorell R. (2003). Depression, Parenting Stress, and Social Support Among Women in Mexico. Published abstract from poster presentation at the American Psychological Association Meeting, August 2003, Toronto, Canada.* [↑](#endnote-ref-160)
161. *DiGirolamo A, Neufeld LM, Rivera J, Gonzalez-Cossio T, Ramakrishnan U, Martorell, R. (2003). Medidas de desarrollo motor en estudios de nutricion de campo. Oral presentation at the X Congreso de Investigación en Salud Publica, March 2003, Cuernavaca, Mexico. Abstract #: TL 184.* [↑](#endnote-ref-161)
162. *DiGirolamo AM, Neufeld L, Rivera J, Ramakrishnan U, Gonzalez de Cossio T, Martorell R. (2003). Nutritional predictors of gross motor milestones among children in Mexico. Poster presentation at Experimental Biology, April 2003, San Diego, CA. Abstract #: 438.7.* [↑](#endnote-ref-162)
163. Conlisk AJ, Stein AD, Schroeder DG, Torun B, Grajeda R, Martorell R. Determinants of fasting glucose in young Guatemalan adults. Ethnic Dis; 11:585-597, 2001. [↑](#endnote-ref-163)
164. Torun B, Stein AD, Schroeder DG, Grajeda R, Rodriguez M, Conlisk AJ, Mendez H, Martorell R. Migration and cardiovascular disease risk factors in young Guatemalan adults Int J Epidemiol;31:218-226, 2002. [↑](#endnote-ref-164)
165. Stein AD, Shea S, Basch CE, Contento IR, Zybert P. Variability and tracking of nutrient intakes of preschool children based on multiple administrations of the 24-hour dietary recall over three years. Am J Epidemiol;134:1427-1437, 1991. [↑](#endnote-ref-165)
166. Stein AD, Shea S, Basch CE, Contento IR, Zybert P. Consistency of the Willett semi-quantitative food frequency questionnaire and 24-hour dietary recalls in estimating nutrient intakes of preschool children. Am J Epidemiol;135:667-677, 1992. [↑](#endnote-ref-166)
167. Rodríguez MM, Méndez H, Torún B, Schroeder D, Stein AD. Validation of a semi-quantitative food frequency questionnaire for use among adults in Guatemala*. Publ Health Nutr;5:691-698, 2002.* [↑](#endnote-ref-167)
168. ? Stein AD, Shea S, Basch CE, Zybert P. Blood pressure reactivity does not correlate with baseline blood pressure or blood pressure change over time in preschool children. Am J Epidemiol; 136:795-805, 1992. [↑](#endnote-ref-168)
169. Stein AD, Shea S, Basch CE, Contento IR, Zybert P. Assessing change in nutrient intakes of preschool children: Comparing 24-hour dietary recall and food frequency methods. Epidemiology; 5:109-115, 1994. [↑](#endnote-ref-169)
170. Shea S, Stein AD, Basch CE, Lantigua R, Maylahn C, Strogatz D, Novick L. Independent associations of educational attainment and ethnicity with behavioral risk factors for cardiovascular disease. Am J Epidemiol; 134:567-582, 1991. [↑](#endnote-ref-170)
171. Shea S, Melnik TA, Stein AD, Zansky SM, Maylahn C, Basch CE. Age, sex, educational attainment and race/ethnicity in relation to consumption of specific foods contributing to the atherogenic potential of diet. Prev Med; 22:203-218, 1993. [↑](#endnote-ref-171)
172. Stein AD, Mincheva V, Stoyanovsky V, Dimitrov E, Hodjeva D, Petkov A, Tsanova V. Prevalence of risk factors for cardiovascular disease in a working Bulgarian population. CVD Prevention; 1:217-224, 1998. [↑](#endnote-ref-172)
173. Stein AD, Stoyanovsky V, Mincheva V, Dimitrov E, Hodjeva D, Petkov A, Tsanova V. Prevalence, awareness, management, and control of hypertension in a working Bulgarian population. Eur J Epidemiol: in press. [↑](#endnote-ref-173)
174. Stein AD, Ravelli ACJ, Lumey LH. Famine, third-trimester weight gain and intrauterine growth retardation: the Dutch Famine Birth Cohort Study. Hum Biol; 67:135-149, 1995. [↑](#endnote-ref-174)
175. Lumey LH, Stein AD, Ravelli ACJ. Maternal recall of birthweights of adult children: validation by hospital and Well Baby clinic records. Int J Epidemiol; 23:1006-1012, 1994. [↑](#endnote-ref-175)
176. Lumey LH, Stein AD, Ravelli ACJ. Timing of prenatal starvation in women and birth weight in their offspring: the Dutch famine birth cohort study. Eur J Obstet Gynecol Repr Biol; 61:23-30, 1995. [↑](#endnote-ref-176)
177. Lumey LH, Stein AD. In utero exposure to famine and subsequent fertility: The Dutch Famine Birth Cohort Study. Am J Public Health; 87:1962-1966, 1997. [↑](#endnote-ref-177)
178. Stein AD, Lumey LH. The association of maternal and offspring birth weights under conditions affecting maternal birth weight: The Dutch Famine Birth Cohort Study. Hum Biol; 72:641-654, 2000. [↑](#endnote-ref-178)
179. Villalpando S, Del Prado M. Interrelationship among dietary energy and fat intakes, maternal body fatness and milk total lipids. J Mamm Gland Biol Neoplasia, 4: 285-294, 1999. [↑](#endnote-ref-179)
180. Del Prado M, Delgado G, Villalpando S. Maternal lipid intake during pregnancy and lactation alters milk composition and production and litter growth in rats. J Nutr 127: 458-462, 1997. [↑](#endnote-ref-180)
181. Del Prado M, Villalpando S, Gordillo J, Hernández- Montes H. A high dietary lipid intake during pregnancy and lactation enhances mammary gland lipid uptake and lipoprotein lipase activity in rats. J. Nutr. 129: 1574-1578, 1999. [↑](#endnote-ref-181)
182. Villalpando S, DelPrado M, Lance A, Alfonso E, Rodríguez M, Demmelmair H, Koletzko B. 13C linoleic acid oxidation and transfer into milk in stunted lactating women with contrasting body mass index. Am J Clin Nutr; 74:827-32, 2001. [↑](#endnote-ref-182)
183. Villalpando S, DelPrado M, Stafford J, Delgado G. Diurnal variations in the fatty acid composition of milk fat from marginally nourished women. Arch Med Res 26:1995;139-143. [↑](#endnote-ref-183)
184. Stafford J, Villalpando S, Urquieta B. Circadian variation and changes after a meal in volume and lipid production in human milk from rural mexican women. Ann Nutr Metab 38: 232-237, 1994. [↑](#endnote-ref-184)
185. Del Prado M, Hernández H, Villalpando S. Characterization of a fluorometric method for lipoprotein lipase. Arch Med Res; 25: 331-335, 1994. [↑](#endnote-ref-185)
186. Kuklina E, Ramakrishnan,U, Martorell R.. Birth Size as a Predictor of Global Motor and Mental Development at 6 months of age in Rural Guatemala, Poster, Experimental Biology 2002, Faseb J; 16(4): Abst # 221.17, 2002. [↑](#endnote-ref-186)
187. Graff M, Kuklina E, Ramakrishnan U, Martorell R. Birth Size as a Predictor of Time until an Infant Can Sit without Support in Rural Guatemala. FASEB J. 16(4): Abst #221.23, 2002. [↑](#endnote-ref-187)
188. Moher D. Schulz KF. Altman DG. Lepage L. The CONSORT statement: revised recommendations for improving the quality of reports of parallel-group randomised trials. Lancet. 357(9263):1191-4, 2001. [↑](#endnote-ref-188)
189. *Encuesta Nacional de Nutrición 1999, Instituto Nacional de Salud Publica, Mexico (Unpublished data).*  [↑](#endnote-ref-189)
190. Gottlieb G. Developmental Psychobiological Theory. In:Developmental Science. Cairns RB, and Elder GH eds., pp. 63-77. Cambridge studies in Social and Emotional Development, Cambridge University Press, 1998, New York [↑](#endnote-ref-190)
191. Singer LT. Methodological considerations in longitudinal studies of infant risk. In: Dobbing J, ed. Developing Brain and Behaviour: The Role of Lipids in Infant Formula, San Diego: Academic Press, 209-251, 1997. [↑](#endnote-ref-191)
192. Cohen J. Statistical Power Analysis for the Behavioral Sciences, Second Edition. Lawrence Erlbaum Associates, Publishers, Hillsdale, New Jersey, 1988. [↑](#endnote-ref-192)
193. Apgar V. A proposal for a new method of evaluation of the newborn infant. From Current Researchers in Anesthesia and Analgesia, July - August, 1953, page 260. Presented before the Twenty-Seventh Annual Congress of Anesthetists, Joint Meeting of the International Anesthesia Research Society and the International College of Anesthetists, Virginia Beach, Virginia, September 22-25, 1952. [↑](#endnote-ref-193)
194. Medical College of Georgia. (1996). Assessing newborns' vital signs-The Apgar Score. Medical College of Georgia website, copyright 2002. [Http://www.mcg.edu/News/96features/Apgarscore.html](http://www.mcg.edu/News/96features/Apgarscore.html). [↑](#endnote-ref-194)
195. Brazelton TB, Nugent JK. Neonatal Behavioral Assessment Scale, 3rd Edition. London, England: Mac Keith Press, 1995. [↑](#endnote-ref-195)
196. Carlson SE. Behavioral methods used in the study of long-chain polyunsaturated fatty acid nutrition in primate infants, Am J Clin Nutr; 71:268S-274S, 2000. [↑](#endnote-ref-196)
197. *Bouglé D, Denise P, Vimard F, Nouvelot A, Penniello MJ, Guillois B. Early neurological and neurophysiological development of the preterm infant and polyunsaturated fatty acid supply. Clinical Neurophhysiology 1999; 110:1363-1370.* [↑](#endnote-ref-197)
198. Fagan JF, Detterman, DK. The Fagan Test of Infant Intelligence: A technical summary. J Appl Dev Psychol; 13:173-193, 1992. [↑](#endnote-ref-198)
199. Fagan JF III, Singer LT, Montie JE, Shepherd PA. Selective screening device for the early detection of normal or delayed cognitive development in infants at risk for later retardation. Pediatrics; 78:1021–1026, 1987. [↑](#endnote-ref-199)
200. Fagan JF, Singer LT. Infant recognition memory as a measure of intelligence. In: Lipsitt LP, ed. Advances in infancy research. Vol 2. Norwood, NJ: Ablex; 31–72, 1983. [↑](#endnote-ref-200)
201. Bornstein MH, Sigman MD. Continuity in mental development from infancy. Child Dev; 57:251-274, 1986. [↑](#endnote-ref-201)
202. McCall R, Carriger M. A meta-analysis of infant habituation and recognition memory performance as predictors of later IQ. Child Dev; 64:57-79, 1993. [↑](#endnote-ref-202)
203. Lohman TG, Roche AF, Martorell R. Anthropometric Standardization Reference Manual. Human Kinetics Publishers. Champaign, IL, 1988. [↑](#endnote-ref-203)
204. Hamill PVV, Drizd TA, Johnson CL, Reed RB, Roche AF, Moore WM. Physical growth: National Center for Health Statistics percentiles. Am J Clin Nutr; 32:607–629, 1979. [↑](#endnote-ref-204)
205. Ballard JL, Khoury JC, Wedig K, Wang L, Eilers-Walsman BL, Lipp R. New Ballard Score, expanded to include extremely premature infants. J Pediatr; 119:417-423, 1991. [↑](#endnote-ref-205)
206. Hornstra G. Essential fatty acids, pregnancy, and pregnancy complication: a round table discussion. In Sinclair A and Gibson R, eds, Essential fatty acids and eicosanoids. Champaign, IL: American Oil Chemists’ Society; 177-182, 1992. [↑](#endnote-ref-206)
207. Folch J, Lees M, Sloanne-Stanley GH. A simple method for the isolation and purification of total lipids from animal tissues. J Biol Chem; 226:497-509, 1957. [↑](#endnote-ref-207)
208. Kankaanpaa, P. 1. Nurmela, K. 2. Erkkila, A. 3. Kalliomaki, M. 4. Holmberg-Marttila, D. 5. Salminen, S. 1. Isolauri, E. 4. Polyunsaturated fatty acids in maternal diet, breast milk, and serum lipid fatty acids of infants in relation to atopy. Allergy. 56(7):633-638, 2001. [↑](#endnote-ref-208)
209. Gibson RA. Kneebone GM. Effect of sampling on fatty acid composition of human colostrum. J Nutr; 110(8):1671-5, 1980. [↑](#endnote-ref-209)
210. Harzer G. Haug M. Dieterich I. Gentner PR. Changing patterns of human milk lipids in the course of the lactation and during the day. Am J Clin Nutr; 37(4):612-21, 1983. [↑](#endnote-ref-210)
211. Makrides M. Simmer K. Neumann M. Gibson R. Changes in the polyunsaturated fatty acids of breast milk from mothers of full-term infants over 30 wk of lactation. Am J Clin Nutr; 61(6):1231-3, 1995. [↑](#endnote-ref-211)
212. Fédération Internationale de Laiterie - International Dairy Federation. Determination of fat. 1C:1-8, 1987. [↑](#endnote-ref-212)
213. Christie WW. A simple procedure for rapid transmethylation of glycerolipids and cholesteryl esters. J Lipid Res; 23:1072-1075, 1982. [↑](#endnote-ref-213)
214. *Valverde V, Delgado H, Martorell R, Belizan J, de Ramirez E, Pivaral VM, and Klein RE. The Measurement of Individual Food Intake in Longitudinal Nutrition Studies in Poor Rural Communities in Guatemala. Monograph 14. Guatemala: Institute of Nutrition of Central America and Panama (INCAP), 1980. 80 pages.* [↑](#endnote-ref-214)
215. *US Department of Agriculture, Agricultural Research Service. USDA Nutrient Database for Standard Reference, Release 14 (online). Available at Nutrient Data Laboratory Home Page,* [*http://www.nal.usda.gov/fnic/foodcomp*](http://www.nal.usda.gov/fnic/foodcomp)*, 2001.* [↑](#endnote-ref-215)
216. Raven JC, Court JH, & Raven J. Manual for Raven’s Progressive Matrices and Vocabulary Scales (Section 3) –Standard Progressive Matrices (1983 edition). Lewis: London, 1983. [↑](#endnote-ref-216)
217. Sattler JM Assessment of Children, Third Edition. San Diego, California, 1988. [↑](#endnote-ref-217)
218. Bradley R, Caldwell B. Home environment, cognitive competence and IQ among males and females. Child Dev; 51:1140-8, 1980. [↑](#endnote-ref-218)
219. Siegal LA. Home environmental influences on cognitive development in pre-term and full term children during the first 5 years. In: Gottfried AW, ed. Home Environment and Early Cognitive Development: Longitudinal Research. Orlando, FL: Academic Press, 197-233, 1990. [↑](#endnote-ref-219)
220. Caldwell B, Bradley R. Home Observation for Measurement of the Environment. Little Rock, AK: University of Arkansas, 1984. [↑](#endnote-ref-220)
221. Bradley R, Caldwell B. Using the HOME inventory to assess the family environment. Pediatric Nursing ; 14, 97-102, 1988. [↑](#endnote-ref-221)
222. Elardo R, Bradley R. The home observation for measurement of the environment: A review of research. Developmental Review; 1, 113-145, 1981. [↑](#endnote-ref-222)
223. Anderson JW, Johnstone BM, Remley DT. Breast-feeding and cognitive development: a meta-analysis., Am J Clin Nutr; 70(4):525-535, 1999. [↑](#endnote-ref-223)
224. World Health Organization. Indicators for assessing breastfeeding practices. Indicators for assessing breastfeeding practices. Geneva, Switzerland: World Health Organization, WHO/CHD/SER/91.4,1991. [↑](#endnote-ref-224)
225. Shrout PE. **Measurement reliability and agreement in psychiatry.** Stat Methods Med Res;7(3):301-317, 1998. [↑](#endnote-ref-225)
226. Spiegelman D, McDermott A, Rosner B. **Regression calibration method for correcting measurement-error bias in nutritional epidemiology.** Am J Clin Nutr;65(4 Suppl):1179S-1186S, 1997. [↑](#endnote-ref-226)
227. Rosner B, Willett WC. **Interval estimates for correlation coefficients corrected for within-person variation: implications for study design and hypothesis testing.** Am J Epidemiol;127(2):377-386, 1988. [↑](#endnote-ref-227)
228. Little RJA and Rubin DB. Statistical Analysis with Missing Data, second edition, Wiley:New York, 2002. [↑](#endnote-ref-228)
229. SAS Institute Inc. SAS/STAT Users Guide Version 6, Cary, NC. SAS Institute, 1990. [↑](#endnote-ref-229)
230. Zeger SL, Liang K-Y. Longitudinal data analysis for discrete and continuous outcomes. Biometrics; 42:121-130, 1986. [↑](#endnote-ref-230)
231. ?Zeger SL, Liang K-Y, Albert P.S. Models for longitudinal data: a generalized estimating equation approach. Biometrics; 44:1049-1060, 1988. [↑](#endnote-ref-231)
232. SAS Technical Report P-229. SAS Institute Inc., Cary, NC. 287-369, 1992. [↑](#endnote-ref-232)
233. Biddle BJ, Marlin MM. Causality, confirmation, credulity and structural equation modelling . Child Dev; 58:4-17, 1987. [↑](#endnote-ref-233)
234. Bentler PM, Stein JA. Structural equation models in medical research. Stat Meth Med Res; 1:159-181, 1992. [↑](#endnote-ref-234)
235. Johnston J. Econometric Methods. Third Edition. New York:McGraw-Hill Book Company, 1984. [↑](#endnote-ref-235)
236. Angrist JD, Imbens GW, Rubin DB. Identification of causal effects using instrumental variables. J Am Stat Ass, 91:444-455, 1996. [↑](#endnote-ref-236)
237. Farrell AD. Structural equation modeling with longitudinal data: strategies for examining group differences and reciprocal relationships. J Cons Clin Psych; 62:477-487, 1994. [↑](#endnote-ref-237)
238. Snedecor G, Cochran W. Statistical Methods. Iowa State University Press; 104, 1989. [↑](#endnote-ref-238)
239. Kleinbaum DG, Kupper LL, Muller KE. Applied Regression Analysis and other Multivariate Methods. 2nd, Ed. PWS-Kent Publishing Company. Boston, 1988.

     **H. CONSORTIUM / CONTRACTUAL ARRANGEMENTS**

     This is a collaborative project between the Rollins School of Public Health of Emory University, the Instituto Nacional de Salud Publica (INSP), and the Instituto Mexicano de Seguro Social, Cuernavaca, Mexico. Dr. Ramakrishnan (Principal Investigator) at Emory University will play a major role in developing the study protocol, providing logistical, epidemiological and technical support for the intervention trial, and will coordinate and participate in the analyses, interpretation and writing the results. Dr. Socorro Parra (Co-Principal Investigator), Senior Investigator, Department of Health and Nutrition, INSP will participate in the development of the study protocol, supervise and coordinate all administrative and technical aspects of the study, especially at the study site in Mexico and will participate in the analyses, interpretation and writing the results. Dr, Juan Rivera, Director, Center for Nutrition and Population Sciences, INSP will serve as an advisor and provide support in administrative and technical aspects of the study. Both institutions have signed an inter-institutional agreement to foster collaborative research (*Appendix IV*), and as part of this arrangement, Dr. Rivera is an Adjunct Associate Professor at the School of Public Health, Emory University. The resources of INSP include specialized laboratories for handling and analyzing biological products, professional personnel specialized in epidemiologic studies, and technical personnel trained in data collection, management and analysis. The intervention trial will be carried out in IMSS, General Hospital I, Cuernavaca, Morelos (see Appendix I for letter of collaboration) and has an institutional agreement with [↑](#endnote-ref-239)
